# Schmutzi: estimation of contamination and endogenous mitochondrial consensus calling for ancient DNA

**DOI:** 10.1186/s13059-015-0776-0

**Published:** 2015-10-12

**Authors:** Gabriel Renaud, Viviane Slon, Ana T. Duggan, Janet Kelso

**Affiliations:** Max Planck Institute for Evolutionary Anthropology, Deutscher Platz 6, Leipzig, Germany; McMaster Ancient DNA Centre, Department of Anthropology, McMaster University, 1280 Main St West, Hamilton, ON, L8S 4L9 Canada

## Abstract

Ancient DNA is typically highly degraded with appreciable cytosine deamination, and contamination with present-day DNA often complicates the identification of endogenous molecules. Together, these factors impede accurate assembly of the endogenous ancient mitochondrial genome. We present schmutzi, an iterative approach to jointly estimate present-day human contamination in ancient human DNA datasets and reconstruct the endogenous mitochondrial genome. By using sequence deamination patterns and fragment length distributions, schmutzi accurately reconstructs the endogenous mitochondrial genome sequence even when contamination exceeds 50 %. Given sufficient coverage, schmutzi also produces reliable estimates of contamination across a range of contamination rates. Availability: https://bioinf.eva.mpg.de/schmutzi/ license:GPLv3.

**Electronic supplementary material:**

The online version of this article (doi:10.1186/s13059-015-0776-0) contains supplementary material, which is available to authorized users.

## Introduction

Advances in sequencing and improved methods for the extraction of ancient DNA (aDNA) have enabled the study of ancient genomes. However, many computational hurdles remain in the analysis of aDNA. After the death of an organism, the endogenous DNA begins to degrade and accumulates chemical damage. aDNA molecules, therefore, tend to be quite short, typically less than 60 bases in length [1], and carry uracils as a result of cytosine deamination. Deaminated cytosines are misread as thymines during sequencing and lead to the characteristic increase in frequency of cytosine to thymine transitions near the ends of ancient molecules [2]. Further, when extracting DNA from ancient human remains, microbial DNA often forms the bulk of all recoverable fragments [3], which, together with contaminating DNA from individuals who handled the ancient sample, is sequenced along with the endogenous DNA [4]. While bacterial sequences do not typically align to the human reference genome, present-day human contaminants will align together with the endogenous DNA fragments. The presence of contaminant fragments affects both consensus calling and genotyping, and the resulting errors may influence comparisons to present-day humans including the calculations of genotype likelihoods, divergence times, population genetics parameters and phylogenetic reconstructions [5, 6].

**Fig. 1:**
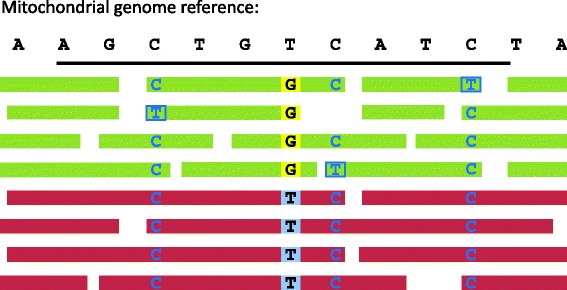
Schematic illustration of mitochondrial sequences from an ancient DNA library. When DNA from an ancient human sample is sequenced, DNA from the ancient human (endogenous fragments represented in *green*) as well as contaminant DNA fragments from the individuals who have handled the bone (contaminating fragments represented in *red*) are included. Because DNA undergoes deamination over time, endogenous fragments are likely to carry deaminated cytosines (represented as *T’s* in a *blue frame*), particularly near the ends of the DNA fragments. The non-deaminated cytosines are represented as *unframed blue C’s*. Schmutzi first identifies the endogenous fragments and, in a second step, uses these to quantify contamination. These steps are repeated until convergence is achieved and a single mitochondrial genome is identified

Previous approaches to reconstructing ancient mitochondrial genomes include the mapping iterative assembler (MIA), which iteratively calls a consensus from the DNA fragments [7]. When contamination is high (e.g., >30 %), calling the consensus sequence of the endogenous mitochondrial genome without removing contaminant fragments is likely to result in an incorrect sequence (see Fig. 1). Because ancient endogenous DNA is more likely to be deaminated than the contaminant DNA from present-day humans [8], some studies have restricted the analyses to fragments carrying deaminated cytosines [9, 10]. However, using only deaminated fragments reduces the amount of data available for many ancient samples.

Due to these issues, research groups have generally prioritized samples with low levels of present-day human contamination. To date, methods to quantify present-day human mitochondrial contamination have relied on the presence of fixed differences between the mitochondrial genomes of archaic and modern humans [11, 12]. This works well when analyzing the genomes of Neanderthals and Denisovans, but early modern human genomes typically carry too few fixed differences to permit a robust estimate of contamination. For early modern humans, various groups have, therefore, relied on sites in the ancient sample that differ from a large dataset of present-day human mitochondrial sequences [13]. Additionally, a maximum-likelihood approach, which co-estimates sequencing error rates and contamination, has been applied to sequences originating from both early modern humans and archaic humans [14]. Deamination patterns have also been used to estimate contamination from present-day humans in mitochondrial DNA [10]. Software tools are available to measure overall deamination [15], identify the endogenous template [16], isolate deaminated fragments [9] and perform nuclear contamination estimates based on the X-chromosome [17]. However, there is currently no software for estimating mitochondrial contamination, which has been thoroughly tested to ascertain its accuracy, available for download for the aDNA research community.

We developed *schmutzi*, an iterative approach to assembling the endogenous mitochondrial genome while simultaneously estimating present-day human mitochondrial contamination in archaic and early modern human aDNA datasets. Our approach to determining the endogenous mitochondrial genome sequence relies on distinguishing the endogenous and the contaminant nucleotides, given a prior on: contamination, deamination frequency and length distribution of the fragments. Contamination is estimated using single nucleotide differences between the endogenous mtDNA sequence and a database of potential contaminant mitochondrial genomes. The consensus calling and contamination estimation are run iteratively until a stable contamination rate estimate is reached.

Schmutzi was tested on both simulated and empirical data. Our results show that schmutzi outperforms currently available methods in terms of accuracy of the endogenous call and contamination estimate, particularly at high levels of contamination. An open-source implementation of schmutzi in C++ has been released under the GPLv3.0 and is freely available together with the test datasets that were used [18]. On a desktop computer, schmutzi requires between 1 and 3 hours to reach convergence for approximately 1 million fragments aligned to the mitochondrial reference genome. Faster run times (∼30 minutes) can be achieved using multi-core systems.

## Results

**Fig. 2:**
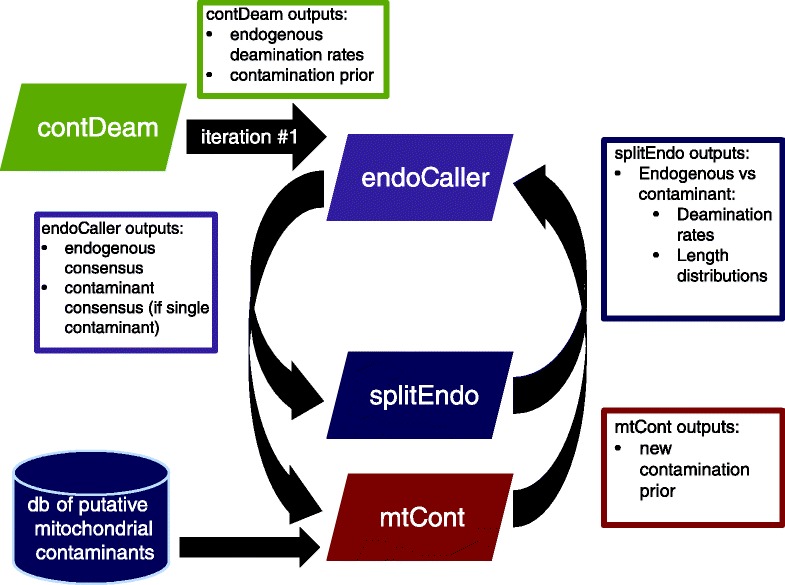
Schmutzi workflow. An initial contamination estimate is computed using the deamination rates of fragments by conditioning on the other end being deaminated and comparing these to the deamination rate of all fragments in the dataset (contDeam). This prior is provided to call an endogenous consensus (endoCaller). The consensus call is, in turn, used to re-estimate mitochondrial contamination (mtCont). Deamination rates and fragment length distributions are measured for fragments that support endogenous and contaminant mitochondrial genomes (splitEndo). The information from mtCont and splitEndo is used as input for re-calling the endogenous consensus (endoCaller). This cycle is repeated until a stable contamination rate is reached. *db* database

Schmutzi iteratively calls (i) the endogenous mitochondrial consensus sequence and (ii) a contamination estimate using two linked software programs (Fig. 2). The input for endoCaller, the consensus caller, is a set of aDNA sequences aligned to a mitochondrial genome reference, a contamination prior and deamination rates for the potentially endogenous and potentially contaminating DNA fragments. In the first iteration, the deamination rates and the prior for contamination are obtained using contDeam, a third sub-program of the schmutzi package (Fig. 2). contDeam implements a methodology described in previous studies [10], but incorporates some additional information including base quality and mapping quality into a Bayesian framework. An underlying assumption is that the base qualities are reasonably representative of the sequencing error probability. Recent versions of the default Illumina base caller, Bustard, provide such accuracy. The inputs for the contamination estimator, mtCont, are the same set of aligned fragments (in BAM format) that were used as input for contDeam, the endogenous consensus sequence determined by endoCaller, and a database of potential contaminant mitochondrial genomes. endoCaller, contDeam and mtCont can also be used as standalone applications. Each component program uses a Bayesian maximum a posteriori algorithm to estimate the most probable model parameters given the data. A list of the inputs and outputs for each of the three main programs, which are described in more detail in the sections below, is presented in Table 1.

**Table 1:** Inputs and outputs for the different programs described in ‘Methods’. Overview of the three main programs, contDeam, endoCaller and mtCont, with the helper program splitEndo for the iterative mode

Program	Input	Output
contDeam	BF	CRDP, EDR
endoCaller	BF, EDR, CP, DFL	EB
mtCont	BF, EDR, DB, EB	CRDB, CS
splitEndo	BF, EB	EDR, DFL

*BF* BAM file, *CRDP* present-day human contamination rate using deamination patterns, *CRDB* present-day human contamination rate using a database of putative contaminants and the endogenous base, *EDR* endogenous deamination rates, *CP* contamination prior, *DFL* distribution of endogenous/contaminant fragment lengths, *EB* endogenous base, *DB* database of putative mitochondrial contaminant genomes, *CS* most likely contamination source

We tested the performance of each of the component programs and of the iterative function, using both simulated and empirical data. For simulations, we used mitochondrial sequences from an early modern human, a Neanderthal and a Denisovan as the endogenous genomes and a present-day human as the contaminant genome. We also tested the performance on a number of previously published aDNA datasets [12, 19–21]. Further details about the test data can be found in ‘Methods’.

### Endogenous consensus calling

#### Simulated data

**Fig. 3:**
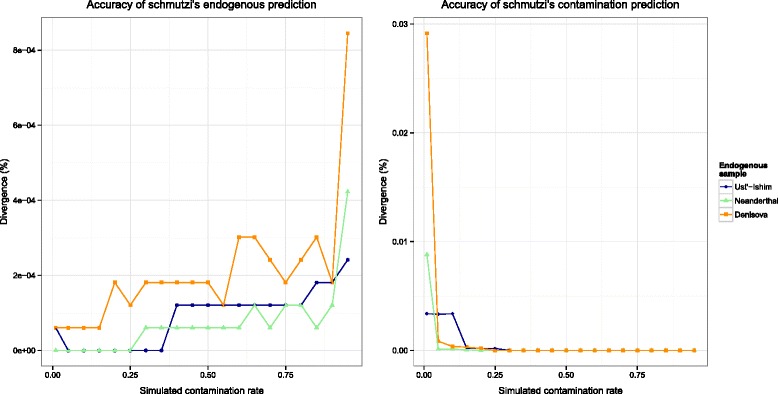
Effect of increasing contamination on endogenous genome sequence reconstruction and contaminant genome sequence reconstruction of simulated data. Accuracy of the ancient (**a**) and present-day contaminant (**b**) mitochondrial consensus sequences produced by schmutzi on simulated data for an early modern human, a Neanderthal and a Denisovan mitochondrial genome. We define an error as either a mismatch or an indel between the predicted endogenous sequence and the published mitochondrial sequence used for simulations. As contamination increases, inference of the endogenous mitochondrial genome becomes more difficult (**a**). In contrast, the prediction of the contaminant genome becomes more accurate at higher levels of present-day human contamination (**b**)

We ran schmutzi on simulated datasets created for three archaic genomes, each with increasing levels of present-day human contamination, and compared the endogenous and the contaminant genome sequences inferred by schmutzi to the published mitochondrial genome sequences for each individual (see Fig. 3). We also compared the endogenous consensus produced by (i) schmutzi, (ii) simply calling a consensus from all fragments using htslib and (iii) calling a consensus from fragments identified as deaminated by PMDtools. A Neanderthal was used as the endogenous mitochondrion and various levels of present-day human contamination were simulated. The mitochondrial sequences obtained using all three approaches to call the consensus were not considerably different (<1 %) at low levels of contamination (see Table 2). In contrast, at higher levels of contamination (>20 %), using only the deaminated reads reduces false calls by bases from present-day human contamination. However, schmutzi, which uses all the reads and includes additional sources of information such as fragment length and a contamination prior, prevents false calls that are due to the presence of present-day human contamination. For the remaining simulations, a Denisovan, Neanderthal or early modern human was used as the endogenous genome, with either single- or double-stranded deamination patterns. The accuracy of the consensus sequences generated by schmutzi and by other computational methods to the published reference sequences is presented in Additional file 1: Tables S9–S14.

**Table 2:** Similarity of the predicted endogenous mitochondrial genome sequence to the original Neanderthal reference sequence, at various rates of simulated contamination with present-day human DNA. An endogenous consensus call was performed using schmutzi on all fragments, and using PMDtools followed by htslib on the fragments labeled by PMDtools as endogenous. For comparison, we generated a simple consensus by running htslib on all sequenced fragments. While this approach works well at low amounts of contamination, it produces an incorrect consensus at higher levels of contamination when the presence of contaminating fragments is not accounted for using approaches like PMDtools and schmutzi. The number of indels are reported as either insertions or deletions in either the predicted consensus or the Neanderthal reference; hence, discrepancies in the final sum may occur

Contamination	Endogenous prediction			Endogenous prediction from			Mitochondrial consensus, called		
rate	from schmutzi			PMDtools and htslib			using htslib on all fragments		
	Matches	Mismatches	Indels	Matches	Mismatches	Indels	Matches	Mismatches	Indels
1 %	16,565	0	0	16,561	2	6	16,561	3	5
5 %	16,565	0	0	16,561	2	6	16,561	3	5
10 %	16,565	0	0	16,561	2	6	16,561	3	5
15 %	16,565	0	0	16,560	3	6	16,553	11	5
20 %	16,565	0	0	16,560	3	6	16,488	76	5
25 %	16,565	0	0	16,558	5	6	16,374	190	5
30 %	16,564	1	0	16,558	5	6	16,371	193	5
35 %	16,564	1	0	16,556	7	6	16,371	193	5
40 %	16,564	1	0	16,555	8	6	16,371	193	5
45 %	16,564	1	0	16,553	10	6	16,371	193	5
50 %	16,563	2	0	16,553	10	6	16,371	193	5
55 %	16,564	1	0	16,554	9	6	16,370	194	5
60 %	16,563	2	0	16,551	12	6	16,368	196	5
65 %	16,563	1	1	16,551	12	6	16,361	203	5
70 %	16,562	1	2	16,548	15	6	16,358	206	5
75 %	16,563	1	1	16,546	17	6	16,355	209	5
80 %	16,561	2	2	16,545	18	6	16,355	209	5
85 %	16,563	1	1	16,544	19	6	16,355	209	5
90 %	16,561	3	1	16,539	24	6	16,355	209	5
95 %	16,550	15	7	16,532	31	6	16,355	209	5

Schmutzi produced a consensus for both the endogenous and contaminant genomes that is very robust to high levels of contamination. Our results show that the endogenous consensus is accurately reconstructed for up to 50 % present-day human contamination for the double-stranded simulations and up to 70 % for the single-stranded ones. This is due to higher levels of deamination in the single-stranded simulations resulting in better ascertainment of the endogenous base.

We also called mitochondrial consensus sequences for each sample after processing the data using PMDtools (using the parameter -a to adjust quality scores and the recommended PMD score threshold of 3) to identify deaminated reads and then calling the consensus with htslib (default parameters and haploid model). The sequence similarity to the published ancient genomes was computed as for schmutzi. At higher levels of contamination, schmutzi is able to infer the endogenous genome more accurately than is possible using only htslib on the deaminated reads. It also performs better at higher levels of contamination than the approach of calling a consensus solely from deaminated reads using samtools mpileup (see Additional file 1: Results, Section 2.3.1). All three approaches provide a more accurate sequence than mitochondrial consensus genome obtained using MIA (see Additional file 1: Results, Section 2.3.1).

The improvement obtained by schmutzi over approaches that use only deaminated reads from highly contaminated samples results from the inclusion of length and observed ratio of endogenous and contaminant bases. Iteration increases the accuracy of the endogenous consensus call. We found that the initial call for the m dataset with a simulated contamination rate of 58 % had seven mismatches to its original reference while only a single mismatch remained after convergence.

At around 50 % present-day human contamination, the inference of the endogenous base becomes difficult as there is a near 50/50 distribution of endogenous and contaminant bases. As in the evaluation of the contamination estimate, to simulate low coverage, we subsampled the original BAM file with a simulated contamination rate of 48 %. This was done both for fragments with double-stranded and single-stranded associated damage. Our results show that, for this difficult target, we can infer the endogenous genomes to a coverage of about 20 × (see Additional file 1: Results, Section 2.3.2). This is also possible at 15 × but the endogenous calls need to be filtered for high-quality bases to avoid a high number of errors. This filtering also eliminates a significant portion (≈1/16) of the mitochondrial calls. Prediction of the endogenous mitochondrial genome at a lower coverage is possible if present-day human contamination is lower.

#### Empirical data

Because not all features of empirical aDNA datasets can be accurately simulated, we also tested schmutzi on the five empirical datasets described in Table 3. Only a subset of the original data was used here. The accuracy of the endogenous consensus sequences called using schmutzi was compared to the published mitochondrial genomes and to the consensus sequence called using htslib. For htslib, the quality scores of potentially deaminated bases were reduced to avoid incorrect calls at deaminated sites, like the procedure used in [12, 22].

**Table 3:** Empirical mitochondrial datasets. The numbers in parentheses represent the deamination rates when conditioning on the other end of the fragment being deaminated for heavily contaminated samples

Sample	mtDNA	Deamination		Present-day	Library ID
ID	coverage	rates (%)		contamination	and reference
	(×)	5^′^	3^′^		
Altai Neanderthal	1076	5.7	28.4	Low (∼1 %)	L9198 from [12]
Denisovan	258	14.8	33.9	Low (∼1 %)	B1108 from [20]
Ust’-ishim	124	2.7	3.4	Low (∼1 %)	B3899 from [19]
Mezmaiskaya Neanderthal B9687	711	8.8 (17.3)	13.3 (25.8)	High (∼40–50 %)	B9687 from [21]
Mezmaiskaya Neanderthal B9688	636	8.5 (15.0)	12.7 (24.1)	High (∼40–50 %)	B9688 from [21]

**Fig. 4:**
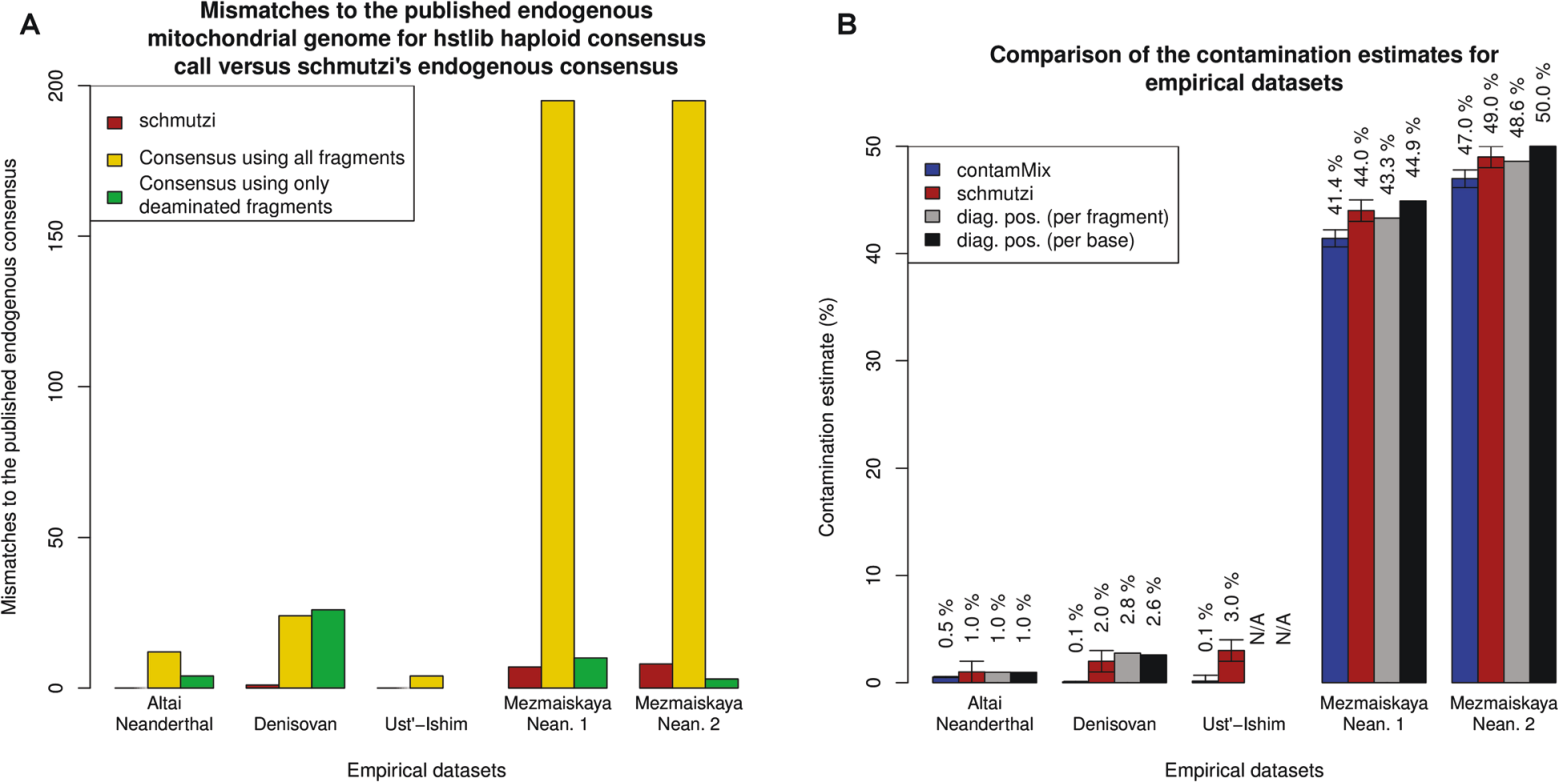
Consensus call and contamination estimate accuracy for empirical datasets. **a** The htslib consensus call (*yellow*) and the schmutzi consensus call (*red*) were performed on a subset of the data from three Neanderthals, one Denisovan and one early modern human. The number of mismatches between the mitochondrial consensus sequence and the published mitochondrial genome from the same individual was calculated. **b** Contamination was estimated using schmutzi (*red*) and contamMix v.1.0-10 (*blue*) and compared to the contamination computed using diagnostic positions (*gray* per fragment and *black* per base). For the two Mezmaiskaya individuals, the endogenous genome used for comparison was obtained using another library with low levels of contamination from the same individual. *diag pos* diagnostic position, *Nean* Neanderthal

At contamination rates less than 5 %, the consensus sequences called with htslib were highly similar (between one and five mismatches) to the published mitochondrial genome sequences (see Fig. 4). However, at higher contamination rates (>40 %), the consensus sequence becomes increasingly inaccurate when called with htslib. In contrast, the consensus sequence produced by schmutzi is robust to higher contamination (40–50 %). For the highly contaminated Mezmaiskaya samples, we assessed the effect of using only deaminated fragments to generate the consensus using htslib. This approach has been used previously and substantially reduces the amount of contamination. Indeed, we show that the consensus obtained using htslib and only deaminated fragments improves the accuracy of the consensus sequence (see Fig. 4) but that the consensus sequence produced by schmutzi is still more accurate in all but one case, which was influenced by capture bias (see paragraphs below and ‘Discussion’).

**Fig. 5:**
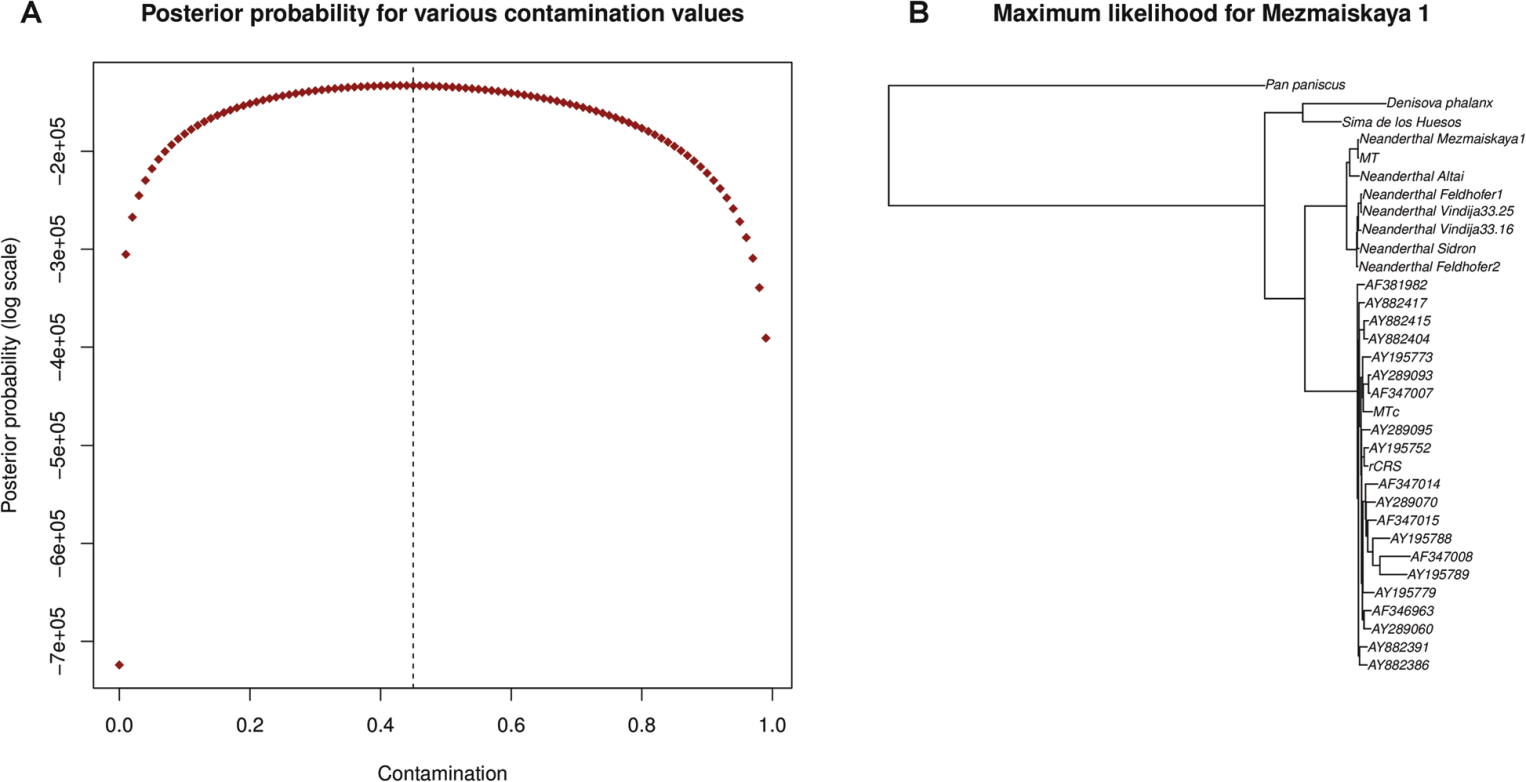
Contamination estimates and phylogenetic placement of Mezmaiskaya 1 (library ID B9687). **a** The posterior probability distribution for contamination in Mezmaiskaya 1. The *dotted line* represents the estimate obtained using an ad hoc method based on fixed sites. **b** A maximum-likelihood tree showing the placement of the mitochondrial genome of Mezmaiskaya 1 (labeled *MT* in the tree) and the inferred contaminant (labeled *MTc* in the tree), compared to 20 present-day humans and nine archaic humans

To evaluate further the accuracy of the endogenous consensus calling, a maximum-likelihood phylogenetic tree was computed using the high-quality bases (≥200 PHRED scale) for both the inferred endogenous and contaminant genomes (see Fig. 5b), and another using the unfiltered positions (see Additional file 1: Results, Section 2.2.3). The tree for the high-quality bases has a higher likelihood than the unfiltered one. As expected, our endogenous mitochondrial genome falls within the Neanderthal lineage, more precisely on the Mezmaiskaya branch, whereas the contaminant one falls within the range of human variation. Our algorithm is, therefore, able, without any prior phylogenetic information, to separate the endogenous sequences from the contaminant portions of the alignment.

We examined in more detail the sequence inferred for the mitochondrial genome of the Neanderthal from Mezmaiskaya 1 (library ID B9687), which was generated from the same individual for which a high-quality mitochondrial genome from a library with low contamination is available (GenBank FM865411). We note that the contaminating mitochondrial sequence is not known.

Under the assumption that the sequence from GenBank is without errors, the endogenous genome inferred by schmutzi should match perfectly this reference sequence. The inferred endogenous sequence differed by nine of the 16,608 bases. We noted that this region falls in the D-loop, which is typically quite divergent. We speculated that the incorrect identification of these nine bases may arise from an ascertainment bias due to the mitochondrial capture of the Mezmaiskaya sample using probes based on the human mitochondrial sequence. Indeed, we found that in this region the endogenous bases were significantly underrepresented compared to the contaminant (75 % rather than the average of 50 % for the whole mitochondrial genome). However, these bases tend to have low consensus base quality, which implies that the consensus calls at these positions is unreliable. Filtering for consensus base quality ≥200 (PHRED scale) reduces the number from nine mismatches to one. This single mismatch is in the poly-C region (position 16,184), which is routinely removed in downstream analyses [23, 24].

### Accuracy of contamination estimates

#### Empirical data

We estimated contamination for each of the five empirical datasets using schmutzi and contamMix (v1.0-10), an implementation from the authors of a previously described maximum-likelihood method for estimating mitochondrial contamination [14, 19].

The correct contamination estimate was taken to be that obtained from fragments aligned to sites in the reference mitochondrial genome where Neanderthals or Denisovans differ from 20 present-day humans (diagnostic sites). Since there are too few diagnostic sites, this approach could not be used for the early modern human data.

For the Altai Neanderthal and Denisovan samples, which have low contamination, both schmutzi and contamMix accurately estimate the contamination (see Fig. 4). However, for the highly contaminated Mezmaiskaya Neanderthal samples, schmutzi’s contamination estimates are closer to the estimates provided using diagnostic positions (44.1±0.8 and 49.3±0.7 for Mezmaiskaya samples 1 and 2, respectively). For Mezmaiskaya 1, for instance, using the 111 diagnostic sites, there were 2,443,418 individual bases supporting the Neanderthal base and 1,989,785 supporting the present-day human base, resulting in an estimated contamination of 44.9 % (per nucleotide basis). The contamination estimates obtained using diagnostic positions are constant even when filtering for high base quality and removing potentially deaminated bases. In comparison, the contamination estimate from schmutzi was 44±1 % and the estimate from contamMix was 41.4±0.8 %. We speculate that this is due to schmutzi’s iterative inference of the contaminating genome (see ‘Simulated data’). To explore the results, we plotted the distribution of the posterior probability for the contamination estimate from one of the individuals (library ID B9687); see Fig. 5a. The posterior probability peaks at the one obtained using diagnostic positions.

To test further the ability of schmutzi to estimate contamination and infer the endogenous sequence, we downloaded 22 different aDNA datasets from four different studies from different research groups. To compare our estimates to those produced by existing methods, we also ran contamMix on the same samples. Our results show that schmutzi is more accurate and our implementation faster than the existing methodologies (see Additional file 1: Results, Section 2.2.5).

#### Simulated data

**Fig. 6:**
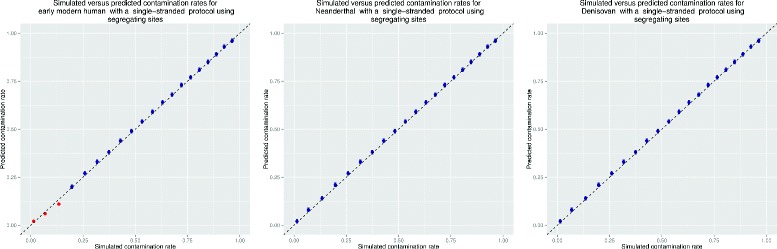
Simulated versus measured contamination rates. Several sets contained simulated aDNA fragments from a mitochondrial genome belonging to an early modern human (left), a Neanderthal (middle) or a Denisovan (right). All simulated sets had damage patterns associated with a single-stranded library protocol. The double-stranded figure can be found in Additional file 1: Results. A contaminating present-day human was pooled together at various rates to simulate contamination. The *dotted black line* represents a perfect prediction, and *blue dots* are the predicted rates of contamination by schmutzi once convergence was achieved. The *red dots* represent sets for which the algorithm stopped prematurely due to lack of information about the contaminant fragments. The *black whiskers* represent the 95 % confidence interval for contamination

To evaluate the range of contamination and coverage over which schmutzi can be used, we used the three simulated datasets with increasing levels of contamination and at varying coverage. For the simulated datasets, the contamination rates predicted by schmutzi correlate well with those simulated (Fig. 6 and Additional file 1: Fig. S16).

To test the accuracy of our algorithm to existing methods, we ran our algorithm and contamMix on a simulated dataset of 1 million fragments with double-stranded deamination patterns. The endogenous mitochondrial genome used was an early modern human with 50 % present-day human contamination. Our result show that the schmutzi algorithm offers superior accuracy compared to this existing method for estimating early modern human contamination (see Table 4). Results for the maximum-likelihood methods used by contamMix for the remaining samples are presented in Additional file 1: Results, Section 2.3.6. We also evaluated the impact of having multiple contaminant mitochondrial genomes (see Additional file 1: Results, Section 2.3.7) where an underestimate is observed for the early modern human at very high levels of contamination (70 %) and at a high mixture (e.g., 50/50) of different contaminant mitochondrial sequences.

**Table 4:** Accuracy of contamination estimates on a simulated early modern human with double-stranded deamination patterns and high present-day modern human contamination. Three cores were used for every program. The programs contamMix and contDeam estimate contamination on a per fragment basis while mtCont estimates contamination on a per nucleotide basis. The contamination on a per nucleotide basis is higher due to the longer average length of contaminating fragments

Contamination	Contamination	Run time
estimate	estimate	
method		
Target contamination rate: 50 % (fragment basis)		
contamMix 1.0-10	54.9±0.7 %	4 days
Schmutzi (contDeam)	49.0±0.5 %	68 s
Target contamination rate: 58.2 % (nucleotide basis)		
Schmutzi (mtCont without the predicted contaminant)	32.0±1.0 %	183 m
Schmutzi (mtCont with the predicted contaminant)	60.0±1.0 %	200 m

The predictions of our algorithm for the remaining simulated datasets are presented in Additional file 1: Results, Section 2.3.5. The predicted present-day human contamination rates matched the simulated contamination rates.

To evaluate the effect of coverage on schmutzi’s contamination estimate, we analyzed a dataset with 47 % contamination and subsampled this to various levels of coverage. We chose 47 % as a level of contamination that makes the use of currently available tools difficult. Furthermore, at this level of contamination, there is an almost even number of endogenous and contaminant bases thus making the inference of each one relatively difficult for our model.

**Fig. 7:**
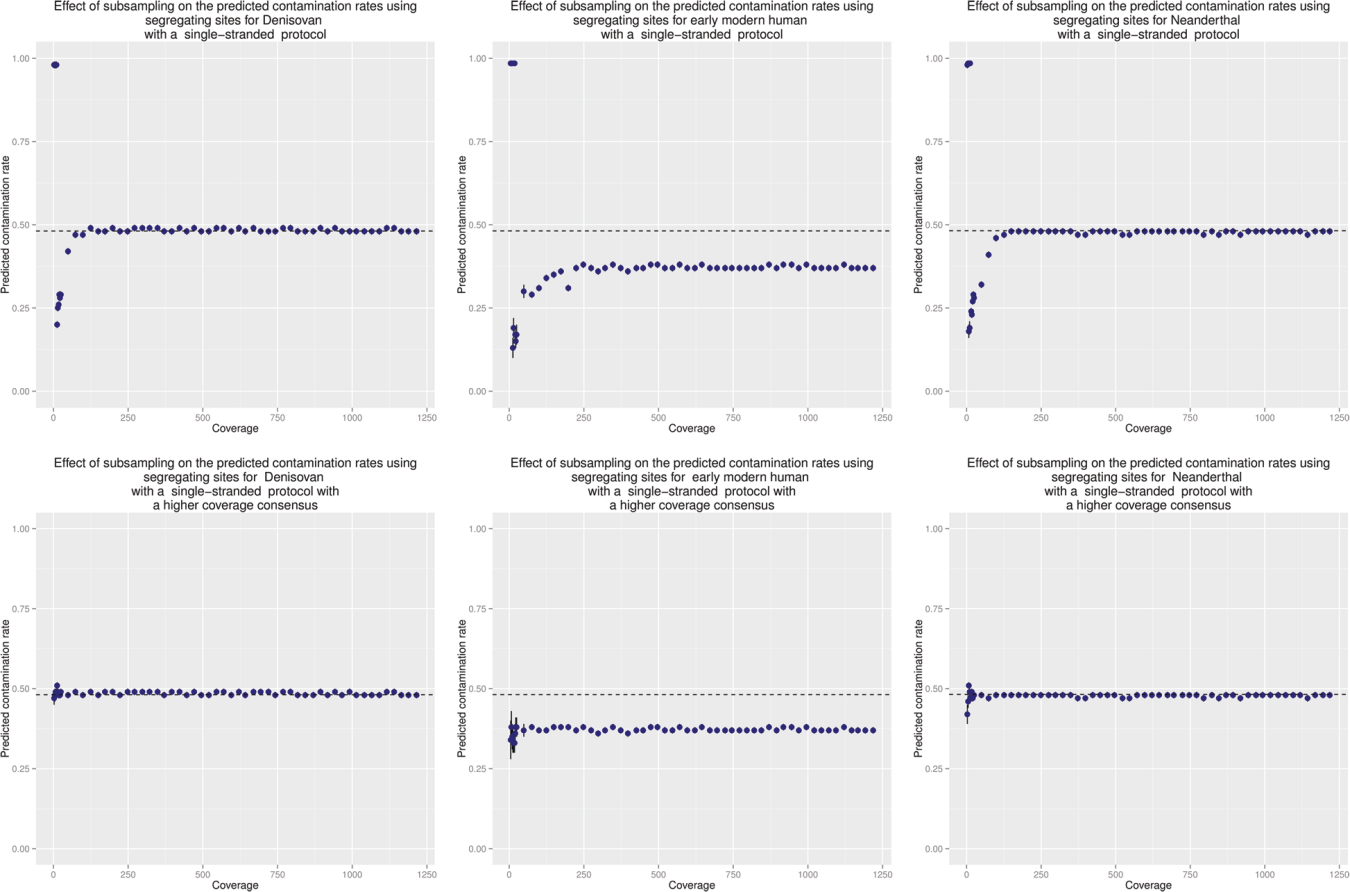
Robustness of the contamination estimate to lower coverage. The simulated dataset with a contamination rate of ∼47 % and single-stranded deamination patterns was subsampled at various coverages from 0 to 1250 ×. Top: Contamination rates were estimated across a range of coverages in simulated data for a Neanderthal, a Denisovan and an early modern human (Ust’-Ishim). Bottom: Contamination estimates when a high-quality mtDNA sequence from a closely related individual is used as the endogenous genome. Robust estimates can be made down to 5 × coverage even at 47 % contamination. For the early modern human, the contamination estimate provided was computed using the database alone and not the prediction of the contaminant genome thus leading to underestimates (see Table 4 for an example of the effect of using the predicted contaminant in the contamination estimate)

For the simulated Neanderthal, the contamination estimated by schmutzi is stable down to a coverage of ∼ 100× (see results for the single-stranded protocol in the top row of Fig. 7 and Additional file 1: Fig. S18 for the double-stranded data). At coverage less than 100 × for the single-stranded data and contamination simulated here, estimation of contamination becomes difficult. For the double-stranded data, due to lower rates of deamination, our estimates are stable at coverages down to 150 ×. However, there are cases where an accurate mitochondrial genome sequence from another closely related individual can be used as a proxy to compute contamination rates (see Additional file 1: Fig. S19). Using this heuristic we can obtain accurate contamination rates for coverage down to ∼ 5× (see Fig. 7 bottom row).

We also note that the contamination prior estimates based only on deamination patterns also show a high correlation to those simulated (see Additional file 1: Results, Section 2.3.3). However, these estimates do not have the same robustness to low coverage as the final contamination estimate produced by the iterative approach. Additionally, we show that using a sensitive aligner like SHRIMP [25] does not cause drops in coverage around regions of high divergence of the Denisovan mitochondrial genome to the human mtDNA, thus allowing for a more reliable contamination estimate (see Additional file 1: Results, Section 2.1).

### Contaminant consensus calling

The accuracy of the contaminant genome inferred from the simulated datasets increased as the amount of contamination increased (Fig. 3). However, at less than 1 % contamination, schmutzi cannot accurately infer the contaminant genome. An analysis of the inferred contaminant sequences is presented in Additional file 1: Tables S6 and S7.

For our empirical Mezmaiskaya samples, while the endogenous genome sequence is known, the nature of the contaminant is not. We can, however, take the inferred contamination genome and verify whether it falls within a known mitochondrial haplogroup. Using HaploGrep [26, 27], we determined that our inferred mitochondrial genome pertained to the T2b3 haplogroup with a confidence of 93.1 %. Out of a total of 33 diagnostic sites for this haplogroup, only one site, with a relatively low consensus prediction quality from our software (65 on a PHRED scale), was not the expected diagnostic base.

## Methods

### Test data

We tested the performance of schmutzi on simulated and empirical mitochondrial sequence data from both archaic humans and early modern humans. Simulated mtDNA datasets with increasing levels of contamination were created by fragmenting and deaminating the mitochondrial genome sequences of a Denisovan (GenBank: FN673705.1) [20], a Neanderthal (GenBank: AM948965.1) [12] and an early modern human (Ust’-Ishim individual [19]) and adding increasing amounts of contamination from a single, randomly selected present-day human mitochondrial genome (GenBank: KJ446110.1). We used empirical deamination rates from data prepared using a double-stranded library preparation protocol (C → T at the 5^′^ end and G → A at the 3^′^ end, rates from [13]). We repeated the simulations by adding deamination rates from empirical data prepared using a single-stranded library protocol (C → T at both ends, rates from [22], see Additional file 1: Methods).

The empirical data included Illumina sequences from the same three ancient individuals as well as sequence data for two additional Neanderthal individuals from Mezmaiskaya [21] (NCBI SRA ID: PRJEB6014), which were selected because of the high rate of present-day human contamination present in the sequencing libraries [12].

We compared the accuracy of the consensus sequence called by schmutzi to the consensus sequences generated using a set of typical approaches that have been described in the literature: (i) MIA [7], (ii) PMDtools to identify deaminated reads followed by a haploid consensus call using htslib [9] and (iii) samtools mpileup (obtained from [28]) after removing deaminated reads [10]. We also compared schmutzi’s contamination estimates to the known contamination in the simulated sequence data, to the estimates based on diagnostic sites for the empirical data, and to the estimates obtained from the maximum-likelihood approach described in [14, 19]. This is currently the only published method that can estimate mitochondrial contamination for both early modern humans and archaic humans. To assess the robustness of schmutzi to varying coverage, fragments were downsampled from 1 % to 50 % of the data using a uniform probability distribution.

We first discuss how a reasonable contamination prior can be obtained using deamination patterns. We then provide details of the algorithm behind the endogenous consensus caller and show how the contamination is estimated using the output of the endogenous consensus caller. A list of the symbols used throughout this section is found in Table 5.

**Table 5:** Notation used in ‘Methods’

Symbol	Definition
$\mathbb {R}$	Set of all fragments
$\mathbb {E}$	Set of all fragments from the endogenous genome
*R*_*j*_	a particular fragment in *R*, with *l* bases $\{ r_{1}, \dots, r_{l} \}$ and respective error probabilities $\{ \epsilon _{1}, \dots, \epsilon _{l} \}$, which are given by the per-base quality scores
*E*	The event that a sequencing error has occurred
*D*	The event that deamination has occurred
*C*	The event that *R*_*j*_ was sampled from a contaminant mitochondrial genome
*M*	The event that *R*_*j*_ was correctly mapped
$m_{R_{j}}$	Probability that *R*_*J*_ is mismapped (*P*[¬*M*])
*b*_e_	The base from the endogenous genome
*b*_c_	The base from the contaminant genome
*c*	The base from the contaminant genome used by mtCont, obtained from a database
*r*_*i*_	The base at position *i* from fragment *R*_*j*_
*ε*_*i*_	The probability that base *r*_*i*_ has a sequencing error as determined by the base caller
¬	Denotes the complement of an event (event has not occurred)
*c*_d_	Contamination rate, estimated by contDeam
*c*_r_	Contamination rate, estimated by mtCont
*c*_c_	Prior on contamination rate provided as input to endoCaller
endo_dist_	log-normal distribution of the fragment length for the endogenous fragments
cont_dist_	log-normal distribution of the fragment length for the contaminant fragments

### contDeam: determining a contamination prior using deamination patterns

The first iteration of the endogenous genome inference needs a contamination prior that is ideally a reasonable approximation of the actual contamination rate. This first contamination estimate is computed by contDeam (see schematic in Fig. 2). This program computes the likelihood of observing the aDNA fragments aligned to the reference genome given fixed endogenous deamination patterns and a prior on the rate of present-day human contamination. It then returns the contamination rate with the highest posterior probability. This contamination rate is the most likely value needed to explain the difference between deamination rates for fragments identified as endogenous and overall deamination rates for all the fragments of the entire dataset. We start from the assumption that only the endogenous DNA has the deamination patterns typical of aDNA and that contaminant fragments are not deaminated and will, therefore, only reduce overall deamination rates. Previous studies suggest that deamination is rare in contaminants younger than about 100 years old [8]. Having deaminated contaminant fragments may lead to underestimates. We discuss the extent of the potential underestimate at the end of this section.

To identify the endogenous fragments and derive their deamination rate, there are two possible approaches. The first involves the separation of the endogenous and contaminant fragments using diagnostic positions on the mitochondrial genome. This is relatively straightforward when dealing with Neanderthal or Denisovan individuals, as their mitochondrial genome sequences fall outside of present-day human variation [29, 30]. For instance, there are 111 diagnostic positions on the mitochondrial genome sequence at which seven Neanderthals share the same base, which differs from 20 present-day humans.

However, when the endogenous sample is an early modern human and falls within present-day human variation, this approach lacks power due to the rarity of such diagnostic sites. A second strategy takes advantage of the observation that deamination at the 5^′^ end of the fragment is independent of the deamination occurring at the 3^′^ end and vice versa. By conditioning on observing deamination at one end and measuring the rates of deamination at the other, an estimate of the deamination rates of the endogenous fragments can be obtained [10]. This second strategy requires an endogenous base to measure rates of deamination. We, therefore, use the mitochondrial reference sequence as the endogenous template. This assumption yields accurate results even for the highly divergent Denisovan mitochondrial genome. The contamination prior estimated by schmutzi uses this second approach by default. The estimate of the endogenous deamination rate is calculated only once, when launching contDeam. The contamination estimate obtained by contDeam is subsequently used as contamination prior for the first iteration (see Fig. 2).

Let $\mathbb {R}$ be the set of all fragments and $R_{j} \in \mathbb {R}$ be a particular aligned fragment of length *l*. We compute the probability of observing this particular alignment to the reference genome given two models: (i) the null model, where any difference from the reference can be solely explained by sequencing error or (ii) the deaminated model, where deamination and sequencing errors could have given rise to this particular alignment to the reference. For fragment *R*_*j*_, let $\{ r_{1}, \dots, r_{l} \}$ be the individuals nucleotides and their respective error probabilities $\{ \epsilon _{1}, \dots, \epsilon _{l} \}$, both of which are provided by the base caller. Let *E* denote the event that a sequencing error has occurred, *D* the event that deamination has occurred and let ¬ denote the complement of an event (i.e., the event has not occurred).

We compute the likelihood of observing the base *r*_*i*_∈*R*_*j*_, aligned to the reference nucleotide *n*, by assuming that nucleotide *n* was the endogenous template. The likelihood of observing *r*_*i*_ under the null model, denoted *p*_*n*_(*r*_*i*_), is computed by taking into account two events, either a sequencing error has occurred or it has not:

(1) $$ P_{n} [\!r_{i}] = \begin{cases} (1-\epsilon_{i})\,P[\!n \to r_{i} |\neg E], & \text{if}\,n = r_{i} \\ \epsilon_{i}\,P[\!n \to r_{i} |E ], & \text{if}\,n \neq r_{i} \\ \end{cases}   $$

where *p*[*n*→*r*_*i*_|¬*E*] is the probability that *r*_*i*_ is observed if *n* was the template. This quantity is 1 as both nucleotides are identical. The other term, *p*[*n*→*r*_*i*_|*E*], is the probability of a substitution from nucleotide *n* to *r*_*i*_ given sequencing error. This term is approximately equal to 1/3 but empirical substitution rates are used (see next section for details). Under the deaminated model, the probability of seeing base *r*_*i*_ (given the template *n*) denoted *p*_*d*_(*r*_*i*_) is

(2) $$ {}P_{\mathrm{d}} [\!r_{i}] =\left\{ \begin{array}{l} (1-\epsilon_{i})\,P[\!n \to r_{i} |\neg D \cap \neg E ], \text{if}\,n = r_{i} \\ (1-\epsilon_{i})\,P[\!n \to r_{i} |D ]\\ +\\ \epsilon_{i}\,P[\!n \to r_{i} |E], \quad\quad \quad\quad\quad\quad \text{if}\,n \neq r_{i} \\ \end{array} \right.  $$

as three events need to be taken into account: (i) $\neg D \cap \neg E$, absence of both sequencing error and deamination (if *n*=*r*_*i*_), and either (ii) *D* deamination or (iii) *E* error occurred and *n*≠*r*_*i*_. We currently ignore the probability of observing the data given that both deamination and a sequencing error have occurred ($D \cap E$) as it is very unlikely compared to the scenarios mentioned above. The probability of observing a substitution *n*→*r*_*i*_ given deamination (*P*[ *n*→*r*_*i*_|*D*]) is computed using the endogenous deamination rates that were described earlier. The term $P [\!n \to r_{i} |\neg D \cap \neg E]$ is the probability that base *r*_*i*_ remains as is. This probability is obtained by subtracting from 1, the deamination probability of the remaining bases. For instance, if a given base has a deamination rate of 0.3, the probability that the base remains as is, given the absence of sequencing error, is 0.7.

Let *C* be the event that we sampled the fragment *R*_*j*_ from a contaminant mitochondrial genome and ¬*C* be the event that we sampled from the endogenous genome. We compute the probability of observing fragment *R*_*j*_ with its alignment to the reference given that it was sampled from the endogenous genome by assuming that each base is an independent observation and that the probability of seeing any difference to the reference is explained by the deaminated model described by Eq. 2. Hence, we have:

(3) $$ P[\!R_{j} | \neg C ] = \prod_{r_{i} \in R_{j}} P_{\mathrm{d}} [\!r_{i}]   $$

and similarly, if *R*_*j*_ was sampled from the contaminant, the probability of any base that differs from the reference is explained solely by sequencing errors (as defined by Eq. 1). This probability for all bases is given by

(4) $$ P[\!R_{j} | C ] = \prod_{r_{i} \in R_{j}} P_{n} [\!r_{i}].   $$

There are two events that could have occurred: either we sampled the fragment from the contaminant with probability denoted *c*_d_ or we sampled from the endogenous genome with probability 1−*c*_d_. The goal of contDeam is to estimate *c*_d_ given the data. Using Eqs. 3 and 4, we obtain the probability of observing *R*_*j*_ given that it is sampled from the contaminant at rate *c*_d_:

(5) $$ P [\!R_{j}| c_{\mathrm{d}} ] = P[\!R_{j}|\neg C] (1-c_{\mathrm{d}}) + P [\!R_{j}|C ] c_{\mathrm{d}}   $$

since *P*[ *C*]=*c*_d_ by definition. The probability of observing all the fragments in set *R*, assuming the reference as the template and the endogenous deamination rates that were initially computed, for a given contamination rate *c*_d_, is given by assuming that each fragment is an independent observation:

(6) $$ P [\!\mathbb{R} | c_{\mathrm{d}} ] = \prod_{R_{i} \in \mathbb{R}} P [\!R_{i} | c_{\mathrm{d}}].   $$

Finally, the posterior probability of the contamination rate is given by omitting the probability term for the data ($P[\mathbb {R}]$) as it is independent of the contamination rate, and using a uniform prior for the contamination rate (*P*[ *c*_d_]). This posterior probability is, therefore,

(7) $$ P [\!c_{\mathrm{d}}|\mathbb{R}] \propto P_{d} [ \!\mathbb{R} |c_{\mathrm{d}}].   $$

We then produce the contamination rate $\widehat {c_{\mathrm {d}}}$ with the highest posterior probability:

(8) $$ \widehat{c_{\mathrm{d}}} = \text{argmax}~P [\!c_{\mathrm{d}} | \mathbb{R} ].  $$

One advantage of this approach is that it does not require the computation of the endogenous consensus. However, it also does not allow the user to identify the source of the contamination. Furthermore, it may underestimate contamination if the contaminant is deaminated (see Additional file 1: Results, Section 2.3.4). The assumption that the mitochondrial genome reference sequence is the template does not seem to influence the final contamination estimate even for the highly divergent Denisovan mitochondrial genome (see Additional file 1: Results, Section 2.3.3).

### endoCaller: mitochondrial consensus call

The first step of the iterative process is to call an initial consensus of the endogenous mitochondrial genome from mtDNA fragments aligned to a mitochondrial reference sequence (endoCaller in Fig. 2).

The consensus call relies on computing the probability of observing the aligned aDNA data for a particular pair of endogenous and contaminant nucleotides at a specific site, given a fixed contamination prior and fixed deamination patterns. The endogenous consensus caller seeks to identify the pair of endogenous and contamination nucleotides with the highest posterior probability given the aligned aDNA fragments. We also consider insertion/deletion at a given position. We assume that at any position there is a single nucleotide from the present-day human contaminant. The impact of having multiple contaminating nucleotides was also considered (see ‘Results’).

For a given position in the mitochondrial reference sequence, assuming a single contaminant, there are two bases to infer, *b*_e_ and *b*_c_, for the endogenous and contaminant genomes, respectively. Let *R* be the set of all fragments and $R_{j} \in \mathbb {R}$ be a fragment of length *l* that overlaps the position. Let $\{ r_{1}, \dots, r_{l} \}$ be the individual nucleotides of the fragment *R*_*j*_, as identified by the base caller. The respective error probabilities $\{ \epsilon _{1}, \dots, \epsilon _{l} \}$ for each base are also provided by the base caller.

For the position to be evaluated, let the nucleotide *r*_*i*_ be the base of fragment *R*_*j*_ that aligns at that specific position. Let *ε*_*i*_ be its error probability as determined by the base caller. Let *M* be the event that *R*_*j*_ was correctly mapped and *P*[*M*] is estimated using the mapping quality provided by the mapper. Let $\mathbb {E}$ be the set of fragments from the endogenous mitochondrial genome such that $\mathbb {E} \subseteq \mathbb {R}$. We will assume that the a priori probability that fragment *R*_*j*_ is endogenous is $P[\!R_{j} \in \mathbb {E}]$. This quantity is computed using both the deamination patterns of the fragment and its length to derive a probability of that fragment being endogenous. The equations for this expression are described in greater detail at the end of this section.

In having observed the base *r*_*i*_, there are two possibilities: the base came either from the contaminant with probability $1-P[\!R_{j} \in \mathbb {E}]$ or from the endogenous sample with probability $P[\!R_{j} \in \mathbb {E}]$. We assume for now that the fragment was properly mapped (i.e., *M* occurred). The final equation, which considers either possibility, is presented in ‘Mapping’. The probability of observing base *r*_*i*_, denoted by *P*[ *r*_*i*_|*b*_e_,*b*_c_,*M*], is given by

(9) $$ P[\!R_{j} \in \mathbb{E}] P_{\mathrm{c}} [\!r_{i}|b_{\mathrm{e}},M] +\ (1- P[\!R_{j} \in \mathbb{E}]) P_{\mathrm{c}} [\!r_{i}|b_{\mathrm{c}},M].   $$

The expression *P*_e_[*r*_*i*_|*b*_e_,*M*] is the probability of observing *r*_*i*_ given that the fragment is endogenous and *b*_e_ is the endogenous base. Let *E* denote the event that a sequencing error has occurred and let ¬*E* denote the complement of the event or, in other words, that the sequencing was correct and no error has occurred. The quantity *P*_e_[ *r*_*i*_|*b*_e_,*M*] is given by

(10) $$ (1-\epsilon_{i}) P_{\mathrm{e}} [\!b_{\mathrm{e}} \to r_{i}|\neg E,M] +\ \epsilon_{i} P_{\mathrm{e}} [\!b_{\mathrm{e}} \to r_{i} | E, M ].   $$

Given that the base is correct (i.e., without sequencing error), both *r*_*i*_ and *b*_e_ should be identical; hence,

(11) $$ P_{\mathrm{e}} [\!b_{\mathrm{e}} \to r_{i} | \neg E, M] = \begin{cases} 1, & \text{if}\,b_{\mathrm{e}} = r_{i} \\ 0, & \text{if}\,b_{\mathrm{e}} \ne r_{i}. \\ \end{cases}   $$

However, due to deamination, it is possible to have a substitution with the probability derived from the deamination profile entered as input. Let *Ω* be the set of all DNA bases (*Ω*={*A*,*C*,*G*,*T*}). Under the deamination model, the term *P*_e_[ *b*_e_→*r*_*i*_|¬*E*,*M*] becomes

(12) $$ \begin{cases} 1 - \sum\limits_{b_{\mathrm{e}}' \in \Omega \setminus b} \text{rate}_{\text{deam}}\left(b_{\mathrm{e}} \to b_{\mathrm{e}}'\right), & \text{if}\,b_{\mathrm{e}} = r_{i} \\ \text{rate}_{\text{deam}}(b_{\mathrm{e}} \to r_{i}), & \text{if}\,b_{\mathrm{e}} \ne r_{i} \\ \end{cases}   $$

where rate_deam_(*b*→*r*_*i*_) is the rate of nucleotide substitution from *b* to *r*_*i*_ due to deamination at that specific position of the fragment. As stated before, the deamination rates per base for each position of the fragment are entered as input and remain unchanged by endoCaller. For sequencing errors, the probability of base substitution can be obtained using the assumption that any given nucleotide is equally likely to be miscalled as any of the remaining three nucleotides:

(13) $$ P_{\mathrm{e}} [\!b_{\mathrm{e}} \to r_{i} |E,M] = \frac {1} {3}, \quad \forall b_{\mathrm{e}} \ne r_{i}.   $$

However, studies on Illumina sequencing errors show that this assumption is often incorrect [31]. We, therefore, recommend using empirical nucleotide substitutions rates from an Illumina sequencing run (provided with the software package). The new error probability term becomes

(14) $$ P_{\mathrm{e}} [\!b_{\mathrm{e}} \to r_{i} |E,M] = \frac { \# b_{\mathrm{e}} \to r_{i}} { \sum\limits_{b_{\mathrm{e}}' \in \Omega \setminus b_{\mathrm{e}}} \# b_{\mathrm{e}} \to b_{\mathrm{e}}' }  $$

where *#x*→*y* represents the number of times a mismatch between the reference base *x* to an observed *y* occurred. These counts were determined using spiked-in control sequences aligned to the PhiX genome provided by Illumina Corp.

A similar computation is derived for the probability of seeing *r*_*i*_ given that we sampled the contaminant base *b*_c_ (*P*_*c*_[ *r*_*i*_|*b*_c_,*M*]). However, the deamination profile provided as input for the contaminant fragments are different from the endogenous ones and tend to be much lower (the end of ‘Methods’ describes the test data for empirical deamination rates for both endogenous and contaminant fragments). Our mitochondrial consensus caller endoCaller allows for deamination of the contaminant unlike contDeam, which assumes that the contaminant fragments have little to no deamination.

#### Mapping

Thus far, it was assumed that the fragment *R*_*j*_ was correctly mapped. For fragments not properly mapped, we estimate that the probability of seeing the base *r*_*i*_ is independent of bases *b*_e_ and *b*_c_ and is simply the probability of observing *r*_*i*_:

(15) $$ P[\!r_{i}|b,\neg M] = P[\!r_{i}] = \frac{1} {4}.   $$

The probability of fragment *R*_*j*_ being incorrectly mapped is obtained using its mapping quality, and we, therefore, combine Eqs. 9 and 15 into one to compute the final probability of observing the base *r*_*i*_, denoted by *P*[ *r*_*i*_|*b*_e_,*b*_c_]:

(16) $$ \left(1-m_{R_{j}}\right) P[\!r_{i}|b_{\mathrm{e}},b_{\mathrm{c}},M] +\ m_{R_{j}} P[\!r_{i}|b_{\mathrm{e}},b_{\mathrm{c}},\neg M]   $$

where $m_{R_{j}}$ is the probability that the fragment *R*_*j*_ is mismapped (i.e., $m_{R_{j}} = P[\neg M] $).

#### Producing the most likely bases

The probability of observing the data given every endogenous and contaminant base has been described. However, the posterior probability of the pair of bases given the data $\mathbb {R}$ is the quantity that is sought. We assume that every fragment *R*_*j*_ represents an independent observation and we also consider that the likelihood of bases *b*_e_ and *b*_c_ given the data is proportional to the probability of observing the data given the pair of nucleotides times a flat prior:

(17) $$ P[\!b_{\mathrm{e}},b_{\mathrm{c}}|\mathbb{R}] \propto \prod_{R_{j} \in \mathbb{R}} P[\!R_{j}|b_{\mathrm{e}},b_{\mathrm{c}}] \frac {1} {4^{2}}.  $$

Once the joint probability for all pairs of nucleotides is computed, a marginalization over *b*_c_ is used to obtain the likelihood of a given endogenous base:

(18) $$ P[\!b_{\mathrm{e}}|\mathbb{R}] = \sum_{b_{\mathrm{c}} \in \Omega} P[\!b_{\mathrm{e}},b_{\mathrm{c}}|\mathbb{R}].  $$

A marginalization over the endogenous base is used to call the contaminant base. Finally, the most likely endogenous nucleotide $\widehat {b_{\mathrm {e}}}$ is produced:

(19) $$ \widehat{b_{\mathrm{e}}} = \underset{b_{\mathrm{e}} \in \Omega}{argmax}\,P [\!b_{\mathrm{e}}|\mathbb{R}].  $$

The probability of error on $\widehat {b_{\mathrm {e}}}$ is given by the ratio of the sum of the probabilities for all alternative bases except the most likely over the sum of the probabilities for all bases:

(20) $$ P[ \neg \widehat{b_{\mathrm{e}}}|\mathbb{R}] = \frac { \sum\limits_{b_{\mathrm{e}} \in \Omega \setminus \widehat{b_{\mathrm{e}}}} P[\!b_{\mathrm{e}}|\mathbb{R}]} { \sum\limits_{b_{\mathrm{e}} \in \Omega} P[\!b_{\mathrm{e}}|\mathbb{R}] }.   $$

An analogous computation is done to determine the contaminant base. The computation for insertions and deletions is similar (see Additional file 1: Methods, Section 1.2).

#### Computation of $P[R_{j} \in \mathbb {E}]$

For the probability that a given fragment *R*_*j*_ is endogenous, denoted as $P[\!R_{j} \in \mathbb {E}]$, our model takes into consideration two factors: deamination patterns and the length of the fragments. Parameters for these two factors are introduced as input to the endogenous caller. Such parameters are re-estimated at each iteration using fragments that support an endogenous base versus a contaminant one (splitEndo in Fig. 2). The splitEndo module will (i) use the output of endoCaller from the previous iteration and separate fragments that support the endogenous or the contaminant base at positions where they differ and (ii) estimate deamination parameters and fit a log-normal distribution on each separated set of fragments independently. Deamination rates are obtained by measuring rates of nucleotide substitution from the reference base at a given position in the fragment and the log-normal parameters are obtained by a maximum-likelihood fit using the fitdistrplus R package. These estimates are fixed throughout a single iteration and are re-estimated by splitEndo in the following one.

Endogenous fragments tend to exhibit higher rates of deamination than contaminant fragments (see Additional file 1: Methods, Section 1.7). In the previous section where contDeam was described, we compared a model that considers deamination and sequencing errors, and another model that solely uses sequencing errors to compute the probability of seeing a particular alignment given the reference as template. In this section, we seek to incorporate the possibility that the template might be a different base than the endogenous one for greater accuracy. Let *E* denote the event that a sequencing error has occurred, *D* the event that deamination has occurred and let ¬ denote the complement of an event (i.e., the event has not occurred). First, we seek to compute the probability of observing the base *r*_*i*_, part of the fragment *R*_*j*_, given that it originated from endogenous base *b*_e_ under a model where substitutions are solely due to sequencing errors. This term, denoted *p*_*n*_(*r*_*i*_), is obtained similarly to Eq. 1 but by considering all four potential endogenous bases *b*_e_ as follows:

(21) $$ \sum_{b_{\mathrm{e}} \in \Omega} (1-P[\neg b_{\mathrm{e}}]) P_{n} [\!r_{i}|b_{\mathrm{e}}]   $$

where *P*_*n*_[*r*_*i*_|*b*_e_] is equal to

(22) $$ \begin{cases}(1-\epsilon_{i})\,P[\!b_{\mathrm{e}} \to r_{i} |\neg E], & \text{if}\,b_{\mathrm{e}} = r_{i} \\ \epsilon_{i}\,P[\!b_{\mathrm{e}} \to r_{i} |E], & \text{if}\,b_{\mathrm{e}} \neq r_{i} \\ \end{cases}   $$

where $P[\neg b_{\mathrm {e}}|\mathbb {R}]$ is the probability of error for endogenous base *b*_e_ as defined in Eq. 20. The nucleotide substitution probabilities given either absence or presence of a sequencing error are computed as described in the contDeam section. Second, we compute the probability of seeing base *r*_*i*_ given endogenous base *b*_e_ if any difference is explained by either deamination or sequencing errors. Like Eq. 2, this probability, denoted *P*_d_[ *r*_*i*_], is computed using

(23) $$ \sum_{b_{\mathrm{e}} \in \Omega} (1-P[\neg b_{\mathrm{e}}]) P_{\mathrm{d}} [r_{i}|b_{\mathrm{e}}]   $$

where *P*_d_[*r*_*i*_|*b*_e_] is equal to

(24) $$ \begin{cases}(1-\epsilon_{i})\,P[\!b_{\mathrm{e}} \to r_{i} |\neg D \cap \neg E ], & \text{if}\,b_{\mathrm{e}} = r_{i} \\ (1-\epsilon_{i})\,P[\!b_{\mathrm{e}} \to r_{i} |D ] +\ \epsilon_{i} P[ \!b_{\mathrm{e}} \to r_{i} |E], & \text{if}\,b_{\mathrm{e}} \neq r_{i}. \\ \end{cases}   $$

Again, the substitution probabilities given either deamination or sequencing error are computed as described in the contDeam section.

We compute the probability that the aligned fragment *R*_*j*_ was observed under a deamination and sequencing error model, denoted *P*[ *R*_*j*_|*M*_deam_], by taking the product for each base $r_{1}, \dots, r_{l} \in R_{j}$ of the term described by Eq. 23. The probability that aligned fragment *R*_*j*_ was observed under a sequencing error model, denoted *P*[ *R*_*j*_|*M*_null_] uses the product of the term described by Eq. 21 where only sequencing errors are considered.

As mentioned previously, endoCaller needs as input a prior, denoted *c*_c_, on the rate of present-day human contamination. Finally, both probabilities are combined with our prior on a fragment being endogenous of 1−*c*_c_ as a posterior probability to obtain the probability that fragment *R*_*j*_ is deaminated:

(25) $$ \frac { (1-c_{\mathrm{c}}) P[\!R_{j} | M_{\text{deam}} ]} { (1-c_{\mathrm{c}}) P[\!R_{j} | M_{\text{deam}} ] +\ c_{\mathrm{c}} P[ \!R_{j} | M_{\text{null}}] }.   $$

Differences in fragment lengths between the endogenous and contaminant sequences can also be informative about contamination. Ancient fragments tend to be shorter than modern contaminating DNA fragments due to degradation of aDNA [1, 29, 30, 32] (see Additional file 1: Methods, Section 1.5). Other studies have modeled the length of aDNA fragments using a log-normal distribution [33]. Here we model the endogenous and contaminant fragment length distributions using two log-normal distributions and infer, using empirical distributions, four parameters, *μ*_endo_,*σ*_endo_,*μ*_cont_ and *σ*_cont_, for the location and scale parameters of the endogenous and contaminant log-normal distributions, respectively. Again, these parameters are estimated by splitEndo at each iteration. The probability that the fragment *R*_*j*_ of length *l* was sampled from the endogenous distribution is given by the probability density function for the log-normal distribution:

(26) $$ P[\!R_{j} \in \text{endo}_{\text{dist}}] = \frac {1} {l \sqrt{2\pi} \sigma_{\text{endo}}} \mathrm{e}^{- \frac{\left(\ln(l) - \mu_{\text{endo}}\right)^{2}} {2 \sigma_{\text{endo}}^{2}} }.  $$

The probability that the fragment is from the contaminant distribution (*P*[*R*_*j*_∈cont_dist_]) is calculated the same way except using the location and scale for that distribution. The posterior probability of both terms is used to compute the probability that fragment *R*_*j*_ pertains to the endogenous distribution using the contamination prior:

(27) $$ \frac { (1-c_{\mathrm{c}}) P[\!R_{j} \in \text{endo}_{\text{dist}}]} {(1-c_{\mathrm{c}}) P[\!R_{j} \in \text{endo}_{\text{dist}}] +\ c_{\mathrm{c}} P[\!R_{j} \in \text{cont}_{\text{dist}}]}.   $$

Finally, the deamination and length probabilities are combined to compute the probability that a fragment is endogenous ($P[\!R_{j} \in \mathbb {E}]$).

### mtCont: mitochondrial contamination estimate

Once the endogenous base and its likelihood have been computed for a given site, a second program takes this information, together with the aligned BAM file of all fragments covering each site, and determines the most likely contaminating genome from the database of possible contaminants as well as the contamination rate (mtCont in Fig. 2). This is achieved by determining the most likely contamination rate using sites where bases in the putative endogenous and contaminant genomes differ. Once this computation is finished for all mitochondrial genomes in the database, the genome with the highest likelihood of being the contaminant is identified (see details in Additional file 1: Methods, Section 1.6).

In the previous section, a fixed contamination prior was supplied to endoCaller and the most likely endogenous and contaminant bases were inferred given the data. In this section, mtCont computes the most likely contamination rate given the data for fixed probabilities for the endogenous and contamination bases, which are provided by endoCaller. As in endoCaller, the deamination rates are entered as input. The contamination estimate generated by contDeam at iteration #1 is recalculated by mtCont in subsequent iterations (see Fig. 2).

For a given position on the mitochondrion, let *b*_e_ be a possible base from the endogenous sample and *c* be a potential base from the contaminant. Let the contamination rate be *c*_r_, defined as the probability of seeing a base from the contaminant at this given position. Therefore, the probability that the base is endogenous is 1−*c*_r_. Like the terms used in the section above, let *R*_*j*_ be a fragment with mismapping probability $m_{R_{j}}$ and let base *r*_*i*_ be its base at the position of interest. The probability of observing *r*_*i*_ given that either *b*_e_ or *c* could have given rise to it, denoted *P*[ *r*_*i*_|*b*_e_,*c*], is

(28) $$ (1-m_{R_{j}}) P[\!r_{i}|b_{\mathrm{e}},c,M] +\ m_{R_{j}} P[\! r_{i}|b_{\mathrm{e}},c,\neg M]   $$

where the probability of being mismapped is defined as in Eq. 15. If the fragment is properly mapped, it can originate from either the contaminant or the endogenous genome. Using the defined contamination rate, we can quantify *P*[*r*_*i*_|*b*_e_,*c*,*M*], the probability of observing *r*_*i*_ given that the fragment was correctly mapped as

(29) $$ (1-c_{\mathrm{r}}) P_{\mathrm{e}} [\!r_{i}|b_{\mathrm{e}},M] +\ c_{\mathrm{r}} P_{c} [\!r_{i}|c,M]   $$

since we either sampled from the contaminant with probability *c*_r_ or from the endogenous base with probability 1 − *c*_r_. The probability of observing the base *r*_*i*_ given it came from either the endogenous material (*P*_e_[ *r*_*i*_|*b*_e_,*c*,*M*]) or the contamination (*P*_*c*_[ *r*_*i*_|*b*_e_,*c*,*M*]) considers sequencing errors and deamination rates. The precise terms found for such quantities are derived as in Eq. 10. The only difference is that a deaminated substitution model is used for the endogenous base whereas a model that only considers sequencing errors is used for the contaminant base.

Let *Ω*^2^ be the set of all possible pairs of nucleotides. For a given contamination rate *c*_r_, the probability (*P*[ *r*_*i*_]) of observing the base *r*_*i*_ is obtained by marginalizing over each possible contaminant and endogenous base:

(30) $$ \sum\limits_{b_{\mathrm{e}},c \in \Omega^{2}} P[\!r_{i}|b_{\mathrm{e}},c] P[\!b_{\mathrm{e}},c]   $$

where the term *P*[ *r*_*i*_|*b*_e_,*c*] is defined in Eq. 28. The combined probability of *b*_e_ being the endogenous and *c* being the contaminant base is given by *P*[ *b*_e_,*c*]=*P*[ *b*_e_]*P*[ *c*]. The prior on the endogenous base *P*[ *b*_e_] is one minus the probability that *b*_e_ is not the endogenous base, a quantity defined by Eq. 20. The probability *P*[ *c*] is defined by the probability of having nucleotide *c* in the putative contaminant mitochondrial sequence.

The total likelihood is obtained by the product of Eq. 30 for every fragment. This likelihood is computed for every contamination rate between 0 and 100 % assuming a uniform prior on the contamination rate and for each mitochondrial genome in the set of putative contaminants. Finally, the contaminant genome is determined and the contamination rate with the highest posterior probability, as well as a 95 % confidence interval, is produced.

## Discussion

aDNA analyses have typically decoupled reconstruction of the endogenous mitochondrial genome from quantification and characterization of present-day human contamination. Since these two tasks are interdependent, we argue that consensus calling and contamination estimation should be performed iteratively to achieve the most accurate results. Current approaches to determining the endogenous mtDNA sequence are very dependent on the amount of contamination. In samples with low present-day human contamination, a consensus sequence is usually called using all sequences, whereas for highly contaminated samples, only deaminated fragments are used. However, there is no clear contamination cut-off to determine which strategy should be used. Schmutzi can be applied to samples with either low or high levels of contamination thereby obviating this decision.

We have presented here empirical and simulated datasets demonstrating that schmutzi outperforms a number of existing approaches to consensus sequence calling and contamination estimation over a wide range of contamination rates and coverages. Our simulations were conducted using empirical fragment length distributions and deamination rates. It is trivial to see that higher deamination rates can enable end users to infer with greater confidence the endogenous sequence of even highly contaminated samples. We note that absence of deamination will yield incorrect estimates of contamination. Since deamination is the primary feature used to distinguish endogenous from contaminant bases, treatment with full uracil-DNA glycosylase [34] is also likely to impact negatively the estimation of contamination and result in an incorrect endogenous consensus call at high levels of contamination. We, therefore, recommend using schmutzi only for samples with either no, or partial, uracil-DNA glycosylase treatment for potentially contaminated samples. It is important to note that the number of parameters and their range hinder us from making simple general statements about the amount of coverage or extent of deamination required for accurate estimates of present-day human contamination or accurate inference of the endogenous genome sequence. Although our study focused on human mitochondrial aDNA data, schmutzi can be applied to any other haploid aDNA dataset for which a reference genome is available. This includes non-human mitochondrial genomes, as well as viral, bacterial and chloroplast genomes.

Although many groups have implemented ad hoc methods to assess contamination, there are few available software implementations. We compared schmutzi to contamMix, a previously used maximum-likelihood method described in [14]. The predicted contamination rates produced by our algorithm are more accurate than those produced by this method on simulated data (see Additional file 1: Results, Section 2.3.6). Although the true contamination rate is not known for most ancient datasets, we have shown that our estimates are also consistent with contamination measured in empirical datasets using methods relying on diagnostic positions. While the approach of taking diagnostic positions is suitable for archaic humans like Neanderthals, it is not readily applicable to early modern humans, who have few fixed differences to present-day humans. Schmutzi’s modeling of mismatches due to deamination, sequencing errors and mismapping results in greater accuracy than simply estimating a single error parameter.

Our endogenous consensus call shows a significant dependence on the prior, which is calculated based on the deamination patterns only for the first iteration (contDeam). We interpret this as evidence that a reasonable estimate for contamination can be obtained from deamination. For contDeam, we have also evaluated the impact on the final estimate due to biases like insufficient deamination and having deamination for contaminant fragments (see Additional file 1: Results Section 2.3.4). We do, however, notice that the contamination estimate improves incrementally during iteration of consensus calling and contamination estimation, suggesting that additional information is available in the mitochondrial endogenous consensus. This is particularly useful for low coverage samples.

Schmutzi accurately infers the endogenous ancient genome sequence from unfiltered ancient sequence data. This is of particular importance when the contamination is high. Interestingly, schmutzi is also more accurate than approaches that reduce contamination by using only deaminated fragments to call the consensus. Such approaches substantially reduce the number of fragments available for calling the consensus, which may explain why schmutzi is marginally better at determining the consensus sequence.

Although schmutzi performs well for both simulated and empirical data, a few artifacts are not currently modeled in the software. First, it is possible that there are multiple present-day human contaminants. At low contamination rates with multiple contaminants, schmutzi will underestimate the contamination, but the inference of the endogenous consensus sequence should not be affected. However, at high contamination rates, multiple contaminants make the inference of the endogenous sequence and estimation of the contamination extremely difficult, since the endogenous and contaminant alleles do not follow the expected distributions. Second, inclusion of misaligned microbial sequences and mitochondrial heteroplasmy are also not currently considered in the computation, though the empirical data suggest that schmutzi is not particularly sensitive to these. Lastly, the use of target enrichment approaches with DNA probes that are closer to the contaminant than to the endogenous sequence may cause differences in allele sampling, and may lead to incorrect consensus calls (see Fig. 4 and Additional file 1: Section 2.2.3 for further discussion about the capture bias).

Schmutzi is sensitive to the divergence between the actual contaminant and the closest record in the database of putative contaminants. If this divergence is very large (e.g., more than 30 mismatches), contamination will be underestimated.

When contamination rates are high, the predicted contaminant can be inferred at high resolution. This enables the program to use this predicted contaminant as a database record for the quantification of mitochondrial contamination (see Additional file 1: Results). This is not feasible at low contamination rates, where the prediction of the contaminant mtDNA is poor. Our method does not currently use phylogenetic information to infer the endogenous and contaminant sequences. Although our approach works well empirically, the use of phylogenetic information could provide additional power for obtaining contamination estimates in very low coverage samples.

In conclusion, we have described an algorithm that infers the endogenous mitochondrial genome sequence from an aDNA sample, even in the presence of high contamination. We have applied this to the reconstruction of mitochondrial genomes for archaic and early modern humans and show that it is possible to quantify accurately contamination from present-day individuals.

## Additional file

Additional file 1 **Supplementary material.** (PDF 2232 kb)

## Abbreviations

aDNA: Ancient DNA
MIA: Mapping iterative assembler
