# Supplementary material for: Schmutzi: estimation of contamination and endogenous mitochondrial consensus calling for ancient DNA
Source: Genome Biol. 2015 Oct 12;16:224. doi: 10.1186/s13059-015-0776-0 (PMC4601135; doi:10.1186/s13059-015-0776-0)
Supplement: Supplementary file 1 — Supplementary material. (PDF 2232 kb) [file 13059_2015_776_MOESM1_ESM.pdf]

# Schmutzi: contamination estimate and endogenous mitochondrial consensus calling for ancient DNA.

Additional file 1

Gabriel Renaud Viviane Slon Ana T. Duggan
Janet Kelso

## Contents

**S1 Additional file 1: Methods** **3**
S1.1 Mapping . . . 3
S1.1.1 Handling circular references . . . 3
S1.1.2 Mapping sensitivity . . . 3
S1.2 Identifying endogenous insertions and deletions . . . 4
S1.2.1 Deletions . . . 4
S1.2.2 Insertion . . . 5
S1.3 Endogenous consensus calling with multiple contaminants . . . 5
S1.3.1 Calling the endogenous nucleotide . . . 6
S1.3.2 Insertions . . . 6
S1.3.3 Deletion . . . 7
S1.4 Comparison to existing methods . . . 7
S1.5 Distribution of the endogenous and contaminant fragment size . 8
S1.6 Database of putative contaminants . . . 8
S1.7 Test data . . . 9
S1.7.1 B9687 . . . 9
S1.7.2 B9688 . . . 11

**S2 Additional file 1: Results** **16**
S2.1 Mitochondrial mapping strategies . . . 16
S2.2 Empirical data . . . 18
S2.2.1 Contamination estimate based on deamination . . . 18
S2.2.2 Contamination estimate based on divergent bases . . . 19
S2.2.3 Endogenous mitochondrion consensus call . . . 19
S2.2.4 Contaminant mitochondrion consensus call . . . 21
S2.2.5 Contamination estimate for 4 ancient mtDNA studies from different laboratories . . . 24
S2.3 Simulated data . . . 27
S2.3.1 Endogenous mitochondrion consensus call . . . 27
S2.3.2 Contaminant mitochondrion consensus call . . . 29
Effect of lower coverage . . . 29
S2.3.3 Contamination estimate based on deamination . . . 30
Full datasets . . . 31
Subsampled datasets . . . 31
S2.3.4 Biases affecting the prior contamination estimate based on deamination . . . 38
Impact of low deamination rates . . . 38

Impact of deamination for contaminating fragments . . . 38
Independence tests for deamination on each end . . . . . 38
S2.3.5 Contamination estimate based on divergent bases . . . . . 43
Full datasets . . . . . . . . . . . . . . . . . . . . . . . . . . 43
Subsampled datasets . . . . . . . . . . . . . . . . . . . . . . 43
S2.3.6 Comparison to existing methods . . . . . . . . . . . . . . . 49
S2.3.7 Multiple contaminants . . . . . . . . . . . . . . . . . . . . . 51

# S1 Additional file 1: Methods

## S1.1 Mapping

### S1.1.1 Handling circular references

Prior to performing the endogenous consensus call, all fragments from both the contaminant and endogenous genomes must be aligned to a reference genome. Most aligners for next-generation sequencing (NGS) do not allow for circular reference genomes leading to spurious drops of coverage around the ends. To circumvent this, the first 1000 basepairs of the mitochondrial reference can be appended at the end and used as new reference. A script [1] folds alignments spanning the end of the mitochondrion back to the beginning of the reference. To illustrate the corrective effect on coverage, a set of 1M fragments of 100 bp from the revised Cambridge Reference Sequence (rCRS) mitochondrion (GenBank: NC_012920) were simulated. Random coordinates were simulated using a uniform distribution and fragments were allowed to span the sequence junction as to reflect circularity. Fragments were simulated using in-house programs [2]. The fragments were aligned to the default reference using BWA v0.5.10[1]. In a separate set, the fragments were aligned to the extended reference genome and fragments spanning the junction of the genome were folded back. Results show the corrective effect of this strategy (see Section S2).

### S1.1.2 Mapping sensitivity

The lack of sensitivity of the aligner for highly divergent loci can create a bias towards having a greater proportion of contaminant fragments aligning than the average across the mitochondrial genome (see Figure S1). This is particularly true for highly divergent samples like the Denisovan mitochondrion [2]. To evaluate whether currently used aligners could cause such a bias, aDNA fragments from the Denisovan mitochondrial genome [3] were simulated again using the strategy described above. The simulated length of the fragments was taken from empirical distributions (see Section S1.5). Deamination rates were added using the deamination rates from the single-stranded libraries from [3]. Sequencing errors were added along with representative quality scores using empirical rates obtained using Illumina reads of PhiX control. The fragments were aligned to the extended human mitochondrial reference using both BWA v0.5.10 (with "-n 0.01 -o 2 -l 16500", optimized for increased sensitivity for ancient DNA [4]) and SHRIMP v2.2.3[5] ("-N 5 -o 1 –single-best-mapping –sam-unaligned –fastq –sam –qv-offset 33"). Again, fragments spanning the junction of the genome were wrapped back at the beginning. The impact of the mapping algorithm used on coverage versus genome divergence are described in Section S2.

[1] https://github.com/udo-stenzel/biohazard/
[2] https://github.com/grenaud/simulateAncientDNA
[3] GenBank: FN673705.1

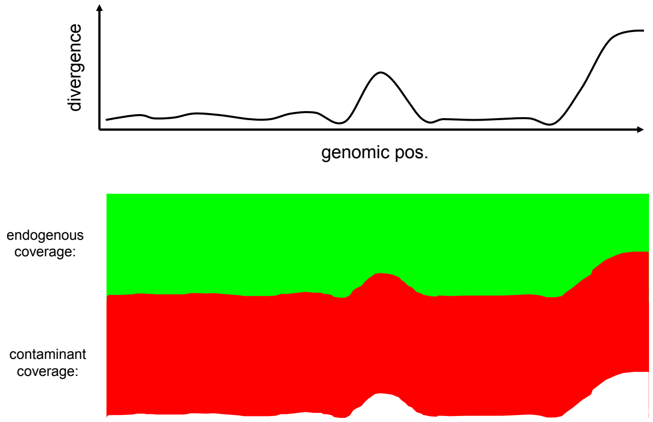


Figure S1: Schematic of the effect of using a low sensitivity aligner to the human mitochondrial reference in regions of high divergence. The endogenous ancient DNA has higher divergence to the reference than the contaminant creating the possibility that the endogenous fragments will not align due to a higher edit distance. Although the distribution of the fragments will be representative of the contamination rate in regions of low divergence, contaminant fragments may overtake endogenous ones in regions of high divergence. A paucity of endogenous fragments could lead to an inability to call certain regions, generating spurious signals and an overestimate of the contamination rate.

## S1.2 Identifying endogenous insertions and deletions

For indels, we consider two separate cases:

- A deletion in our sample (which could also be an insertion in the reference)
- An insertion in our sample (which could also be a deletion in the reference)

Each case is described separately in the sections below. In both cases, we cannot know *a priori* without using phylogenetic information in which lineage the indel occurred. We consider the error rate of indels to be a constant $\epsilon_{indel}$ for both cases. This constant is defined from the literature on sequencer-specific error rates [6]. Given than an indel was present in the fragment, we consider it to be present in the original fragment with probability $1 - \epsilon_{indel}$ and absent with probability $\epsilon_{indel}$. As in the inference of a single nucleotide, the computation is different depending on whether we consider a single contaminant or multiple ones.

### S1.2.1 Deletions

A deletion refers to missing nucleotides with respect to the reference in either the contaminant or the endogenous genome. This could be due to a deletion in the lineage leading to our sample or an insertion in the one leading to the reference.

Given that a deletion is observed, four different scenarios need to be considered:

- Both endogenous and contaminant genomes have the deletion

- Only the endogenous genome has the deletion
- Only the contaminant genome has the deletion
- Neither the contaminant nor the endogenous genome have the deletion and the observation was due to a sequencing error

Let $\mathbb{E}$ be the set of fragments came from the endogenous mitochondrial genome such that $\mathbb{E} \subseteq \mathbb{R}$. The observation of a fragment with or without a deletion changes the likelihood for each possibility. For instance, for the first case, the observation of a fragment $R_j$ with the deletion gives the following term in the product:

$$(1 - m_{R_J})[P[R_j \in \mathbb{E}] \cdot (1 - \epsilon_{indel}) + (1 - P[R_j \in \mathbb{E}]) \cdot (1 - \epsilon_{indel})] \quad (1)$$

where $m_{R_J}$ is the probability that fragment $R_j$ is mismapped and where $P[R_j \in \mathbb{E}]$ is the probability that fragment $R_j$ is endogenous (defined in the methods section of the main manuscript). As both genomes contain the deletion, the probability of observing the fragment $R_j$ is the probability of having correctly detected the deletion in either of the two cases. If the fragment does not have the deletion, still under the assumption that both endogenous and contaminant genomes have the deletion, the term becomes the probability that either one contains an error:

$$(1 - m_{R_J})[P[R_j \in \mathbb{E}] \cdot \epsilon_{indel} + (1 - P[R_j \in \mathbb{E}])) \cdot \epsilon_{indel}] \quad (2)$$

as the fragments falsely called it in both cases. A similar computation is done for the remaining three possibilities but where the indel error term is used differently depending on which genome is believed to have the deletion. Finally, the possibility with the maximum posterior probability is used to produce both the endogenous and contaminant genomes. The error probability on that call is computed by the ratio of the sum of the probabilities for all three least likely scenarios over the sum of all probabilities.

#### S1.2.2 Insertion

Insertions are produced in a manner similar to deletions. For the deletion case, we considered the likelihood of a nucleotide being present or absent at a specific position. In the case of insertions, the possibility of having various nucleotides being inserted at a specific position is considered. The likelihood for each putative inserted sequence and the absence of an insertion is calculated.

We consider a bi-dimensional matrix for all possible insertions for both the endogenous and contaminant genomes. Each cell represents a specific model where either genomes could have a given insertion. The likelihood is computed using a product over all fragments using terms analogous to expressions 1 and 2 depending on which of the two genomes has the insertion for that given model. Finally, the most likely model is retained. For calling the endogenous consensus genome, the error probability is marginalized over each possible contaminant insertion and vice-versa for the contaminant consensus calling.

### S1.3 Endogenous consensus calling with multiple contaminants

Multiple contaminants with equal contributions represent a more complex problem for consensus calling, compared to a single one (see Figure S2). Our results show that schmutzi yields good results (a reliable consensus endogenous genome) at low contamination rates but not at higher ones (see Section S2).

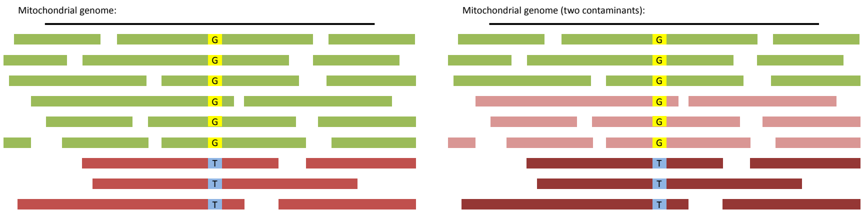


Figure S2: Schematic of alignments to the mitochondrial genome where green lines represent endogenous fragments and red lines, the contamination. However, depending on whether there is a single source of mitochondrial contamination (left) or multiple ones (right), the distribution of the bases at a segregating site can change. Given that the contamination rate is $\frac{1}{3}$ for the single contaminant scenario, inferring the endogenous and contaminant bases is straightforward, as the relative number of each base follows the expected distribution. However, in the figure to the right, knowing that the contamination rate is $\frac{2}{3}$ does not translate into observing this fraction of a particular contaminant base.

#### S1.3.1 Calling the endogenous nucleotide

Given that the fragment $R_j$ was correctly mapped, it originated either from the endogenous or the contaminant genome. Let $P[R_j \in \mathbb{E}]$ be the probability that the fragment came from the endogenous genome (refer to methods section of the main document for further information). The probability that the fragment stemmed from the contaminant genome is simply $1 - P[R_j \in \mathbb{E}]$.

We seek to compute the probability of observing $r_i$ on fragment $R_j$ given that $b_e$ is the putative endogenous base. Let $M$ be the event that $R_j$ was correctly mapped. In the case where the fragment $R_j$ is a contaminant fragment, no information can be obtained on the probability of observing base $r_i$ given $b_e$ hence the uniform prior for nucleotides is used:

$$P[r_i|b_e, M] = P[R_j \in \mathbb{E}] \cdot P_e[r_i|b_e] + (1 - P[R_j \in \mathbb{E}]) \cdot \frac{1}{4} \tag{3}$$

This term is similar to the way $P[r_i|b_e, b_c, M]$ is computed for the single contaminant case with the exception of the lack of a single contaminant base $b_c$. The remaining computations are identical to the case with a single contaminant.

#### S1.3.2 Insertions

We compute the likelihood of all observed insertions at a given position, assuming that unobserved insertions have a negligible likelihood. We also consider the likelihood for not having an insertion. For a given insertion, if it is observed in a fragment, the term in the product becomes the following expression:

$$(1 - m_{R_J}) \cdot P[R_j \in \mathbb{E}] \cdot (1 - \epsilon_{indel}) \tag{4}$$

where $m_{R_J}$ is the probability of mismapping for that given fragment and $P[R_j \in \mathbb{E}]$ is the probability that $R_j$ is endogenous. However, for the remaining insertions, the following term is used:

$$(1 - m_{R_J}) \cdot P[R_j \in \mathbb{E}] \cdot \epsilon_{indel} \tag{5}$$

The most likely insertion is produced and the error probability is defined as the ratio of the sum of the probabilities for possible insertions minus the most likely over the sum of all probabilities.

### S1.3.3 Deletion

We consider the likelihood of two scenarios: either the endogenous genome has a deletion or it does not. Again, using the assumption of independence of observation for each fragment, we multiply the likelihood for each fragment independently for each of these two possibilities. For the former, where the endogenous genome has a deletion, for each fragment $R_j$ with a deletion, the term in the product becomes the term defined in equation 4. For the second scenario, where the endogenous does not have the deletion and the fragment $R_j$ has the deletion, the expression used is defined by equation 5. If fragment $R_j$ does not have a deletion, the two previously defined terms are swapped for one another in the products. Finally, a deletion in the endogenous consensus is produced if the likelihood of such an event exceeds the likelihood of not having a deletion. The error probability is computed by taking the ratio of the second most likely scenario over the sum of the probabilities for both possibilities.

## S1.4 Comparison to existing methods

Although there have been descriptions of methods to estimate the contamination rate, there is currently no software implementation of an algorithm to estimate contamination for aDNA samples that is widely available for download. To provide a comparison to such methods, the maximum likelihood model described in [7, 8] was implemented and used on our simulated datasets. The predicted contamination rate was compared to the simulated one.

Briefly, a rate of sequencing error denoted $\epsilon$ is estimated using monomorphic regions of a set of mitochondrial genomes. The fragments are aligned against the endogenous consensus call and a database of 311 potential contaminant mitochondria as described in the original methodology. Since our simulations used a single contaminant, a single genome was used in the database. We ran the method once using the closest mitochondrial genome in the database and once more using the same contaminant used in the simulations.

For a read $R_i$ aligned to the endogenous genome, we compute $M_{i,e}$ and $N_{i,e}$ for the number of matches and mismatches respectively. The read is also re-aligned to the contaminant genome and the same analogous quantities, $M_{i,c}$ and $N_{i,c}$ are computed. Given the error rate $\epsilon$, the number of matches and mismatches to the endogenous genome, we compute the probability of observing $R_i$ aligned to the endogenous mitochondrion as:

$$\binom{M_{i,e} + N_{i,e}}{N_{i,e}} (1-\epsilon)^{M_{i,e}} (\epsilon)^{n_{i,e}} \tag{6}$$

In the original description, a vector of probabilities describes the probability that read $R_j$ came from each possible contaminant genome in the database and the endogenous mitochondrial genome. This vector is used to compute the probability of observing read $R_j$. In our simulations, as we have two genomes, this expression becomes:

$$(1-c) \cdot \binom{M_{i,e} + N_{i,e}}{N_{i,e}} (1-\epsilon)^{M_{i,e}} (\epsilon)^{n_{i,e}} + c \cdot \binom{M_{i,e} + N_{i,c}}{N_{i,c}} (1-\epsilon)^{M_{i,c}} (\epsilon)^{n_{i,c}} \tag{7}$$

where $c$ is the predicted contamination rate. Finally, the most likely contamination rate given the data is produced by assuming that each fragment represents independent observations as described in [7]. As the method requires the endogenous consensus call, the mitochondrial genome produced by PMDtools and htslib was used as they represent the state of current methods. As the target contamination rate, we used the number of contaminant fragments over the total

as the method operates on a per fragment basis rather than on per nucleotide basis.

## S1.5 Distribution of the endogenous and contaminant fragment size

It was previously suggested in the literature that endogenous and contaminant fragments might have different size distributions where the endogenous fragments are shorter than the contaminant fragments [9, 10]. To measure this, we analyzed the fragments from the Sima de los Huesos hominin [11] that aligned to the mitochondrial genome. As it was heavily contaminated, fragments could be separated into those supporting an endogenous or a contaminant base using diagnostic positions that supported an archaic hominin base or a present-day human one. The size distribution for both was plotted (see Figure S3).

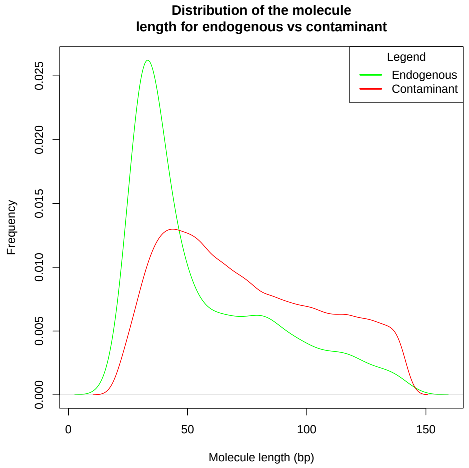


Figure S3: Size distribution of the endogenous (green) versus contaminant (red) fragments in the Sima de los Huesos sample.

## S1.6 Database of putative contaminants

To predict accurate rates of contamination, we require a database of human mitochondrial sequences that are representative of the natural diversity while restricting the total number of sequences due to the computational overhead. As two nearly identical mitochondrial sequence will yield the same contamination rate, having the same sequence twice in the database will result in redundant computations. We downloaded every human mitochondrial sequences from Genbank and performed a multiple sequence alignment using mafft v7.017b [12] due to its speed and multithreading options. All pairwise sequence distances were computed. We pruned the results according to a minimal pairwise edit distance as to have a non-redundant database of 197 records (see Tables S1 and S2). This database is distributed as part of the software package.

Furthermore, the "–usepredC" option in the overall wrapper script allows the user to introduce the predicted contaminant as a database record. This option is recommended for cases where the contamination is very high thus allowing for adequate characterization of the contaminant mitochondrial genome, assuming a that a single contamination source is responsible for most of the contaminating present-day human fragments. As this is not known in advance, we recommend to run the wrapper once with this option and once without.

## S1.7 Test data

Our algorithm was tested on simulated data and empirical ancient DNA datasets as well. We sought to test schmutzi's ability to call the endogenous mitochondrial genome and, assuming that the contamination stemmed from a single source, the accuracy in calling the contaminant's mitochondrial genome as well. To show our robustness to contamination, we sought highly contaminated samples. Further, we sought to measure whether the contamination estimate from schmutzi would be within the estimates obtained by simple contamination determination based on diagnostic positions.

We tested schmutzi on two heavily contaminated biological aDNA datasets. These aDNA datasets (B9687 and B9688) described by [13] pertained to the same Mezmaiskaya Neanderthal individual described in [14]. The latter had the advantage of stemming from an extraction with low amounts of present-day human contamination (0.6%). Therefore, the endogenous consensus call should be identical to the Mezmaiskaya mitochondrial genome (GenBank: FM865411). The contaminating genome however, was not characterized.

The total number of fragments and bases aligning to the mitochondrial reference was calculated (see Table S3). Using diagnostic positions for Neanderthal mitochondrial sequences, the number of contaminant and endogenous fragments was tallied (see Table S4). A contamination estimate could be computed by using the ratio of contaminant fragments over the sum of fragments that were flagged as either contaminant or endogenous. Further, this estimate was recomputed by using the sum of the nucleotides instead of the number of fragments. This led to a different contamination estimate for the first sample as there is a difference in length between endogenous and contaminant fragments (see Section S1.5). Maximum likelihood [15] phylogenetic inference was performed using phylip [16] v 3.69 with default parameters using the mitochondrial genomes enumerated in Table S5. Multiple sequence alignments that were used as input were obtained from prank[17] v 140603.

### S1.7.1 B9687

The details of the experimental procedures for the B9687 samples are found in [13]. Briefly, two extracts of the Mezmaiskaya 1 individual were prepared from 107 mg (extract ID: E734) and 90 mg (extract ID: E373) bone powder using the extraction protocol described in [18]. Sequencing libraries of the extracts were generated using single-stranded library preparation method [19] and double indexing was performed on the libraries [20]. All libraries were subsequently enriched for mitochondrial DNA using human mitochondrial DNA probes following the protocol detailed in [7].

For the B9687 sample, the coverage is the highest among our empirical samples at 710X (see Table S3). Aligned fragments were separated according to whether they stemmed from the endogenous (Neanderthal) or the contaminant (present-day human) mitochondrial genomes using 111 diagnostic positions (fixed sites between 7 Neanderthals and 21 present-day humans) on the mitochondrial reference. This separation into two sets was used to quantify contamination and yielded an estimate in the 43-45% range depending on the metric used (see Table S4). An analysis of the length distribution of the endogenous and contaminant fragments revealed an excess of fragments with approximately the same size as the sequencing read length (see Figure S4). After communication with the authors, this effect is unlikely to stem from library preparation but is more likely an artifact of the extraction procedure. Other libraries prepared using the same protocol does not show this enrichment of fragments with the same size as the length of sequencing. This entails that the use of length will not help our algorithm in gaining greater power to recognize the endogenous

base. Deamination patterns were measured on both the fragments labeled as endogenous and those identified as contaminant (see Figure S5). As the deamination rates of the endogenous fragments are several fold higher than the ones found for the contaminant ones, our algorithm can use this information to disentangle which base is likely to be endogenous and which is likely to be the contaminant one. Furthermore, the deamination rates for the contaminants are very low, enabling the possibility of getting an estimate of contamination based on deamination rates alone (see Methods Section in the main document).

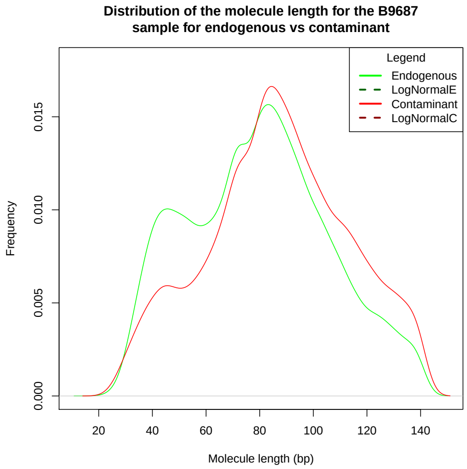


Figure S4: Density plot of the size of the fragments identified as endogenous (Neanderthal in green) and contaminant (present-day human in red) in the B9687 sample.

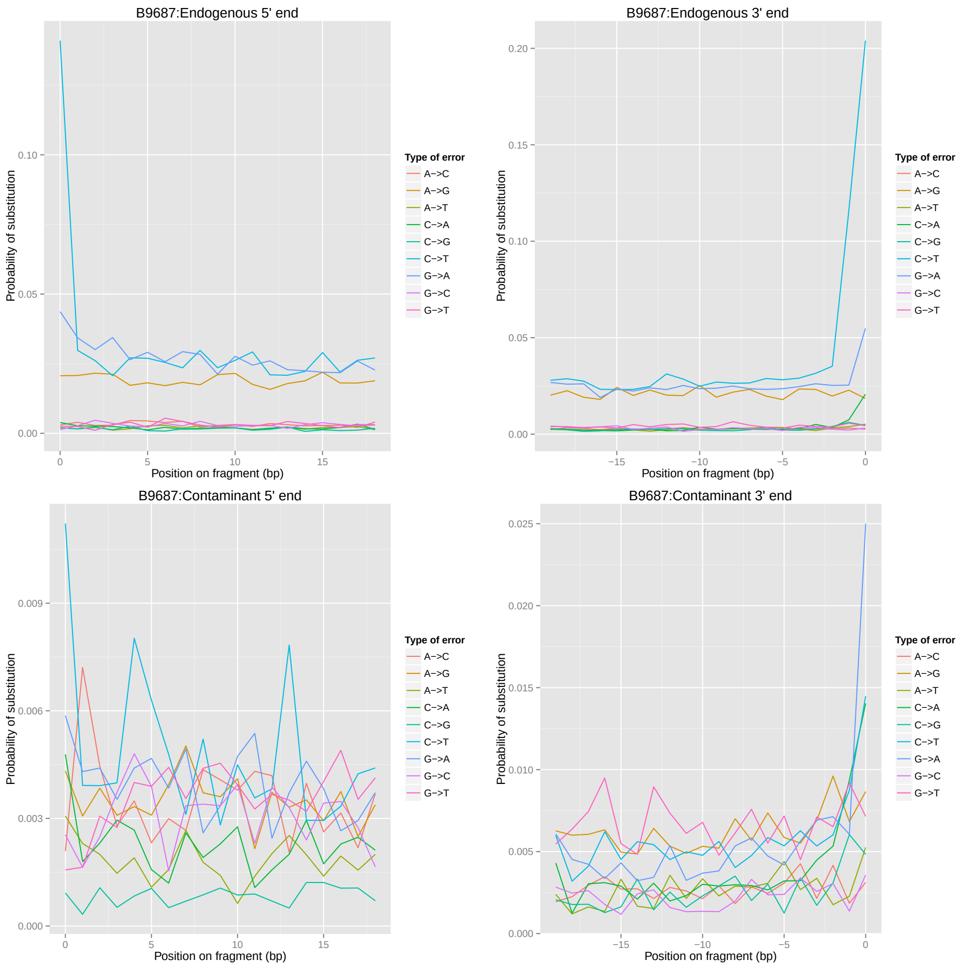


Figure S5: Deamination patterns for the fragments identified as endogenous and contaminant in the B9687 sample for the 5' end (left) and 3' end (right). The fragments were identified as either Neanderthal (top) or present-day human (bottom).

### S1.7.2 B9688

B9688 was the second sample described in [13]. It was sequenced in a similar way as B9687, however, coverage was slightly lower at 635X. Using the same diagnostic positions as B9687, aligned fragments were split into two sets, those supporting a Neanderthal base and those supporting a present-day human one. Contamination estimates for this sample were between 48% and 50%, higher than the B9687 sample (see Table S4). A measure of fragment length revealed the same enrichment for fragments with the same length as the original fragment previously seen in B9687 (see Figure S6). Contaminant fragments also showed very low rates of deamination (see Figure S7).

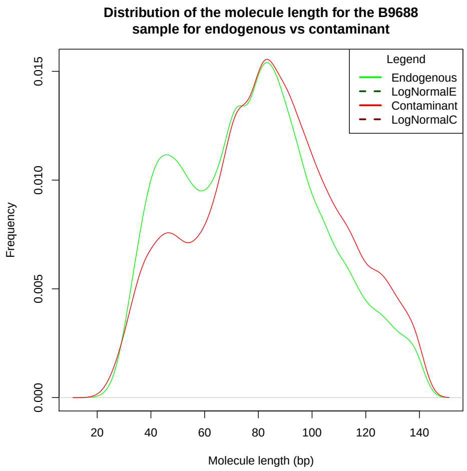


Figure S6: Density plot of the size of the fragments identified as endogenous (Neanderthal in green) and contaminant (present-day human in red) in the B9688 sample.

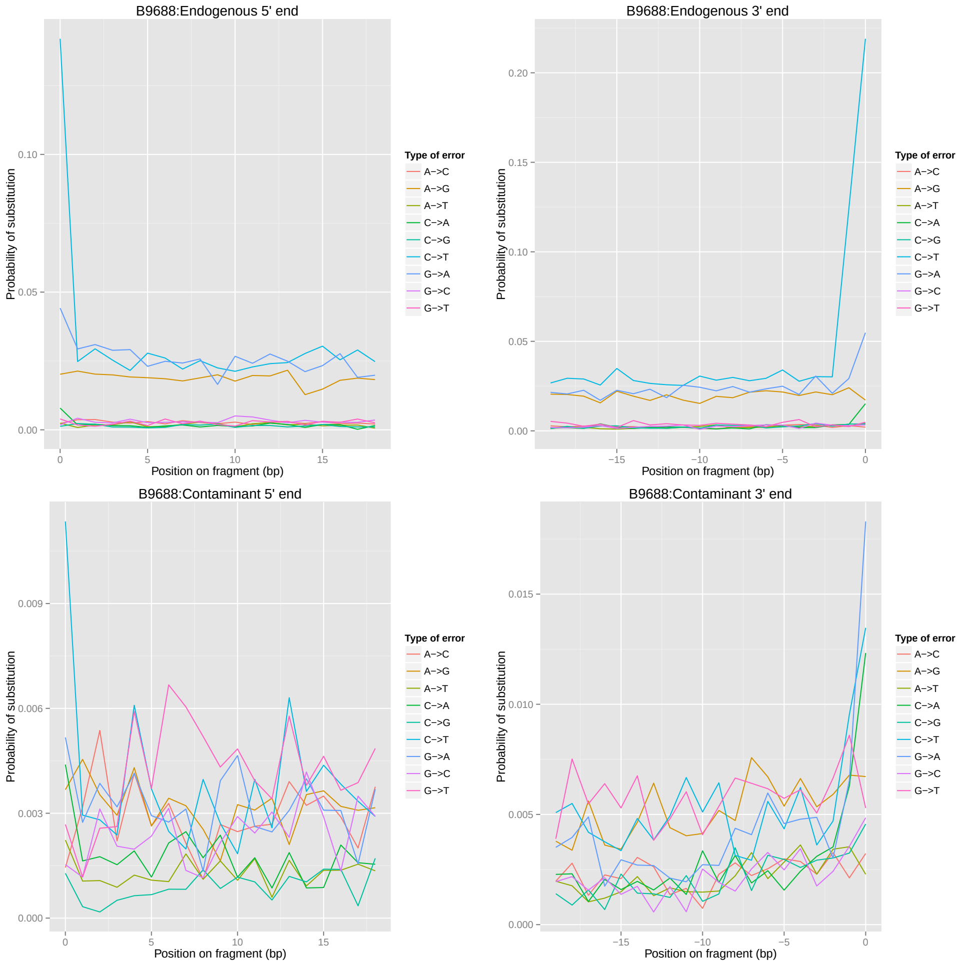


Figure S7: Deamination patterns for the fragments identified as endogenous and contaminant in the B9688 sample for the 5' end (left) and 3' end (right). The fragments were identified as either Neanderthal (top) or present-day human (bottom).

| Sample ID | HaploGrep Quality | Predicted Haplogroup | Sample ID (cont.) | HaploGrep Quality (cont.) | Predicted Haplogroup (cont.) |
|---|---|---|---|---|---|
| JQ703873 | 94.1 | A2i | GQ301880 | 87.1 | M22b |
| KC711027 | 89.5 | B2a1 | FJ543105 | 96.9 | M23 |
| AP008393 | 95.1 | B4c1b1a | KJ154498 | 96.5 | M27a |
| DQ834259 | 63.3 | B4c1b2c | KJ154685 | 86.5 | M27b |
| AP008788 | 91.2 | B4f | KJ154771 | 96.0 | M27c |
| KF540901 | 83.2 | B5a2a2 | KJ154541 | 93.7 | M28a |
| AP008273 | 96.7 | B5b1a2 | DQ137407 | 98.7 | M29a |
| FJ951464 | 95.3 | C5b1a | KC990685 | 75.0 | M2a1a |
| FJ951600 | 85.9 | D4 | EU443449 | 98.5 | M2b3a |
| FJ858886 | 89.6 | D4b1 | KC911426 | 91.1 | M2c |
| FJ168748 | 97.0 | D4h3a9 | AY950293 | 96.7 | M31a1b |
| FJ951465 | 100.0 | D5a2a2 | GQ389779 | 98.4 | M32c |
| KJ154788 | 98.8 | E1b1 | HM030510 | 91.0 | M33b1 |
| KF849964 | 94.4 | F1a1d | JX462713 | 97.0 | M33d |
| KC252477 | 100.0 | F3b1a | AY922304 | 98.3 | M34a1a |
| KF451331 | 93.9 | F4a2 | FJ383405 | 89.4 | M38b |
| KF148403 | 92.9 | G2a1 | KC990670 | 72.0 | M42'74 |
| HM454265 | 92.2 | I1a | DQ404443 | 83.3 | M42a |
| JQ797764 | 94.8 | J1b1a2b | FJ380216 | 82.3 | M42b |
| JQ797929 | 96.2 | J2a2b | FJ383746 | 89.4 | M42b1a |
| JQ702671 | 95.9 | K1a1b1a | KC990667 | 63.0 | M5 |
| KJ185548 | 98.9 | L0a1b1a1 | JX289098 | 91.5 | M50a2 |
| EF184602 | 94.8 | L0a2 | GQ301882 | 97.2 | M51a2 |
| KC533465 | 87.7 | L0a2a2a | FJ383491 | 94.0 | M52b1a |
| KJ185995 | 84.5 | L0a'b | FJ383439 | 94.6 | M53b |
| EU092936 | 94.1 | L0b | KC896622 | 97.3 | M55 |
| KF672800 | 89.8 | L0b | FJ383762 | 87.9 | M57a |
| KC346214 | 97.6 | L0d1b2b2a | JX289110 | 81.9 | M58 |
| KC533490 | 94.4 | L0d1c1a | DQ834260 | 79.4 | M59 |
| KC346193 | 98.9 | L0d2a1c | KC505104 | 87.6 | M59 |
| KC345912 | 98.9 | L0d2b1a1a | JQ446396 | 83.7 | M5a1a |
| KC346210 | 97.3 | L0d2c1a | KC990648 | 72.3 | M5a2a1a |
| KC533475 | 98.8 | L0d3b | FJ383550 | 84.1 | M5b2b1 |
| EF184595 | 82.6 | L0f | FJ544233 | 96.6 | M62b2 |
| EF184598 | 87.4 | L0f | KC887484 | 96.9 | M68a1a |
| EU092870 | 90.2 | L0f1 | HM596653 | 79.1 | M69 |
| KJ185400 | 88.1 | L0f1 | FJ383302 | 93.7 | M6a1a |
| EF184596 | 85.3 | L0f2a | GQ119039 | 94.2 | M73a |
| EF184599 | 91.0 | L0f2a | HM030520 | 88.0 | M74b |
| EF184597 | 97.8 | L0f2a1 | HM030540 | 90.6 | M75 |
| EU092786 | 100.0 | L0f2b | HM030525 | 81.1 | M76 |
| KC345794 | 100.0 | L0k1a2 | AP009443 | 98.0 | M7a1b2 |
| KM101649 | 98.4 | L1b1a4 | KF540526 | 99.0 | M7b1a1i |
| KC533514 | 87.4 | L1c1 | KC252522 | 88.1 | M8a3a |
| HM771141 | 98.1 | L1c1a1a1a | JX289130 | 89.1 | M91a |
| JX303768 | 90.5 | L1c1a2 | KC887486 | 100.0 | M91b |
| KJ185481 | 95.0 | L1c1b | JN048455 | 60.7 | N10 |
| JQ701901 | 92.3 | L1c1c | HM030542 | 84.3 | N10a |

Table S1: Mitochondrial sources of contamination provided with the software.

| Sample ID | HaploGrep Quality | Predicted Haplogroup | Sample ID (cont.) | HaploGrep Quality (cont.) | Predicted Haplogroup (cont.) |
|---|---|---|---|---|---|
| HM771166 | 91.9 | L1c1d | HM030500 | 98.8 | N10b |
| KJ185466 | 92.1 | L1c2a2 | KF540803 | 82.6 | N11a |
| EU092718 | 94.3 | L1c3a1a | GU733776 | 97.9 | N11b |
| KC257334 | 97.1 | L1c3b2 | JN226143 | 85.9 | N13 |
| HM771117 | 97.8 | L1c4a | JQ705527 | 94.9 | N1a1a1a2 |
| JX303797 | 98.5 | L1c5 | JQ704073 | 94.2 | N1a3a1 |
| EU273489 | 90.0 | L1c6 | EF661011 | 97.7 | N1b1a2 |
| JQ044836 | 90.4 | L1c6 | KC867135 | 99.7 | N3a |
| EF184581 | 77.0 | L2'3'4'6+ | GU480021 | 76.4 | N5a |
| JQ045090 | 99.2 | L2a1f | KC505118 | 97.4 | N7a1 |
| KJ185427 | 88.4 | L2a5 | HM030548 | 89.2 | N8 |
| JQ701833 | 97.5 | L2b1a3 | AY289059 | 74.4 | O |
| JQ044878 | 99.2 | L2c2b2 | KC993994 | 98.8 | P10 |
| KJ185421 | 98.1 | L2d1a | EF061154 | 78.0 | P3b1 |
| KJ185902 | 95.6 | L2e1a | EF061158 | 92.6 | P4a |
| DQ341081 | 96.5 | L3a1b | AY289064 | 80.3 | P4b |
| JN655803 | 79.0 | L3a+709 | AY289053 | 73.8 | P6 |
| KJ185776 | 97.0 | L3b1a1a | KF451181 | 99.1 | Q1c |
| DQ341074 | 94.0 | L3c | KJ154822 | 95.0 | Q2a |
| KC622102 | 100.0 | L3d3a1a | AY289079 | 85.1 | Q3a |
| JX303776 | 96.0 | L3e1 | JF824990 | 87.0 | R11b |
| EU092895 | 92.5 | L3e3b | AY714045 | 96.6 | R1a1a |
| JN655842 | 90.1 | L3f1a1 | KC911319 | 77.7 | R2 |
| JQ045052 | 92.2 | L3f1b1a | AY963584 | 93.6 | R21 |
| JN655832 | 83.7 | L3f2a1 | GU170818 | 86.7 | R30a1b1 |
| EU092877 | 84.6 | L3f2b | AY714050 | 88.7 | R30b1 |
| AF347000 | 96.3 | L3h1a2a1 | FJ004826 | 98.2 | R31a1 |
| JN655838 | 94.8 | L3h1b1a | AY714046 | 92.8 | R31b |
| JN655820 | 97.7 | L3h2 | FJ004811 | 99.0 | R7b1a1 |
| JN655789 | 95.0 | L3k1 | JF742196 | 90.7 | R8a1a1a2 |
| JN655802 | 88.7 | L3x2a | DQ404441 | 89.4 | S1a |
| FJ460531 | 95.8 | L4a1 | AY289067 | 96.6 | S3 |
| JQ044848 | 93.6 | L4b1a | JQ705673 | 94.5 | T2e |
| EU092951 | 96.9 | L4b2a2b | KC911502 | 90.2 | U1a1a |
| EF556173 | 97.8 | L5a1a | JQ705704 | 92.5 | U1b1 |
| KC911364 | 94.5 | L5b1a | KC533515 | 96.4 | U2a2 |
| EU092802 | 93.8 | L6a | KF450851 | 95.4 | U2b2 |
| FJ770941 | 82.5 | M | JX984460 | 83.0 | U2c1 |
| KF451676 | 92.5 | M10a1+16129 | KC990647 | 62.3 | U2c1 |
| KC709481 | 92.8 | M11c | JQ706067 | 92.4 | U2d2 |
| KJ446520 | 96.2 | M12a1a2 | KJ445816 | 91.0 | U2e1h |
| KF451769 | 81.6 | M13 | JX153094 | 87.3 | U3a2 |
| FJ544230 | 95.6 | M13a2 | JQ704121 | 96.7 | U4c1a |
| JX289092 | 90.0 | M13c | GU296627 | 95.0 | U5b2b1a2 |
| EF495222 | 77.7 | M14 | KC152579 | 91.4 | U6a5 |
| GU810076 | 92.3 | M17a | KC911508 | 92.3 | U7a3 |
| DQ779925 | 93.8 | M1a3b | JX273294 | 85.5 | U8b1a2 |
| HM030505 | 96.9 | M20 | KC911536 | 83.4 | U8b1a2 |
| GQ119046 | 84.2 | M21a | AY339492 | 96.6 | W1a |
| JF739541 | 87.9 | M21b1a | JN415482 | 90.1 | X2b+226 |
| JX289109 | 94.9 | M21b2 | | | |

Table S2: Mitochondrial sources of contamination provided with the software (cont.).

| sample ID | origin | total fragments | total bases | coverage |
|---|---|---|---|---|
| B9687 | Mezmaiskaya | 162,035 | 11,773,544 | 710.577 |
| B9688 | Mezmaiskaya | 148,817 | 10,533,824 | 635.755 |

Table S3: Number of fragments, sum of all bases and coverage for our datasets from empirical samples

| sample | endogenous | | contaminant | | contamination | |
|---|---|---|---|---|---|---|
| | fragments | base pairs | fragments | base pairs | rate per fragment | rate per base |
| B9687 | 30,876 | 2,443,418 | 23,598 | 1,989,785 | 0.433 | 0.449 |
| B9688 | 25,437 | 1,971,127 | 24,083 | 1,972,954 | 0.486 | 0.500 |

Table S4: Tally of the fragments that support diagnostic positions in the archaic humans and *ad hoc* contamination estimate.

| type of sample | Genbank accession |
|---|---|
| Revised Cambridge Reference Sequence (rCRS) | NC_012920 |
| present-day human | AF347008 |
| present-day human | AY195788 |
| present-day human | AF347015 |
| present-day human | AF347014 |
| present-day human | AY289070 |
| present-day human | AF381982 |
| present-day human | AY195773 |
| present-day human | AY195779 |
| present-day human | AY882391 |
| present-day human | AY882415 |
| present-day human | AY882404 |
| present-day human | AF346963 |
| present-day human | AY882386 |
| present-day human | AY289093 |
| present-day human | AF347007 |
| present-day human | AY289095 |
| present-day human | AY289060 |
| present-day human | AY195752 |
| present-day human | AY882417 |
| present-day human | AY195789 |
| Denisovan phalanx | NC_013993 |
| Sima de los Huesos | NC_023100 |
| Neanderthal Mezmaiskaya1 | FM865411 |
| Neanderthal Feldhofer1 | FM865407 |
| Neanderthal Feldhofer2 | FM865408 |
| Neanderthal Vindija33.25 | FM865410 |
| Neanderthal Vindija33.16 | AM948965 |
| Neanderthal Sidron | FM865409 |
| Neanderthal Altai | KC879692 |
| Pan paniscus | NC_001644 |

Table S5: Description of samples used in the maximum likelihood tree with accession identifier

## S2 Additional file 1: Results

### S2.1 Mitochondrial mapping strategies

To show the gains of rewrapping the fragments around the junction section of the mitochondrial genome, a set of 1M fragments from the human reference were simulated. The fragments were taken from the human reference using the same strategy described in the methods section and using the length distribution of the contaminant sequences for the biological data seen in Figure S3. Mapping was performed with BWA v0.5.10 with increased sensitivity ("-n 0.01 -o 2 -l 16500") against the rCRS human reference and against the extended one with the first 1000 basepairs copied at the end where fragments exceeding the length of the reference were wrapped around to span the break point of the reference genome sequence. Figure S8 shows the coverage for the first and last bases of the mitochondrial reference. The advantage of accounting for circularity in mapping is seen by the more even coverage, compared to the alignment to the standard reference genome.

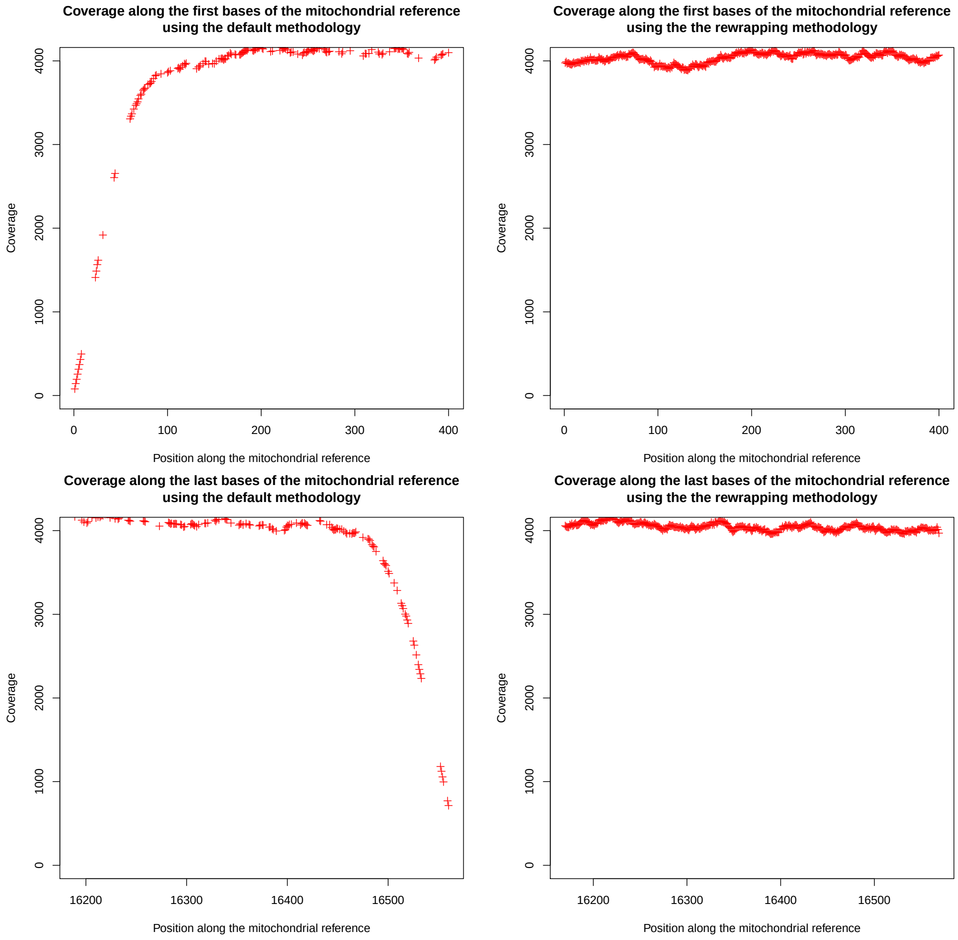


Figure S8: Coverage for the first 400 bases of the mitochondrial genome (top) and last 400 bases (bottom) for simulated short fragments from the rCRS reference. Without accounting for circularity (left) an artificial drop of coverage can be seen. However, if circularity is taken into account (right), the end of the sequence in the reference file does not influence coverage.

As mentioned in the methods section, fragments from the Denisovan mito-

chondrial genome were simulated. Its divergence against the human genome was plotted (see Figure S9). The regions of the mitochondrial genome with the highest divergence can be found around the D-loop. Figure S10 shows the correlation between divergence and coverage. When using BWA, even with parameters tailored for aDNA, a lesser number of fragments align to highly divergent loci. SHRIMP, a more sensitive aligner (see [21]) seems more robust to highly divergent loci. To avoid coverage biases between endogenous and exogenous material, a sensitive aligner is required to accurately quantify contamination.

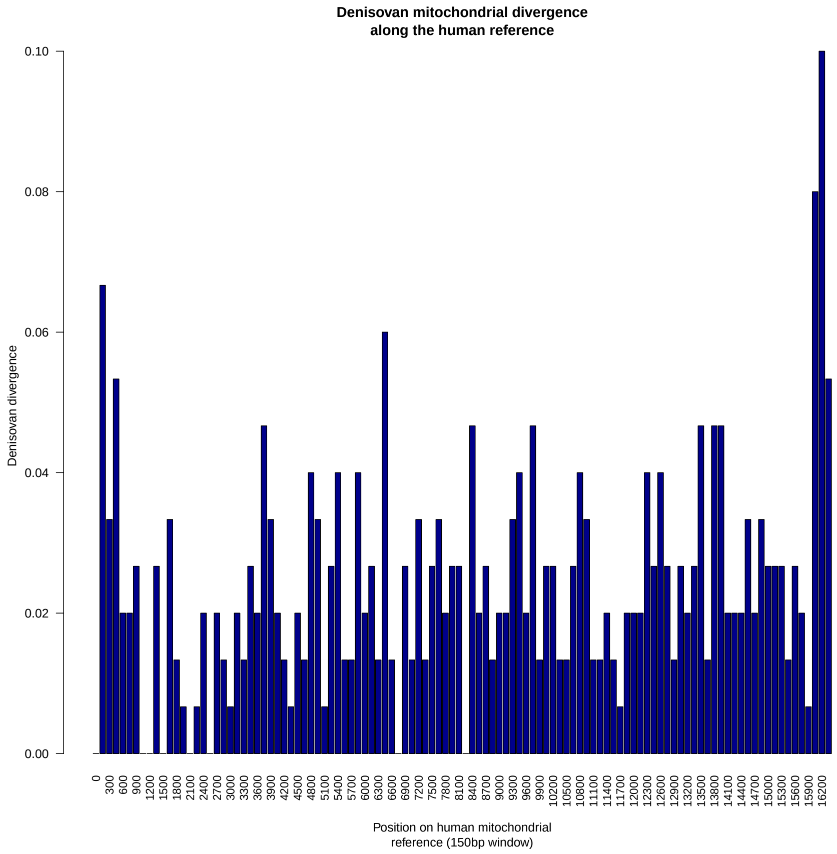


Figure S9: Divergence of the Denisovan mitochondrial genome when aligned to the human reference for windows of 150 basepairs. The most divergent portion of the genome are found in the vicinity of the D-loop.

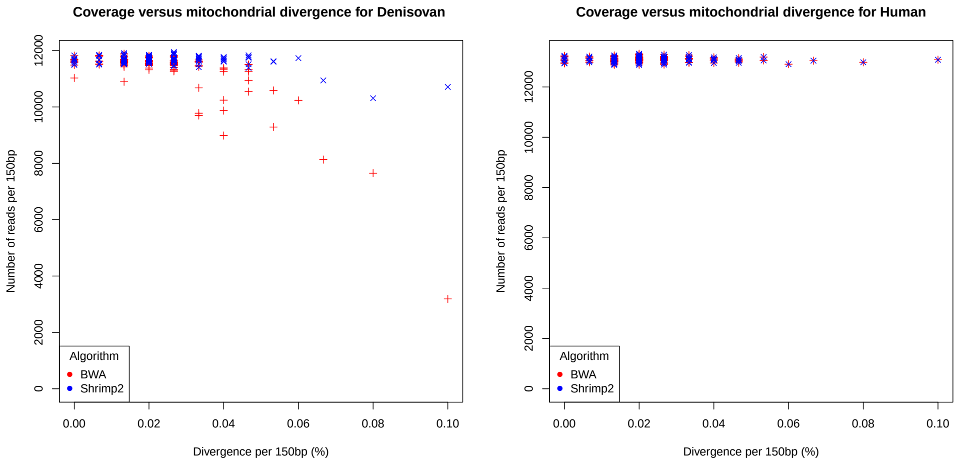


Figure S10: Effect of Denisovan mitochondrial divergence on coverage depending on the aligner. Certain mitochondrial loci of the Denisovan mitochondrial genome are highly divergent to the human reference. The coverage per region is presented both for simulated endogenous fragments from the Denisovan (left) and contaminant fragments (right). BWA (red) performs well at low divergence. At high levels of divergence, the fraction of the contaminant and endogenous fragments that align will not follow the average over the entire genome thus potentially leading to overestimates of contamination rates. SHRIMP (blue) has greater sensitivity to higher divergence and therefore this effect is less prominent.

## S2.2 Empirical data

### S2.2.1 Contamination estimate based on deamination

For the Mezmaiskaya datasets, the maximum *a posteriori* estimates for contamination based on deamination alone were found at 51.0±0.5% and 44.5±0.5% for the B9687 and B9688 samples respectively. The posterior probability distribution was plotted for both samples (see Figure S11). In both cases, the true contamination rate is unknown but both estimates fall within a few percent of the ones presented in Table S4 that were measured using diagnostic positions, thus providing a reasonable initial estimate.

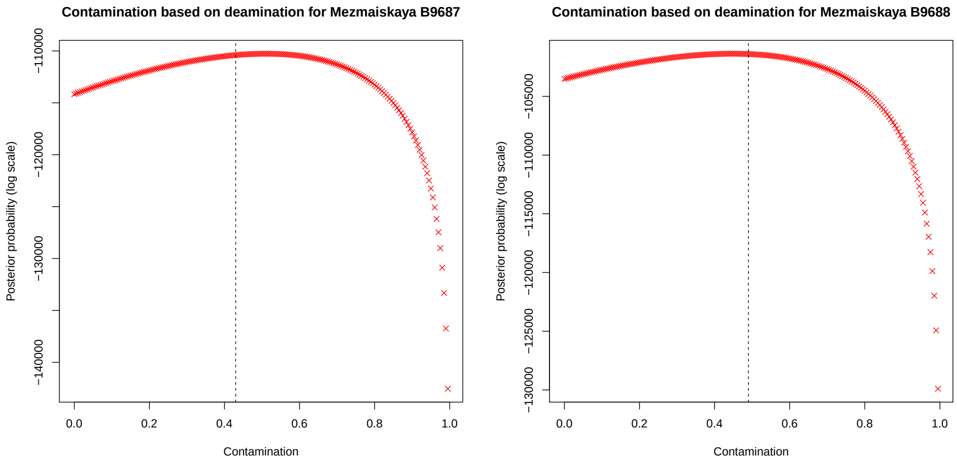


Figure S11: Distribution of the posterior probability for contamination rates as measured by endogenous deamination rates. For our two Mezmaiskaya samples B9687 (left) and B9688 (right), the fraction of contaminant fragments over the total sum is also represented (dotted line).

### S2.2.2 Contamination estimate based on divergent bases

For both Mezmaiskaya datasets, we obtained a contamination rate of 43.0±1.0 and 48.0±1.0 using schmutzi without the inclusion of the predicted contaminant. In both cases, the contamination estimate increased by exactly 1% if the predicted contaminant was used in the database of contaminants (option "–usepredC", see section S1.6). These estimates are closer to the expected ones presented in Table S4 and fall within the lower and upper bounds. The posterior probability distribution shows the peak estimate close to the one obtained using the diagnostic positions (see Figure S12).

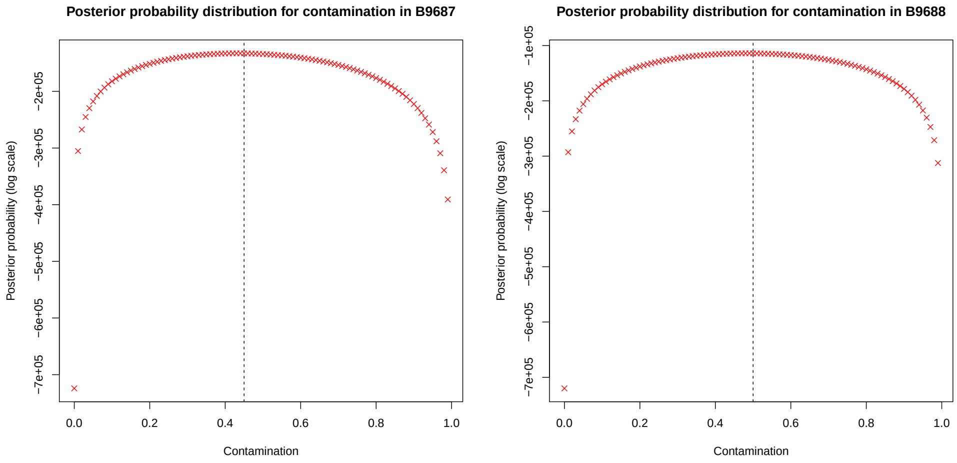


Figure S12: Distribution of the posterior probability for contamination as measured by the endogenous genome and the database of putative contaminants. For the two Mezmaiskaya samples B9687 (left) and B9688 (right), the fraction of contaminant bases over the total sum is also represented (dotted line).

### S2.2.3 Endogenous mitochondrion consensus call

To verify whether the inferred endogenous and contaminant genomes would respectively fall within the predicted archaic and human clades, a maximum likeli-

hood tree was constructed using the mitochondrial genomes from 20 present-day humans and nine archaic hominins enumerated in Section S1 (see Figure S13). The Mezmaiskaya B9687 and B9688 samples cluster with the Mezmaiskaya genome. The contaminant genomes all fall within human variation except the Mezmaiskaya B9687 without any quality filters applied where the contaminant mitochondrion falls outside of all human variations. This is due to low quality bases as a reiteration the phylogenetic reconstruction using only high quality bases resulted in an inferred contaminant mitochondrion which falls within the variation of extant humans. Furthermore, the likelihood of the tree increases as only high quality bases are retained. Attempts to assign the inferred contaminants to known haplogroups are presented in section S2.2.4.

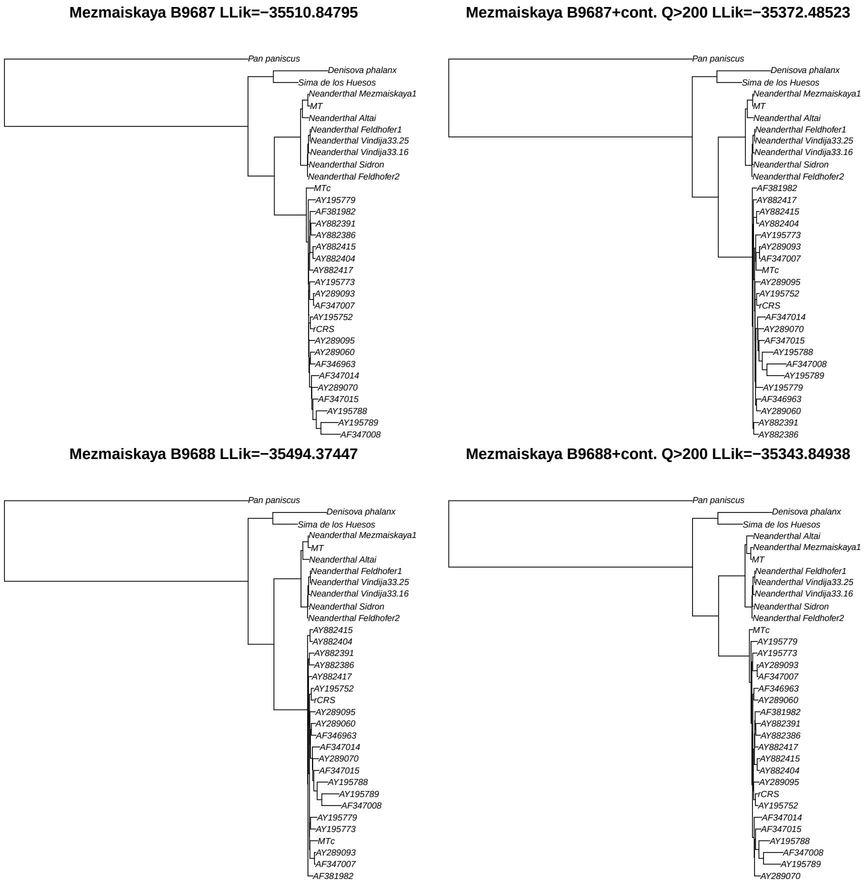


Figure S13: Maximum likelihood trees for the Mezmaiskaya B9687 (top) and B9688 (bottom). The unfiltered data (left) and bases with quality greater than 200 on the PHRED scale (right) were plotted separately. The outgroup used is the bonobo mitochondrial genome.

As the Mezmaiskaya mitochondrial genome had been previously sequenced using data with low present-day human contamination, the endogenous consensus call for both Mezmaiskaya datasets could be compared to this mitochondrial genome. The results for the Mezmaiskaya B9687 sample are described in the main text. Here we describe the quality of the endogenous call we obtained for

Mezmaiskaya B9688.

An alignment of the unfiltered predicted Mezmaiskaya B9688 genome to the original mitochondrion from the same individual revealed a total of ten mismatches, two of which had very low quality score (5.09715 and 83.9567 respectively) while the eight remaining mismatches were all concentrated in a range of 60 basepairs at the end of the mitochondrial reference (positions 16129-16190 on the mitochondrial reference). Using a filter for high quality bases (Q$\geq$200) eliminated the first two miscalls in that loci but left six mismatches in the aforementioned locus of 60 bases on the mitochondrial genome. A closer look revealed a high level of divergence of the Mezmaiskaya mitochondrial genome to the human reference and a drop of coverage in that area. At position 16,139 on the rCRS, for instance, total coverage was 431X and where the contaminant base had 327X coverage thus 75.9% of the fragments. In contrast, the genome-wide mean coverage was 636X and the contamination rate was 48-50% . To verify whether this was due to a bias caused by the short-read aligner, we re-aligned the fragments to the Mezmaiskaya mitochondrial genome. Our results (data not shown) revealed the same drop in coverage in the same area. Communication with the authors involved in generating the original data revealed that, like Mezmaiskaya 1, a mitochondrial capture was performed using a tiled array only with the human base on the probes. Therefore, this artifact was likely due to capture bias which is currently not modeled.

We sought to verify whether schmutzi could call the endogenous mitochondrial genome for samples with low amounts of contamination as well. We ran schmutzi on subset of fragments from the Ust'-Ishim genome [8] (avg. coverage = 124X), the Altai Neanderthal [14] (avg. coverage = 1076X) and the Denisovan individual [22] (avg. coverage = 258X) and compared it to the published reference. In all cases, our prediction was identical to the published reference except for the Denisovan genome where there was an overprediction of one low quality cytosine in a large 6 basepairs insert adjacent to the poly-cytosine strectch (position 5894-5899 on the rCRS).

#### S2.2.4 Contaminant mitochondrion consensus call

As previously mentioned, since there are no tools to call the contaminant mitochondrial genome and since the contaminant was not previously characterized, our inferred contaminant genomes could not be compared to a known sequence. However, as there is a finite set of mitochondrial haplotypes among present-day humans, the predicted contaminant sequence can be compared to existing haplotypes to determine whether it falls within a given haplogroup (i.e. the diagnostic positions for this haplogroup are found).

We present the most likely haplogroup as determined by haplogrep [23, 24] and the calls produced by schmutzi at the diagnostic positions for the most likely haplogroup.

For both Mezmaiskaya samples, the most likely haplogroup as determined by haplogrep was T2b3, a haplogroup predominantly found in Eurasia [25]. All but one of the 33 diagnostic positions were found in the predicted contaminant for the B9687 sample (see Table S6). The single mismatch had low quality relative to the other diagnostic positions. The other Mezmaiskaya sample B9688 had no mismatches for all of the 33 diagnostic positions (see Table S7).

| position | reference base | diagnostic base | predicted base with max. likelihood | quality on a PHRED scale | predicted is equal to diagnostic ? |
|---|---|---|---|---|---|
| 73 | A | G | G | 1126.34 | yes |
| 151 | C | T | T | 206.893 | yes |
| 263 | A | G | G | 664.649 | yes |
| 709 | G | A | A | 1073.22 | yes |
| 750 | A | G | G | 1293.59 | yes |
| 930 | G | A | A | 309.252 | yes |
| 1438 | A | G | G | 994.094 | yes |
| 1888 | G | A | A | 284.227 | yes |
| 2706 | A | G | G | 244.6 | yes |
| 4216 | T | C | C | 96.8476 | yes |
| 4769 | A | G | G | 709.473 | yes |
| 4917 | A | G | G | 252.591 | yes |
| 5147 | G | A | A | 241.557 | yes |
| 7028 | C | T | T | 1151.05 | yes |
| 8697 | G | A | A | 40.3619 | yes |
| 8860 | A | G | G | 1082.47 | yes |
| 10463 | T | C | T | 65.6473 | no |
| 10750 | A | G | G | 899.204 | yes |
| 11251 | A | G | G | 411.396 | yes |
| 11719 | G | A | A | 1096.27 | yes |
| 11812 | A | G | G | 305.569 | yes |
| 13368 | G | A | A | 412.61 | yes |
| 14233 | A | G | G | 232.08 | yes |
| 14766 | C | T | T | 935.827 | yes |
| 14905 | G | A | A | 396.984 | yes |
| 15326 | A | G | G | 1146.32 | yes |
| 15452 | C | A | A | 308.629 | yes |
| 15607 | A | G | G | 297.883 | yes |
| 15928 | G | A | A | 80.2745 | yes |
| 16126 | T | C | C | 28.9019 | yes |
| 16294 | C | T | T | 226.685 | yes |
| 16296 | C | T | T | 212.763 | yes |
| 16304 | T | C | C | 210.421 | yes |

Table S6: Predicted contaminant from the Mezmaiskaya sample B9687 with the diagnostic positions for the T2b3 haplogroup. The base quality reported is from the output of schmutzi and is on a PHRED scale.

| position | reference base | diagnostic base | predicted base with max. likelihood | quality on a PHRED scale | predicted is equal to diagnostic ? |
|---|---|---|---|---|---|
| 73 | A | G | G | 1085.01 | yes |
| 151 | C | T | T | 122.804 | yes |
| 263 | A | G | G | 892.348 | yes |
| 709 | G | A | A | 1216.99 | yes |
| 750 | A | G | G | 1490.97 | yes |
| 930 | G | A | A | 231.495 | yes |
| 1438 | A | G | G | 1173.56 | yes |
| 1888 | G | A | A | 201.053 | yes |
| 2706 | A | G | G | 232.934 | yes |
| 4216 | T | C | C | 71.3592 | yes |
| 4769 | A | G | G | 957.917 | yes |
| 4917 | A | G | G | 264.455 | yes |
| 5147 | G | A | A | 173.945 | yes |
| 7028 | C | T | T | 1407.42 | yes |
| 8697 | G | A | A | 75.387 | yes |
| 8860 | A | G | G | 1127.82 | yes |
| 10463 | T | C | C | 25.2927 | yes |
| 10750 | A | G | G | 968.847 | yes |
| 11251 | A | G | G | 202.335 | yes |
| 11719 | G | A | A | 1444.12 | yes |
| 11812 | A | G | G | 165.341 | yes |
| 13368 | G | A | A | 179.121 | yes |
| 14233 | A | G | G | 263.217 | yes |
| 14766 | C | T | T | 1117.02 | yes |
| 14905 | G | A | A | 312.261 | yes |
| 15326 | A | G | G | 1409.39 | yes |
| 15452 | C | A | A | 146.847 | yes |
| 15607 | A | G | G | 293.051 | yes |
| 15928 | G | A | A | 236.537 | yes |
| 16126 | T | C | C | 143.69 | yes |
| 16294 | C | T | T | 108.801 | yes |
| 16296 | C | T | T | 133.758 | yes |
| 16304 | T | C | C | 135.182 | yes |

Table S7: Predicted contaminant from the Mezmaiskaya sample B9688 with the diagnostic positions for the T2b3 haplogroup. The base quality reported is from the output of schmutzi and is on a PHRED scale.

### S2.2.5 Contamination estimate for 4 ancient mtDNA studies from different laboratories

We evaluated how schmutzi would perform on various publicly available aDNA datasets. Sequencing data from several early modern humans from Haak *et al.* [26] [4], an early modern human from Kostenki (Seguin-Orlando *et al.* [27]) [5], a contaminated Neanderthal from Okladnikov (Skoglund *et al.* [28]) [6] and an early modern human with higher coverage from Mal'ta (Raghavan *et al.* [29]) [7] were downloaded. The aDNA fragments were aligned to the mitochondrial rCRS reference using SHRIMP v2.2.3 (with the same parameters described in section S1.1.2). Our tool was used on all samples but we only report here the results for the 15 largest datasets from the 69 samples from the Haak *et al.* study.

In addition to schmutzi, we also ran contamMix version 1.0-10, previously described in [7] and [8], to provide a comparison to an existing method in terms of contamination estimates and runtime. We ran schmutzi's normal iterative approach. To make sure that the potentially different endogenous consensus call does not influence the contamination estimates, we also ran "mtCont" alone using the same endogenous consensus sequence as the one provided to contamMix. We also compared the runtime of the two programs. In addition, we compared the estimates returned by both schmutzi and contamMix to the ones reported in the original publications which were assessed using a third method. The original publication for Raghavan *et al.* used private mutations and diagnostic positions were used for the Skoglund *et al.* study. This provided a third party estimate for two out of the four studies. Three out of the four studies presented data from modern humans and diagnostic positions for archaic hominins (Neanderthals/Denisovans) are not applicable for modern humans (see section S1.5).

Both schmutzi's mtCont and contamMix were run on a server using AMD Opteron(tm) Processor 2.8GHz CPUS and by limiting the use to 3 cores. When testing mtcont alone, to make sure that solely the contamination estimate was being tested independently of schmutzi's ability to jointly infer the endogenous mitochondrial sequence, the same endogenous mitochondrial sequence and aligned fragments was provided to both programs. In the case of Kostenki and Okladnikov, the reference from NCBI's GenBank was used (GenBank ID FN600416.1 and KF982693.1 respectively). For the remaining two studies, a consensus was generated by using "samtools mpileup" with a minimal coverage of 10 bases and a consensus of 80%, similarly to the approach described by Raghavan *et al.* [29].

Results show that schmutzi produces estimates where the 95% confidence intervals overlap with those from contamMix for the Kostenki and Haak *et al.* studies (see Table S8). As there were no contamination estimates computed using a third method, we can only state that they are generally in agreement. However, to obtain the estimates, schmutzi runs 3 times faster than contamMix.

For the Kostenki sample, the iterative approach to infer the endogenous genome simply from the BAM files was not possible given the low sequence coverage (fewer than 4k aDNA fragments per library). For the Okladnikov data, schmutzi's contamination estimate is closer than contamMix's, to the estimate reported by the authors which was computed using diagnostic positions.

We note that the sequence fragments published for the Mal'ta specimen have extremely low levels deamination (<4% at the ends of the fragments). This is much lower than expected for a sample of this age, and leads to an incorrect

[4] Data obtained from ftp.sra.ebi.ac.uk/vol1/ERA412/ERA412803

[5] Data obtained from http://ricco.popgen.dk/thorfinn/kos18octBlue.bam

[6] Data obtained from the authors

[7] Data obtained from the authors

estimation of the contamination (60%) in the initial iteration of contDeam. In subsequent iterations this estimate is reduced (60% $\rightarrow$ 53% $\rightarrow$ 36% $\rightarrow$ 12% $\rightarrow$ 5%) (Table S8). However, the final estimate remains an apparent over-estimate compared the private site-based approach used by Raghavan et al (cite). A more detailed analysis of the effects of (unexpectedly) low deamination rates on estimation of the contamination prior are presented in section S2.3.4.

We also compared the endogenous consensus call to the publicly available mitochondrial references for Kostenki (GenBank ID: FN600416.1) and Okladnikov (GenBank ID: KF982693.1). For the Okladnikov sample, we only detect a single difference between the published reference and our endogenous consensus call. At position 514 on the rCRS, the published reference shows the deletion of two bases while schmutzi predicts the deletion of 4 bases. A manual inspection of the alignments in IGV [30] showed that schmutzi's prediction is likely correct.

For the various Kostenki datasets, coverage was uneven and very sparse across the mitochondrial genome. This initially lead to the endogenous predictions for the different sets showing a large number of differences to the published genome (246-326). However, no divergent base was found when a threshold of 35 (PHRED scale) on the final prediction score produced by our software thus indicating that those were due to poor coverage.

| Study | Sample | runtime (wallclock) | | contamination estimates (% (95% CI)) | | | third method contamination estimate (% (95% CI)) |
|---|---|---|---|---|---|---|---|
| | | contamMix | schmutzi | contamMix | schmutzi $^{s}$ | schmutzi $^{i}$ | |
| Raghavan *et al.* 2013 | Mal'ta | 94m43s | 44m2s | 7.1 ( 5.6 - 9.1 ) | 3.2 ( 2.9 - 3.5 ) | 5.4 (5.1-5.6) | 1.1 ( 0.0-3.2) |
| Seguin-Orlando *et al.* 2014 | Kostenki_S2_1_37 | 30m42s | 9m26s | 0.1 ( 0.0 - 1.9 ) | 2.8 ( 2.3 - 3.3 ) | N/A | N/A |
| | Kostenski_K14_A1_S2_1 | 24m39s | 10m29s | 1.5 ( 0.5 - 4.0 ) | 3.6 ( 2.9 - 4.3 ) | N/A | N/A |
| | K14_S5_2_C_15 | 26m38s | 7m55s | 2.0 ( 0.9 - 4.6 ) | 3.6 ( 3.0 - 4.2 ) | N/A | N/A |
| | Kostenski_S2_1_36 | 21m28s | 9m35s | 3.8 ( 1.7 - 7.5 ) | 3.7 ( 3.0 - 4.4 ) | N/A | N/A |
| | K14_S5_2_C | 17m29s | 6m53s | 5.0 ( 2.5 - 9.9 ) | 4.2 ( 3.4 - 5.0 ) | N/A | N/A |
| Skoglund *et al.* 2014 | Okladnikov | 41m1s | 16m27s | 8.4 ( 7.2 - 9.7 ) | 9.2 ( 8.6 - 9.8 ) | 9.6 ( 9.0 - 10.2 ) | 10.2 (8.7-11.7) |
| Haak *et al.* 2015 | I0112 | 313m05s | 66m09s | 0.2 ( 0.0 - 0.5 ) | 0.4 ( 0.3 - 0.5 ) | 0.5 ( 0.4 - 0.6 ) | N/A |
| | I0411 | 290m28s | 73m30s | 0.0 ( 0.0 - 0.1 ) | 0.1 ( 0.0 - 0.2 ) | 0.1 ( 0.0 - 0.2 ) | N/A |
| | I0231 | 272m58s | 93m08s | 0.5 ( 0.3 - 0.9 ) | 0.8 ( 0.7 - 0.9 ) | 0.9 ( 0.8 - 1.0 ) | N/A |
| | I0413 | 214m09s | 78m45s | 0.0 ( 0.0 - 0.4 ) | 0.2 ( 0.1 - 0.3 ) | 0.2 ( 0.1 - 0.3 ) | N/A |
| | I0049 | 277m45s | 62m00s | 0.0 ( 0.0 - 0.2 ) | 0.1 ( 0.0 - 0.2 ) | 0.1 ( 0.0 - 0.2 ) | N/A |
| | I0054 | 232m03s | 53m14s | 1.8 ( 1.4 - 2.5 ) | 1.9 ( 1.7 - 2.1 ) | 2.5 ( 2.3 - 2.7 ) | N/A |
| | I0443 | 277m54s | 54m19s | 0.1 ( 0.0 - 0.4 ) | 0.5 ( 0.4 - 0.6 ) | 0.6 ( 0.5 - 0.7 ) | N/A |
| | I0099 | 280m49s | 48m48s | 0.2 ( 0.1 - 0.7 ) | 0.7 ( 0.6 - 0.8 ) | 0.7 ( 0.6 - 0.8 ) | N/A |
| | I0409 | 133m03s | 70m04s | 0.0 ( 0.0 - 0.2 ) | 0.1 ( 0.0 - 0.2 ) | 0.1 ( 0.0 - 0.2 ) | N/A |
| | I0172 | 171m16s | 51m52s | 1.5 ( 1.0 - 2.2 ) | 2.6 ( 2.4 - 2.8 ) | 4.9 ( 4.6 - 5.2 ) | N/A |
| | I0104 | 182m27s | 66m35s | 0.1 ( 0.0 - 0.3 ) | 0.8 ( 0.7 - 0.9 ) | 0.8 ( 0.7 - 0.9 ) | N/A |
| | I0060 | 248m54s | 68m19s | 0.0 ( 0.0 - 0.2 ) | 0.1 ( 0.0 - 0.2 ) | 0.5 ( 0.4 - 0.6 ) | N/A |
| | I0118 | 136m41s | 46m03s | 1.6 ( 0.9 - 3.0 ) | 1.9 ( 1.7 - 2.1 ) | 2.6 ( 2.3 - 2.9 ) | N/A |
| | I0100 | 172m15s | 46m28s | 0.9 ( 0.5 - 1.5 ) | 1.2 ( 1.0 - 1.4 ) | 1.3 ( 1.1 - 1.5 ) | N/A |
| | I0412 | 119m52s | 9m46s | 0.2 ( 0.0 - 0.8 ) | 1.4 ( 1.2 - 1.6 ) | 2.2 ( 1.9 - 2.5 ) | N/A |

Table S8: Comparison of contamination estimates obtained using schmutzi's "mtCont" and "contamMix", an implementation from the authors of a previously published contamination method, for various aDNA datasets. Runtimes are also reported. The $^{s}$ shows where the same endogenous consensus was provided to both schmutzi's "mtCont" and contamMix, while $^{i}$ shows the contamination

## S2.3 Simulated data

For the simulated data, the accuracy of the consensus call for both the endogenous and the contaminant genomes was evaluated as the original mitochondrial genomes were known. We also measured the correlation between the simulated contamination rate and the one calculated by schmutzi.

### S2.3.1 Endogenous mitochondrion consensus call

As detailed in the methods, fragments from three endogenous mitochondrial genomes (Denisovan, Neanderthal and early modern human (EMH)) were each blended independently with the fragments of a contaminant individual at various rates. Two sets were created, one where the endogenous fragments had a damage pattern consistent with a double-stranded library and the other set with a single-stranded library. For the former, the endogenous consensus was also computed using PMDtools and htslib to provide a comparison. We also compared the approach to infer the endogenous consensus of keeping fragments with signs of deamination, masking the deaminated bases and calling a consensus using "samtools mpileup". For each set, schmutzi was run using default parameters and the edit distance of the predicted endogenous genome to its respective reference was computed. Furthermore, we computed the edit distance of the predicted contaminant to the original contaminant mitochondrion. We also ran schmutzi using the multiple contaminant option (described in section S1.3) and compared its predicted endogenous genome. All the data presented in the remaining tables were computed without using any filters on the resulting predictions as to accurately represent error rates. Practically speaking, we encourage users to retain only high quality predictions for downstream analyses.

As our algorithm relies on computing the length and deamination patterns of the endogenous and contaminant fragments, a paucity of contaminant fragments at low contamination rates can result in the program stopping after the first iteration. In subsequent tables, a † symbol on a data point indicates that the algorithm did not continue on to the second iteration due to a lack of detected contaminant at low simulated contamination rates, thus the results presented are the ones from the first iteration. At high contamination rates, as the prediction of the endogenous genome becomes more arduous, the endogenous consensus genome will contain more bases from the contaminant, thus leading to an underestimation of contamination, which can in turn lead to the algorithm not converging. As we mention in the software manual, a corrective measure can be performed by using the predicted contaminant genome as a putative contaminant source via the "–usepredC" option. Sets marked with a ‡ indicate that the predicted contaminant was used as a contaminant source. For very hard targets (e.g., EHM with around 90% contamination), the workflow provided by the wrapper script diverges even with the option of using the contaminant source. For such hard targets, manual intervention would be required and data that caused this type of problem is marked with an $*$ in subsequent tables.

For the simulated EMH, the endogenous genome predicted by schmutzi is identical to the simulated data, up to a contamination rate of 35% for the double-stranded data and up to a contamination rate of 40% for single-stranded libraries (see Tables S9 and S10). As single-stranded data had greater rates of deamination, there is more power to accurately predict the contaminant and endogenous bases. As the contamination increases, more indels and mismatches appear. The consensus made on the deaminated fragments using PMDtools and htslib has two indels compared to the endogenous genome, both of which are located in a region of two consecutive insertions in the EMH mitochondrion genome.

For the Neanderthal data, the results for the double-strand protocol are

presented in the main text. The same comparison to the endogenous genome was made using the data simulated under a single-stranded protocol (see Table S12). Our algorithm was able to perfectly predict the endogenous genome up to a contamination of 25% and with a single mismatch up to a contamination rate of 90%. That single mismatch occurred in a region of high divergence of the Neanderthal genome and had a low quality score relative to the neighboring bases. For the Denisovan simulations, we computed the edit distance of the predicted endogenous genome to the Denisovan one (see Table S14 and S13). This dataset had the highest divergence to the human reference. A single recurrent error was present even at low contamination around base 302 on the rCRS due to high divergence creating ambiguous short-read alignments. However, re-running schmutzi's endogenous callers using the predicted endogenous genome as reference successfully removes this single mismatch (data not shown). Despite this, our algorithm was more robust to high contamination than the current approach of isolating deaminated fragments and calling a consensus. For all three types of endogenous genomes, at low levels of contamination (up to 10%), schmutzi did not go forward after the first iteration due to the lack of contaminating fragments. However, in all cases, the endogenous genome called after the first iteration gave an inference of sufficient quality with no or very few mismatches to the original genome.

Our simulations show that the multiple contaminant option works well at very low rates of contamination, but does not at medium or high rates (see Tables S9 through S14).

| Contamination rate | schmutzi Default parameters | schmutzi Multiple Contaminants | PMDtools and htslib |
|---|---|---|---|
| 0.01 | 16570/0/0 † | 16570/0/0 | 16566/1/3 |
| 0.05 | 16570/0/0 † | 16570/0/0 | 16566/1/3 |
| 0.10 | 16570/0/0 † | 16570/0/0 | 16566/1/3 |
| 0.15 | 16570/0/0 | 16570/0/0 | 16566/1/3 |
| 0.20 | 16570/0/0 | 16567/1/2 | 16566/1/3 |
| 0.25 | 16570/0/0 | 16567/1/2 | 16566/1/3 |
| 0.30 | 16570/0/0 | 16567/1/2 | 16566/1/3 |
| 0.35 | 16570/0/1 | 16567/1/3 | 16566/1/3 |
| 0.40 | 16569/1/1 | 16567/1/3 | 16566/1/3 |
| 0.45 | 16569/1/1 | 16547/21/3 | 16566/1/3 |
| 0.50 | 16569/1/1 | 16547/21/3 | 16566/1/3 |
| 0.55 | 16569/1/1 | 16547/21/3 | 16566/1/3 |
| 0.60 | 16570/0/2 | 16547/21/3 | 16566/1/3 |
| 0.65 | 16570/0/2 | 16547/21/3 | 16566/1/3 |
| 0.70 | 16570/0/2 | 16547/21/3 | 16566/1/3 |
| 0.75 | 16569/1/2 ‡ | 16547/21/3 | 16566/1/3 |
| 0.80 | 16569/1/2 ‡ | 16547/21/3 | 16566/1/3 |
| 0.85 | NA/NA/NA * | 16547/21/3 | 16564/3/3 |
| 0.90 | NA/NA/NA * | 16547/21/3 | 16564/3/3 |
| 0.95 | NA/NA/NA * | 16547/21/3 | 16560/7/3 |

Table S9: Edit distance to the original endogenous genome using an early modern human genome and a double-strand protocol. The original endogenous genome had 16547 matches, 21 mismatches and 3 indels to the contaminant.

For calling the endogenous mitochondrial genome consensus, the mapping iterative assembler (MIA) was originally developed for reconstructing the Neanderthal mitochondrial genome [31]. MIA has been used for reconstructing the

| Contamination rate | schmutzi | | mpileup consensus on deaminated fragments |
|---|---|---|---|
| | Default parameters | Multiple Contaminants | |
| 0.01 | 16570/0/1 † | 16570/0/0 | 16567/1/2 |
| 0.05 | 16570/0/0 † | 16570/0/0 | 16567/1/2 |
| 0.10 | 16570/0/0 † | 16570/0/0 | 16567/1/2 |
| 0.15 | 16570/0/0 | 16570/0/0 | 16567/1/2 |
| 0.20 | 16570/0/0 | 16567/1/2 | 16567/1/2 |
| 0.25 | 16570/0/0 | 16567/1/2 | 16567/1/2 |
| 0.30 | 16570/0/0 | 16567/1/2 | 16567/1/2 |
| 0.35 | 16570/0/0 | 16567/1/3 | 16567/1/2 |
| 0.40 | 16569/1/1 | 16567/1/3 | 16567/1/2 |
| 0.45 | 16569/1/1 | 16548/20/3 | 16567/1/2 |
| 0.50 | 16570/0/2 | 16547/21/3 | 16567/1/2 |
| 0.55 | 16569/1/1 | 16547/21/3 | 16567/1/2 |
| 0.60 | 16570/0/2 | 16547/21/3 | 16566/2/2 |
| 0.65 | 16570/0/2 | 16547/21/3 | 16566/2/2 |
| 0.70 | 16570/0/2 | 16547/21/3 | 16562/6/2 |
| 0.75 | 16570/0/2 | 16547/21/3 | 16562/6/2 |
| 0.80 | 16570/0/2 ‡ | 16547/21/3 | 16561/7/2 |
| 0.85 | 16569/1/2 ‡ | 16547/21/3 | 16558/7/5 |
| 0.90 | 16569/1/2 ‡ | 16547/21/3 | 16561/7/2 |
| 0.95 | 16568/2/2 ‡ | 16547/21/3 | 16553/10/7 |

Table S10: Edit distance to the original endogenous genome using an early modern human genome and a single-strand protocol. The original endogenous genome had 16547 matches, 21 mismatches and 3 indels to the contaminant.

mitochondrial genome for multiple samples [32, 7, 3]. The latest version of MIA[8] was used on our simulated datasets and the distance to the original endogenous genome was computed (see table S15). Our results show that present-day human contamination quickly overruns the consensus call. This effect limits the applicability of a straightforward consensus call to samples with low rates of present-day human contamination.

### S2.3.2 Contaminant mitochondrion consensus call

As previously mentioned, no currently available tool enables users to the call the contaminant mitochondrial genome. However, we compared schmutzi's consensus call for the contaminant genome to the original contaminant genome used by computing the edit distance as a metric (see Table S16 and S17). At very low rates of contamination, schmutzi is unable to call the contaminant mitochondrial genome. For contamination rates of about 20% and higher, the prediction of the contaminant genome is nearly perfect regardless of which endogenous genome was used.

**Effect of lower coverage** In section S2.3.3, we describe the effect of subsampling the original BAM file on the contamination estimate for simulated datasets with heavy present-day human contamination. This is done to evaluate the limits of our algorithm in terms of coverage on the most difficult targets. We report here the edit distance to the simulated endogenous Neanderthal genome as a function of coverage for the same difficult targets as presented in Section S2.3.3

[8]URL: https://github.com/udo-stenzel/mapping-iterative-assembler version:5a7fb5afad735da7b8297381648049985c599874

| Contamination rate | schmutzi | | PMDtools and htslib |
|---|---|---|---|
| | Default parameters | Multiple Contaminants | |
| 0.01 | 16565/0/0 | 16565/0/0 | 16561/2/6 |
| 0.05 | 16565/0/0 † | 16565/0/0 | 16561/2/6 |
| 0.10 | 16565/0/0 | 16565/0/0 | 16561/2/6 |
| 0.15 | 16565/0/0 | 16565/0/0 | 16560/3/6 |
| 0.20 | 16565/0/0 | 16564/1/0 | 16560/3/6 |
| 0.25 | 16565/0/0 | 16562/2/1 | 16558/5/6 |
| 0.30 | 16564/1/0 | 16559/5/5 | 16558/5/6 |
| 0.35 | 16564/1/0 | 16550/3/28 | 16556/7/6 |
| 0.40 | 16564/1/0 | 16542/22/6 | 16555/8/6 |
| 0.45 | 16564/1/0 | 16355/209/6 | 16553/10/6 |
| 0.50 | 16563/2/0 | 16355/209/6 | 16553/10/6 |
| 0.55 | 16564/1/0 | 16355/209/6 | 16554/9/6 |
| 0.60 | 16563/2/0 | 16355/209/6 | 16551/12/6 |
| 0.65 | 16563/1/1 | 16355/209/6 | 16551/12/6 |
| 0.70 | 16562/1/2 | 16355/209/6 | 16548/15/6 |
| 0.75 | 16563/1/1 | 16355/209/6 | 16546/17/6 |
| 0.80 | 16561/2/2 ‡ | 16355/209/6 | 16545/18/6 |
| 0.85 | 16563/1/1 ‡ | 16355/209/6 | 16544/19/6 |
| 0.90 | 16561/3/1 ‡ | 16355/209/6 | 16539/24/6 |
| 0.95 | 16550/15/7 ‡ | 16355/209/6 | 16532/31/6 |

Table S11: Edit distance to the original endogenous genome using a Neanderthal genome and a double-strand protocol. The original endogenous genome had 16355 matches, 209 mismatches and 6 indels to the contaminant.

(48% present-day human contamination). We ran schmutzi with the inclusion of the estimate of the fragment length in the computation to insure the highest accuracy in terms of endogenous base prediction.

Our results show that even at 48% contamination, our algorithm is able to call the endogenous genome sequence down to a coverage of about 20X (see Table S18). The few mismatches that are observed can be avoided by filtering on the log of the posterior probability of the endogenous base provided by our program. However, this filtering comes at a cost. For instance, at 15X coverage for this very difficult contamination rate, we lose about 1k bases. As seen in previous sections, the prediction is slightly more accurate for the single-stranded data as higher rates of deamination allows our algorithm to identify the endogenous base with greater precision. It should be noted that for lower rates of contamination, our algorithm can achieve an endogenous prediction even at a lower coverage.

### S2.3.3 Contamination estimate based on deamination

We also sought to measure the correlation of the contamination estimates obtained using endogenous deamination patterns to the simulated ones. This is the contamination estimate provided to the endogenous caller for the first iteration. We measured correlation between simulated and predicted contamination rates for full datasets with 1M fragments. We also measured robustness to low coverage by subsampling the set taken from the set containing 1M fragments with 40% contamination. The target contamination rate for the simulations was calculated as the fraction of fragments pertaining to the contaminant over the total.

| Contamination rate | schmutzi | | mpileup consensus on deaminated fragments |
|---|---|---|---|
| | Default parameters | Multiple Contaminants | |
| 0.01 | 16565/0/0 | 16565/0/0 | 16565/0/0 |
| 0.05 | 16565/0/0 | 16565/0/0 | 16565/0/0 |
| 0.10 | 16565/0/0 | 16565/0/0 | 16565/0/0 |
| 0.15 | 16565/0/0 | 16564/1/0 | 16565/0/0 |
| 0.20 | 16565/0/0 | 16564/1/0 | 16565/0/0 |
| 0.25 | 16565/0/0 | 16562/2/1 | 16565/0/0 |
| 0.30 | 16564/1/0 | 16559/5/5 | 16565/0/0 |
| 0.35 | 16564/1/0 | 16550/3/28 | 16565/0/0 |
| 0.40 | 16564/1/0 | 16549/15/6 | 16565/0/0 |
| 0.45 | 16564/1/0 | 16356/208/6 | 16565/0/0 |
| 0.50 | 16564/1/0 | 16355/209/6 | 16565/0/0 |
| 0.55 | 16564/1/0 | 16355/209/6 | 16564/0/1 |
| 0.60 | 16564/1/0 | 16355/209/6 | 16563/1/1 |
| 0.65 | 16563/1/1 | 16355/209/6 | 16560/4/1 |
| 0.70 | 16564/1/0 | 16355/209/6 | 16556/8/1 |
| 0.75 | 16563/1/1 | 16355/209/6 | 16546/7/23 |
| 0.80 | 16563/1/1 | 16355/209/6 | 16548/15/6 |
| 0.85 | 16564/1/0 | 16355/209/6 | 16544/20/1 |
| 0.90 | 16563/2/0 | 16355/209/6 | 16536/25/4 |
| 0.95 | 16558/7/0 | 16355/209/6 | 16524/33/12 |

Table S12: Edit distance to the original endogenous genome using a Neanderthal genome and a single-strand protocol. The original endogenous genome had 16355 matches, 209 mismatches and 6 indels to the contaminant.

### Full datasets

Our software, schmutzi, was run on our simulated datasets with 1M fragments to estimate contamination based on deamination patterns alone. We ran our software for both categories of sets: one category where the endogenous genome had a double-stranded type of damage pattern, and the other where a single-stranded damage profile was used.

Our results show that, regardless of the simulated DNA library-preparation protocol, our algorithm produces an estimate that is close to the simulated rate (see Figure S14). Furthermore, these estimates are robust to lower or higher divergence of the contaminant genome to the endogenous one, as this relationship is not *a priori* needed for this approach to produce an estimate.

### Subsampled datasets

To evaluate the robustness of our contamination estimate based on deamination patterns to lower coverage, the dataset with 1M fragments and 40% contamination from the previous section was subsampled at various fractions ranging from 0.01 to 0.5. Our algorithm to predict contamination based on deamination patterns was run on those and the correlation to the original contamination rate was plotted (see Figure S15). Our results show that for the contamination estimate to be stable, a minimal mitochondrial coverage of about 100X to 250X is needed, which, depending on the size of the aDNA fragments, represents approximately 50k to 100k mapped fragments. The simulated type of library protocol or the type of endogenous genome used does not seem to affect the prediction.

| Contamination rate | schmutzi Default parameters | schmutzi Multiple Contaminants | PMDtools and htslib |
|---|---|---|---|
| 0.01 | 16569/1/0 | 16569/1/0 | 16557/2/19 |
| 0.05 | 16569/1/0 | 16569/1/1 | 16557/2/19 |
| 0.10 | 16569/1/1 | 16566/2/2 | 16557/2/19 |
| 0.15 | 16569/1/1 | 16566/2/5 | 16557/2/19 |
| 0.20 | 16568/2/0 | 16559/3/11 | 16557/2/19 |
| 0.25 | 16567/3/0 | 16558/4/12 | 16557/2/19 |
| 0.30 | 16567/2/1 | 16554/8/14 | 16557/2/19 |
| 0.35 | 16567/2/1 | 16552/9/17 | 16555/4/19 |
| 0.40 | 16567/3/0 | 16515/46/17 | 16554/5/19 |
| 0.45 | 16567/3/2 | 16174/387/17 | 16554/5/19 |
| 0.50 | 16568/2/0 | 16174/387/17 | 16551/8/19 |
| 0.55 | 16568/2/2 | 16174/387/17 | 16550/9/19 |
| 0.60 | 16566/2/4 | 16174/387/17 | 16549/10/19 |
| 0.65 | 16566/2/4 | 16174/387/17 | 16547/12/19 |
| 0.70 | 16566/2/4 | 16174/387/17 | 16544/15/19 |
| 0.75 | 16566/2/4 | 16174/387/17 | 16541/18/19 |
| 0.80 | 16566/4/2 | 16174/387/17 | 16534/25/19 |
| 0.85 | 16567/3/4 | 16174/387/17 | 16532/27/19 |
| 0.90 | 16565/5/7 ‡ | 16174/387/17 | 16529/31/17 |
| 0.95 | NA/NA/NA * | 16174/387/17 | 16512/48/17 |

Table S13: Edit distance to the original endogenous genome using a Denisovan genome and a double-strand protocol. The original endogenous genome had 16174 matches, 387 mismatches and 17 indels to the contaminant.

| Contamination rate | schmutzi Default parameters | schmutzi Multiple Contaminants | mpileup consensus on deaminated fragments |
|---|---|---|---|
| 0.01 | 16569/1/0 | 16569/1/0 | 16560/1/9 |
| 0.05 | 16569/1/0 | 16569/1/0 | 16560/1/9 |
| 0.10 | 16569/1/0 | 16566/2/2 | 16560/1/9 |
| 0.15 | 16569/1/0 | 16566/2/5 | 16560/1/9 |
| 0.20 | 16567/3/0 | 16559/3/11 | 16560/2/8 |
| 0.25 | 16568/2/0 | 16559/3/14 | 16560/2/8 |
| 0.30 | 16567/3/0 | 16555/7/14 | 16559/3/8 |
| 0.35 | 16567/3/0 | 16552/9/17 | 16560/2/8 |
| 0.40 | 16567/3/0 | 16542/19/17 | 16566/1/3 |
| 0.45 | 16567/3/0 | 16174/387/17 | 16560/1/9 |
| 0.50 | 16567/2/1 | 16174/387/17 | 16560/2/8 |
| 0.55 | 16568/2/0 | 16174/387/17 | 16559/3/8 |
| 0.60 | 16567/3/2 | 16174/387/17 | 16561/1/8 |
| 0.65 | 16567/3/2 | 16174/387/17 | 16560/1/9 |
| 0.70 | 16568/2/2 | 16174/387/17 | 16557/5/8 |
| 0.75 | 16569/1/2 | 16174/387/17 | 16558/9/3 |
| 0.80 | 16568/2/2 | 16174/387/17 | 16549/13/8 |
| 0.85 | 16569/1/4 | 16174/387/17 | 16538/23/12 |
| 0.90 | 16569/1/2 | 16174/387/17 | 16524/37/12 |
| 0.95 | 16563/7/7 | 16174/387/17 | 16505/56/12 |

Table S14: Edit distance to the original endogenous genome using a Denisovan genome and a single-strand protocol. The original endogenous genome had 16174 matches, 387 mismatches and 17 indels to the contaminant.

| Contamination rate | MIA (with -k 12) | | |
|---|---|---|---|
| | EMH | Neanderthal | Denisovan |
| 0.01 | 16570/0/0 | 16523/1/4 | 16560/0/5 |
| 0.05 | 16565/1/3 | 16549/10/4 | 16565/0/6 |
| 0.10 | 16565/1/3 | 16545/10/4 | 16565/0/6 |
| 0.15 | 16565/1/3 | 16528/10/4 | 16548/0/12 |
| 0.20 | 16562/1/3 | 16360/11/4 | 16211/0/14 |
| 0.25 | 16547/1/3 | 16355/13/5 | 16175/2/14 |
| 0.30 | 16547/1/3 | 16355/18/5 | 16175/5/15 |
| 0.35 | 16547/1/3 | 16355/18/6 | 16174/19/17 |
| 0.40 | 16547/1/3 | 16355/20/6 | 16174/27/17 |
| 0.45 | 16547/2/3 | 16355/29/6 | 16174/30/17 |
| 0.50 | 16547/3/3 | 16355/44/6 | 16174/41/17 |
| 0.55 | 16547/3/3 | 16355/58/6 | 16174/65/17 |
| 0.60 | 16547/3/3 | 16355/109/6 | 16174/106/17 |
| 0.65 | 16547/3/3 | 16355/195/6 | 16174/220/17 |
| 0.70 | 16547/16/3 | 16355/209/6 | 16174/386/17 |
| 0.75 | 16547/20/3 | 16355/209/6 | 16174/387/17 |
| 0.80 | 16547/21/3 | 16355/209/6 | 16174/387/17 |
| 0.85 | 16547/20/3 | 16355/209/6 | 16174/387/17 |
| 0.90 | 16547/21/3 | 16355/209/6 | 16174/387/17 |
| 0.95 | 16547/21/3 | 16355/209/6 | 16174/387/17 |

Table S15: Edit distance of the consensus genome predicted using MIA to the original endogenous genome when using a double-strand protocol. The contaminant genome had 16547 matches, 21 mismatches and 3 indels to the early modern human genome, 16355 matches, 209 mismatches and 6 indels to the Neanderthal genome and 16174 matches, 387 mismatches and 17 indels to the Denisova genome.

| Contamination rate | schmutzi with default parameters | | |
|---|---|---|---|
| 0.01 | 16538/20/44 † | 16442/127/40 | 16311/254/150 |
| 0.05 | 16537/21/34 † | 16358/211/62 † | 16563/2/13 |
| 0.10 | 16544/25/11 † | 16567/0/2 | 16566/0/6 |
| 0.15 | 16568/1/2 | 16567/0/2 | 16566/3/2 |
| 0.20 | 16568/1/2 | 16569/0/0 | 16568/0/1 |
| 0.25 | 16568/1/2 | 16569/0/0 | 16569/0/0 |
| 0.30 | 16569/0/1 | 16569/0/0 | 16569/0/0 |
| 0.35 | 16569/0/0 | 16569/0/0 | 16569/0/0 |
| 0.40 | 16569/0/0 | 16569/0/0 | 16569/0/0 |
| 0.45 | 16569/0/0 | 16569/0/0 | 16569/0/0 |
| 0.50 | 16569/0/0 | 16569/0/0 | 16569/0/0 |
| 0.55 | 16569/0/0 | 16569/0/0 | 16569/0/0 |
| 0.60 | 16569/0/0 | 16569/0/0 | 16569/0/0 |
| 0.65 | 16569/0/0 | 16569/0/0 | 16569/0/0 |
| 0.70 | 16569/0/0 | 16569/0/0 | 16569/0/0 |
| 0.75 | 16569/0/0 ‡ | 16569/0/0 | 16569/0/0 |
| 0.80 | 16569/0/0 ‡ | 16569/0/0 ‡ | 16569/0/0 |
| 0.85 | NA/NA/NA * | 16569/0/0 ‡ | 16569/0/0 |
| 0.90 | NA/NA/NA * | 16569/0/0 ‡ | 16569/0/0 ‡ |
| 0.95 | NA/NA/NA * | 16569/0/0 ‡ | NA/NA/NA * |

Table S16: Edit distance of the predicted contaminant genome to the original contaminant genome when using a double-strand protocol. The contaminant genome had 16547 matches, 21 mismatches and 3 indels to the early modern human genome, 16355 matches, 209 mismatches and 6 indels to the Neanderthal genome and 16174 matches, 387 mismatches and 17 indels to the Denisova genome.

| Contamination rate | schmutzi with default parameters | | |
|---|---|---|---|
| 0.01 | 16537/21/35 † | 16435/134/12 | 16196/369/114 |
| 0.05 | 16538/20/35 † | 16567/0/2 | 16565/1/13 |
| 0.10 | 16537/21/35 † | 16567/0/2 | 16566/0/6 |
| 0.15 | 16568/1/2 | 16568/0/1 | 16566/3/2 |
| 0.20 | 16568/1/2 | 16569/0/0 | 16566/3/0 |
| 0.25 | 16568/1/2 | 16569/0/0 | 16569/0/0 |
| 0.30 | 16569/0/0 | 16569/0/0 | 16569/0/0 |
| 0.35 | 16569/0/0 | 16569/0/0 | 16569/0/0 |
| 0.40 | 16569/0/0 | 16569/0/0 | 16569/0/0 |
| 0.45 | 16569/0/0 | 16569/0/0 | 16569/0/0 |
| 0.50 | 16569/0/0 | 16569/0/0 | 16569/0/0 |
| 0.55 | 16569/0/0 | 16569/0/0 | 16569/0/0 |
| 0.60 | 16569/0/0 | 16569/0/0 | 16569/0/0 |
| 0.65 | 16569/0/0 | 16569/0/0 | 16569/0/0 |
| 0.70 | 16569/0/0 | 16569/0/0 | 16569/0/0 |
| 0.75 | 16569/0/0 | 16569/0/0 | 16569/0/0 |
| 0.80 | 16569/0/0 ‡ | 16569/0/0 | 16569/0/0 |
| 0.85 | 16569/0/0 ‡ | 16569/0/0 | 16569/0/0 |
| 0.90 | 16569/0/0 ‡ | 16569/0/0 | 16569/0/0 |
| 0.95 | 16569/0/0 ‡ | 16569/0/0 | 16569/0/0 |

Table S17: Edit distance of the predicted contaminant genome to the original contaminant genome when using a single-strand protocol. The contaminant genome had 16547 matches, 21 mismatches and 3 indels to the early modern human genome, 16355 matches, 209 mismatches and 6 indels to the Neanderthal genome and 16174 matches, 387 mismatches and 17 indels to the Denisova genome.

| average coverage | double-stranded mismatches (q20) | single-stranded mismatches (q20) |
|---|---|---|
| 15.2 | 13 (15430/0) | 17 (15529/0) |
| 20.5 | 8 (16225/1) | 6 (16347/0) |
| 24.9 | 6 (16509/0) | 3 (16489/0) |

Table S18: Effect of coverage on accuracy of the endogenous consensus call for simulated data. The number in parentheses represent the number of matches and mismatches to the original endogenous mitochondrial genome.

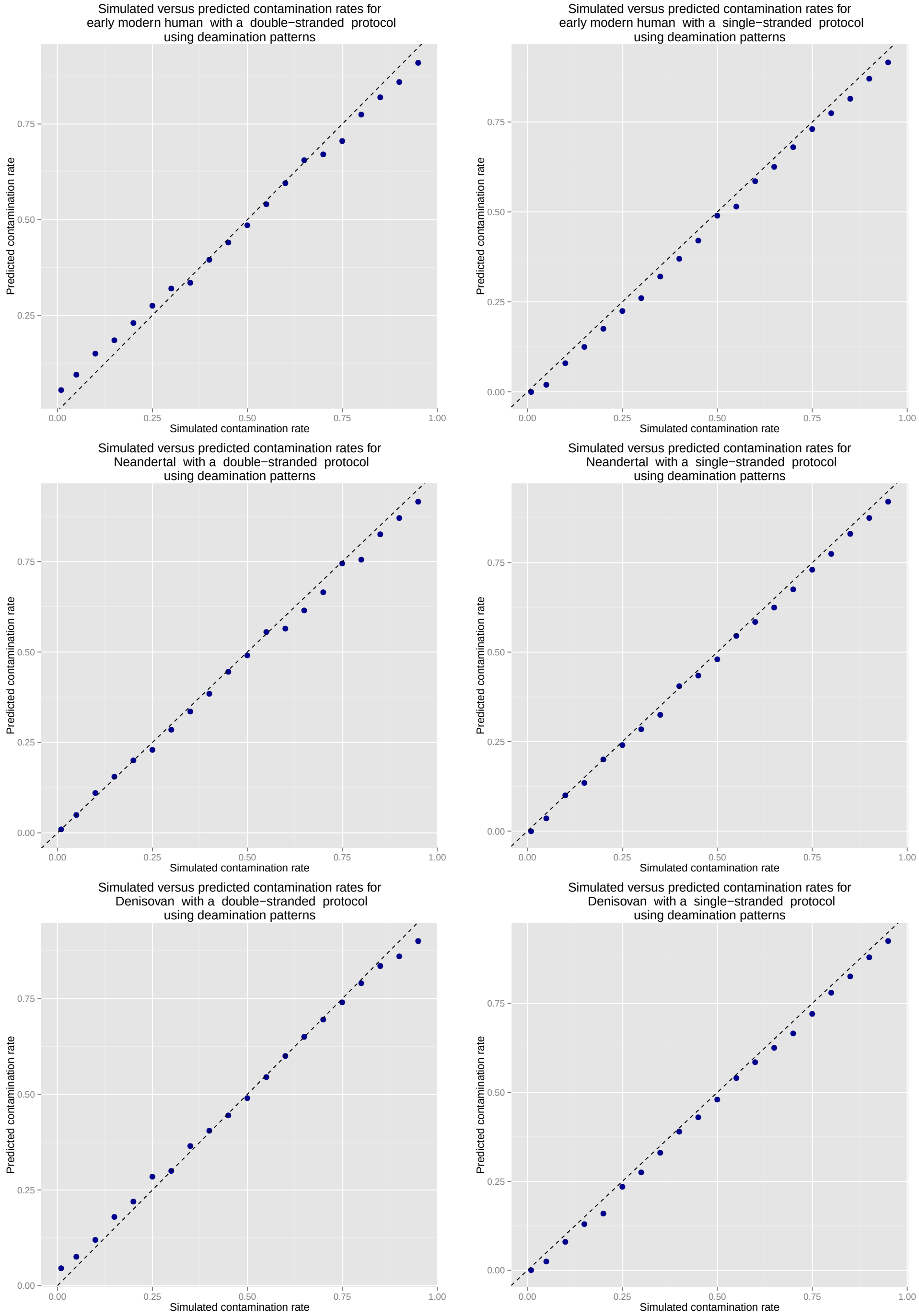


Figure S14: Simulated contamination rates versus predicted contamination ones using deamination patterns alone. Our algorithm was tested on sets containing 1M simulated aDNA fragments using as endogenous genome an early modern humans (top), Neanderthals (middle) and a Denisovan(bottom). We tested our algorithm both with simulated double-stranded (left) and single-stranded (right) protocols. The dotted black line represents a perfect prediction.

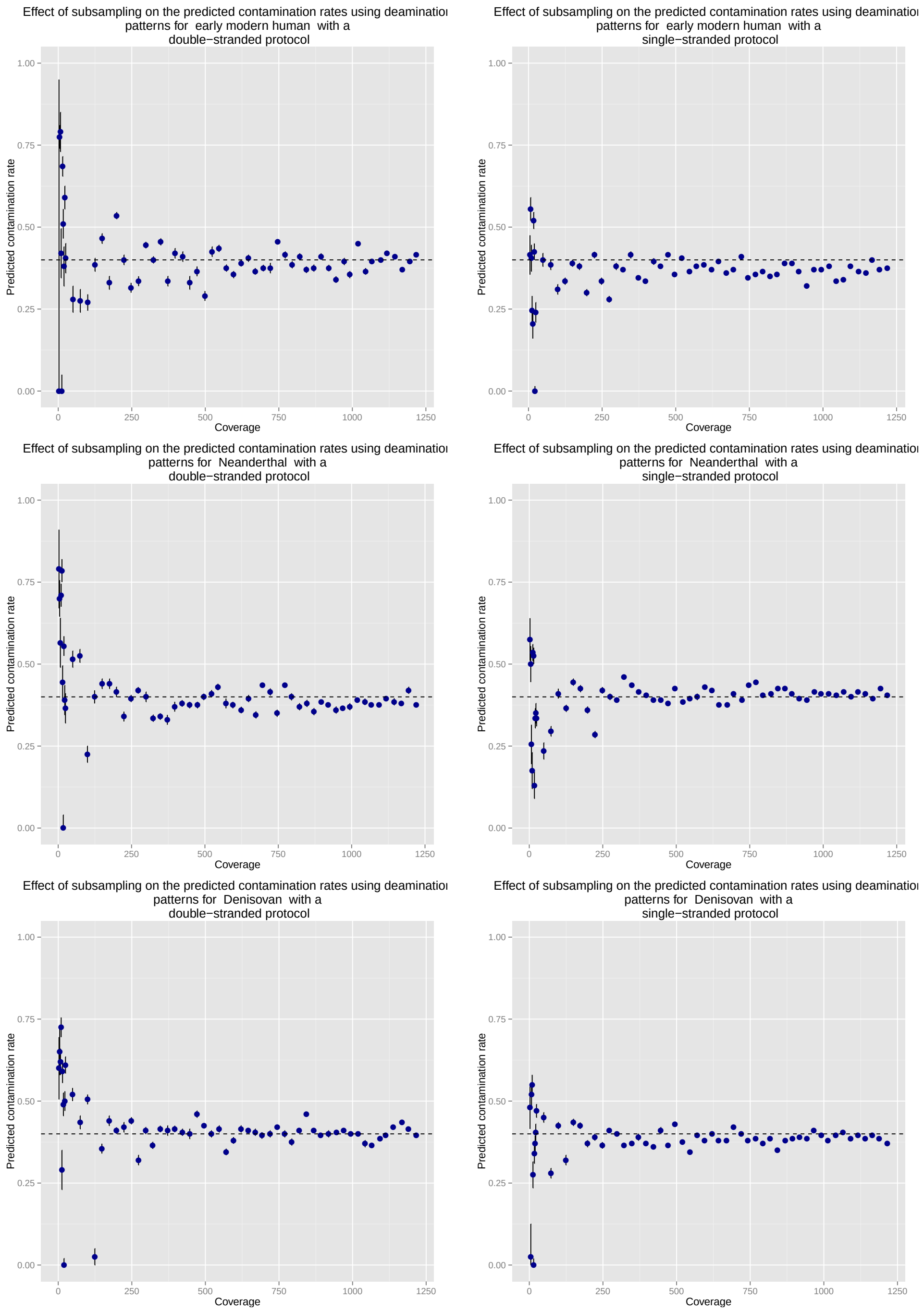


Figure S15: Simulated contamination rates using subsampled sets from a 1M fragment dataset where the original contamination rate was 40% (dotted black line) versus the predicted ones using deamination rates alone. The vertical black lines represent the boundaries of the 95% confidence interval.

### S2.3.4 Biases affecting the prior contamination estimate based on deamination

The contamination prior obtained for the first iteration relies on measuring deamination patterns for the endogenous fragments versus the entire dataset (see main text). One of the approaches to infer endogenous deamination rates is by conditioning on one end of the fragment being deaminated and measuring deamination rates for the other end, a previously described methodology (see [11]). However, while we use this approach to obtain an initial mitochondrial contamination estimate, it can be used for contamination estimates in itself under the following assumptions:

- There is a sufficient number of fragments to allow estimates of deamination rates
- Deamination rates of the endogenous fragments are sufficiently high. Having endogenous fragments with no deamination patterns will not yield an accurate estimate
- The aDNA fragments from the present-day humans that contaminate the sample are not themselves deaminated
- The rates of deamination of the 5' end of the fragment are independent of the rates of deamination of the 3' end and vice-versa

#### Impact of low deamination rates

To measure the impact of having low deamination rates, we repeated simulations by adding various rates of deamination for the endogenous fragments for simulated datasets with 50% contamination. We ran schmutzi's contamination estimate based on deamination patterns on our simulations. Our results presented in Table S19 show that a minimum deamination rate of 5-10% at least one end of the fragment is required to have a contamination estimate within 2-3% of the simulated contamination rate if 1M fragments are used. When a small number of fragments are used (100k), higher rates (40% and above) of deamination are required to obtain a reliable contamination estimate. At intermediate data sizes (500k), rates of deamination upwards of 15% are needed to obtain a reliable contamination estimate.

#### Impact of deamination for contaminating fragments

To measure the impact of various rates of deamination for the contaminant fragments, deamination was added to the simulated contaminant fragments. A contamination rate of 50% was used for our simulation sets 1M fragments for various rates of deamination for both the endogenous and contaminant fragments. Schmutzi's contamination estimate was used on those datasets. Our results show (see Table S20) that even a small amounts of deamination for the contaminant can lead to an underestimate. This effect less pervasive if the endogenous fragments have high levels of deamination or if the contaminant has very low levels of deamination.

#### Independence tests for deamination on each end

The contamination estimate based on deamination relies on measuring endogenous deamination rates and plotting the posterior probability for a non-informative contamination prior. Diagnostic positions cannot always be used

for measuring endogenous deamination rates for aDNA data. Therefore, our algorithm needs to condition on having one deaminated base on one end to measure endogenous deamination rates on the other and vice-versa. One underlying assumption is that deamination on one end is independent of deamination the other end. We sought to determine whether deamination rates on either end of the fragment were independent of deamination on the other end. We measured deamination rates on the 5' end conditioning on whether the 3' end was deaminated (C→T) or not (C→C). The converse was also measured. We evaluated subsets of the Altai Neanderthal [14], the Denisovan individual [22], the Loschbour individual [3], the Afontova Gora and Mal'ta genomes [29] (see Table S21). We ran a $\chi^2$ test on a two by two contingency table with one degree of freedom to test whether deamination on one end was independent of deamination on the other end. For all samples, except the Altai, the p-value was not sufficiently low to the point of concluding that deamination on one end is linked to deamination on the other. However, it should be noted that this is an assumption used by our algorithm and, if this assumption is incorrect and endogenous deamination rates are overestimated, an overestimate of the actual contamination rate will ensue.

| deamination | subsampling fraction | | | | | | | | | |
|---|---|---|---|---|---|---|---|---|---|---|
| rates (%) | 0.1 | 0.2 | 0.3 | 0.4 | 0.5 | 0.6 | 0.7 | 0.8 | 0.9 | 1 |
| 1 | 99.5±0.5 | 98.5±0.5 | 74.0±2.5 | 64.5±1.5 | 56.0±3.0 | 47.0±2.5 | 63.5±2.0 | 64.5±2.0 | 73.0±1.0 | 70.5±1.0 |
| 5 | 52.5±2.5 | 62.0±1.5 | 56.5±1.5 | 55.5±1.5 | 54.0±1.0 | 57.5±1.0 | 55.5±1.0 | 58.0±0.5 | 55.5±0.5 | 55.5±0.5 |
| 10 | 65.0±1.0 | 63.0±1.0 | 59.0±1.0 | 56.5±1.0 | 56.0±0.5 | 54.0±0.5 | 53.5±0.5 | 52.5±0.5 | 52.0±0.5 | 52.0±0.5 |
| 15 | 50.0±1.0 | 52.5±0.5 | 53.5±0.5 | 53.5±0.5 | 52.5±0.5 | 52.5±0.5 | 52.5±0.5 | 53.5±0.5 | 51.5±0.5 | 51.0±0.5 |
| 20 | 54.5±1.0 | 55.0±1.0 | 55.5±0.5 | 55.0±0.5 | 55.5±0.5 | 54.5±0.5 | 54.0±0.5 | 54.0±0.5 | 54.5±0.5 | 54.0±0.5 |
| 25 | 51.5±1.0 | 54.5±1.0 | 54.0±0.5 | 53.5±0.5 | 53.5±0.5 | 52.5±0.5 | 52.5±0.5 | 52.0±0.5 | 51.5±0.5 | 52.0±0.5 |
| 30 | 53.0±1.0 | 52.0±0.5 | 50.5±0.5 | 50.0±0.5 | 50.0±0.5 | 50.0±0.5 | 50.0±0.5 | 49.5±0.5 | 49.0±0.5 | 49.5±0.5 |
| 35 | 54.5±0.5 | 52.5±0.5 | 51.0±0.5 | 51.5±0.5 | 52.5±0.5 | 52.0±0.5 | 52.5±0.5 | 52.5±0.5 | 52.5±0.5 | 53.0±0.5 |
| 40 | 51.5±0.5 | 52.0±0.5 | 52.0±0.5 | 52.0±0.5 | 52.0±0.5 | 52.5±0.5 | 52.5±0.5 | 52.0±0.5 | 52.0±0.5 | 52.5±0.5 |
| 45 | 50.0±0.5 | 51.5±0.5 | 52.0±0.5 | 52.0±0.5 | 52.5±0.5 | 52.5±0.5 | 52.5±0.5 | 52.5±0.5 | 52.0±0.5 | 52.5±0.5 |
| 50 | 52.0±0.5 | 52.5±0.5 | 52.0±0.5 | 52.0±0.5 | 52.0±0.5 | 52.0±0.5 | 52.0±0.5 | 52.0±0.5 | 52.0±0.5 | 52.0±0.5 |
| 55 | 50.5±0.5 | 52.5±0.5 | 53.0±0.5 | 53.0±0.5 | 53.0±0.5 | 53.0±0.5 | 53.0±0.5 | 53.0±0.5 | 53.0±0.5 | 53.5±0.5 |
| 60 | 53.0±0.5 | 53.0±0.5 | 53.0±0.5 | 53.5±0.5 | 53.0±0.5 | 53.0±0.5 | 53.0±0.5 | 53.0±0.5 | 53.0±0.5 | 53.0±0.5 |
| 65 | 53.5±0.5 | 53.0±0.5 | 52.0±0.5 | 52.5±0.5 | 52.5±0.5 | 52.5±0.5 | 52.0±0.5 | 52.5±0.5 | 52.5±0.5 | 52.5±0.5 |
| 70 | 53.5±0.5 | 53.0±0.5 | 53.0±0.5 | 53.0±0.5 | 53.0±0.5 | 53.0±0.5 | 53.0±0.5 | 53.0±0.5 | 53.0±0.5 | 53.0±0.5 |
| 75 | 53.0±0.5 | 52.5±0.5 | 53.0±0.5 | 53.0±0.5 | 53.0±0.5 | 53.0±0.5 | 53.0±0.5 | 53.0±0.5 | 53.0±0.5 | 53.0±0.5 |
| 80 | 53.5±0.5 | 54.0±0.5 | 53.5±0.5 | 53.5±0.5 | 53.5±0.5 | 53.5±0.5 | 53.5±0.5 | 53.5±0.5 | 53.5±0.5 | 53.0±0.5 |
| 85 | 52.5±0.5 | 53.0±0.5 | 53.0±0.5 | 53.0±0.5 | 53.0±0.5 | 53.0±0.5 | 53.0±0.5 | 53.0±0.5 | 53.0±0.5 | 53.0±0.5 |
| 90 | 53.0±0.5 | 53.0±0.5 | 53.0±0.5 | 53.5±0.5 | 53.5±0.5 | 53.5±0.5 | 53.5±0.5 | 53.5±0.5 | 53.0±0.5 | 53.5±0.5 |
| 95 | 53.5±0.5 | 53.5±0.5 | 53.5±0.5 | 53.5±0.5 | 53.5±0.5 | 53.5±0.5 | 53.5±0.5 | 53.5±0.5 | 53.5±0.5 | 53.5±0.5 |

Table S19: Contamination estimate based on deamination patterns as a fraction of amount of data and deamination rates for datasets with a simulated contamination rate of 50%. The "subsampling fraction", is the fraction of fragments from the original 1M dataset that were used in the subsample.

| endogenous deamination rates (%) | contaminant deamination rates (%) | | | | | | | | | |
|---|---|---|---|---|---|---|---|---|---|---|
| | 0 | 1 | 2 | 3 | 4 | 5 | 6 | 7 | 8 | 9 |
| 1 | 70.5±1.0 | 73.0±0.5 | 64.5±0.5 | 61.0±0.5 | 58.0±0.5 | 54.0±0.5 | 53.5±0.5 | 49.0±0.5 | 53.0±0.5 | 50.5±0.5 |
| 5 | 55.5±0.5 | 38.0±0.5 | 30.5±0.5 | 28.0±1.0 | 30.5±1.0 | 23.5±0.5 | 26.5±0.5 | 22.0±0.5 | 19.5±0.5 | 26.0±0.5 |
| 10 | 52.0±0.5 | 32.0±0.5 | 32.0±0.5 | 15.5±1.0 | 21.5±0.5 | 18.0±0.5 | 19.5±0.5 | 11.5±0.5 | 13.5±1.0 | 12.5±0.5 |
| 15 | 51.0±0.5 | 30.5±0.5 | 32.0±0.5 | 22.5±0.5 | 21.5±0.5 | 15.5±0.5 | 15.5±0.5 | 13.5±0.5 | 14.5±0.5 | 10.0±0.5 |
| 20 | 54.0±0.5 | 38.0±0.5 | 34.0±0.5 | 28.5±0.5 | 28.0±0.5 | 27.0±0.5 | 21.0±0.5 | 19.0±0.5 | 17.5±0.5 | 14.0±0.5 |
| 25 | 52.0±0.5 | 39.0±0.5 | 33.0±0.5 | 31.5±0.5 | 29.5±0.5 | 26.0±0.5 | 23.0±0.5 | 21.0±0.5 | 14.5±0.5 | 14.5±0.5 |
| 30 | 49.5±0.5 | 41.0±0.5 | 37.5±0.5 | 34.0±0.5 | 30.5±0.5 | 29.5±0.5 | 26.0±0.5 | 23.0±0.5 | 19.5±0.5 | 20.5±0.5 |
| 35 | 53.0±0.5 | 43.5±0.5 | 41.5±0.5 | 38.0±0.5 | 34.5±0.5 | 34.0±0.5 | 31.0±0.5 | 29.0±0.5 | 25.5±0.5 | 23.0±0.5 |
| 40 | 52.5±0.5 | 44.0±0.5 | 41.0±0.5 | 39.5±0.5 | 37.0±0.5 | 34.5±0.5 | 32.0±0.5 | 30.5±0.5 | 28.0±0.5 | 26.0±0.5 |
| 45 | 52.5±0.5 | 45.0±0.5 | 42.5±0.5 | 41.5±0.5 | 38.5±0.5 | 36.5±0.5 | 36.0±0.5 | 34.0±0.5 | 32.0±0.5 | 29.5±0.5 |
| 50 | 52.0±0.5 | 45.0±0.5 | 43.0±0.5 | 41.0±0.5 | 39.5±0.5 | 38.5±0.5 | 35.5±0.5 | 35.0±0.5 | 33.0±0.5 | 31.5±0.5 |
| 55 | 53.5±0.5 | 46.5±0.5 | 45.0±0.5 | 43.0±0.5 | 41.0±0.5 | 40.0±0.5 | 38.0±0.5 | 36.5±0.5 | 35.5±0.5 | 33.5±0.5 |
| 60 | 53.0±0.5 | 46.5±0.5 | 45.5±0.5 | 43.5±0.5 | 42.0±0.5 | 40.0±0.5 | 39.0±0.5 | 37.0±0.5 | 36.0±0.5 | 34.0±0.5 |
| 65 | 52.5±0.5 | 47.5±0.5 | 45.5±0.5 | 44.0±0.5 | 43.0±0.5 | 41.5±0.5 | 40.0±0.5 | 38.5±0.5 | 37.5±0.5 | 36.0±0.5 |
| 70 | 53.0±0.5 | 47.5±0.5 | 46.0±0.5 | 45.0±0.5 | 43.5±0.5 | 42.5±0.5 | 41.0±0.5 | 39.5±0.5 | 38.0±0.5 | 37.5±0.5 |
| 75 | 53.0±0.5 | 48.0±0.5 | 47.0±0.5 | 46.0±0.5 | 44.0±0.5 | 43.0±0.5 | 41.5±0.5 | 40.5±0.5 | 39.5±0.5 | 38.5±0.5 |
| 80 | 53.0±0.5 | 48.5±0.5 | 47.5±0.5 | 46.5±0.5 | 45.5±0.5 | 43.5±0.5 | 43.0±0.5 | 41.5±0.5 | 41.0±0.5 | 39.5±0.5 |
| 85 | 53.0±0.5 | 49.0±0.5 | 47.5±0.5 | 46.5±0.5 | 45.5±0.5 | 44.0±0.5 | 43.0±0.5 | 42.0±0.5 | 40.5±0.5 | 39.5±0.5 |
| 90 | 53.5±0.5 | 49.0±0.5 | 48.0±0.5 | 47.0±0.5 | 46.0±0.5 | 45.0±0.5 | 44.0±0.5 | 42.5±0.5 | 41.5±0.5 | 40.5±0.5 |
| 95 | 53.5±0.5 | 49.5±0.5 | 48.5±0.5 | 47.5±0.5 | 46.0±0.5 | 45.5±0.5 | 44.5±0.5 | 43.5±0.5 | 42.0±0.5 | 41.0±0.5 |

Table S20: Effect of having deamination for contaminant fragments on the contamination estimate at various deamination rates for endogenous fragments. The original simulated contamination rate was 50%.

| sample type | deamination rates for | | | | $\chi^2$ test | | | | contamination estimate using deamination patterns |
|---|---|---|---|---|---|---|---|---|---|
| | 5'end | | 3'end [9] | | 5'end | | 3'end | | |
| | 3': C→T | 3': C→C | 5': C→T | 5': C→C | $\chi^2$ | p-value | $\chi^2$ | p-value | |
| Afontova Gora | 0.0510778 | 0.0424544 | 0.0385227 | 0.0319476 | 7.2993 | 0.006898 | 6.4212 | 0.01128 | 0.0±0.5 |
| Altai Neanderthal | 0.079841 | 0.0677685 | 0.305404 | 0.269203 | 37.0381 | 1.158e-09 | 33.8252 | 6.029e-09 | 11.5±0.5 |
| Denisovan | 0.0933588 | 0.0899034 | 0.515545 | 0.50517 | 2.5505 | 0.1103 | 2.6116 | 0.1061 | 2.5±1.0 |
| Loschbour | 0.0846782 | 0.0784448 | 0.372735 | 0.353486 | 5.0888 | 0.02408 | 5.5029 | 0.01898 | 4.5±1.0 |
| Mal'ta | 0.0297943 | 0.0291137 | 0.0337034 | 0.0329365 | 0.111 | 0.7391 | 0.2124 | 0.6449 | 0.0±0.5 |

Table S21: Independence of deamination rates for 5' and 3' ends of aDNA fragments for various empirical datasets with low levels of present-day human contamination. Two by two contingency $\chi^2$ tests were used with 1 degree of freedom. The absence of independence between deamination rates at both ends for the Altai Neanderthal leads to an overestimate of the endogenous deamination rate and, as a consequence, of contamination.

### S2.3.5 Contamination estimate based on divergent bases

Once the endogenous consensus call is completed, contamination rates can be computed using this consensus and a set of putative mitochondrial contaminants. This process is repeated until a stable contamination rate is reached and the final rate is produced. Similarly to the section above, we sought to measure the correlation between this final contamination rate and the predefined target contamination rate used in the simulated data. The target contamination rate for the simulations was calculated as the fraction of bases pertaining to the contaminant over the total sum.

#### Full datasets

As mentioned in the methods, users can run the prediction with or without the inclusion of the predicted contaminant as a record in the database of putative contaminants. We ran schmutzi on our six types of previously described datasets of 1M fragments. We ran schmutzi once with the inclusion of the predicted contaminant and once again without this option.

**Full datasets: Using the records in the database only**

Using solely the records in the database described in Section S1, the contamination rate was computed once the algorithm reached convergence. This option always results in an underestimate of the true contamination rate as some sites on the mitochondrial genome will not be considered due to natural divergence between the actual contaminant and the closest record in the database. We plotted the correlation between the simulated contamination rate and the predicted one (see Figure S16).

For both archaic hominins, due to the large numbers of segregating sites compared to the contamination source, the effect of this underestimate is minimal as the contamination estimate is highly correlated with the simulated one. For the EMH, due to the smaller divergence between the contaminant and endogenous genomes, very few sites are considered and the effect of the underestimate is more prominent, especially at higher contamination rates. In the following section, we show that these more difficult targets can be predicted by including the inferred contaminant in the database of putative contaminant genomes.

**Full datasets: Including the predicted contaminant**

We re-ran our program on the same datasets used in the previous section with the inclusion of the predicted contaminant. The correlation between the simulated contamination rate and the predicted one was plotted (see Figure S17). The program performed well for both archaic hominin genomes, similarly to the previous section, as high divergence between the contaminant and the endogenous genomes provide an easy target for contamination estimates. For the EMH, the underestimate seen in the previous section is corrected for using the predicted contaminant as information. However, this approach does not perform well at very low levels of contamination, as adequate characterization of the contaminant genome is not feasible.

#### Subsampled datasets

To measure the robustness of our algorithm to low coverage samples, the dataset with 40% contamination rate was subsampled at rates ranging from 0.5 down to 0.01. Two distinct approaches were taken when rerunning schmutzi on the resulting datasets. The first involved the default behavior of predicting the

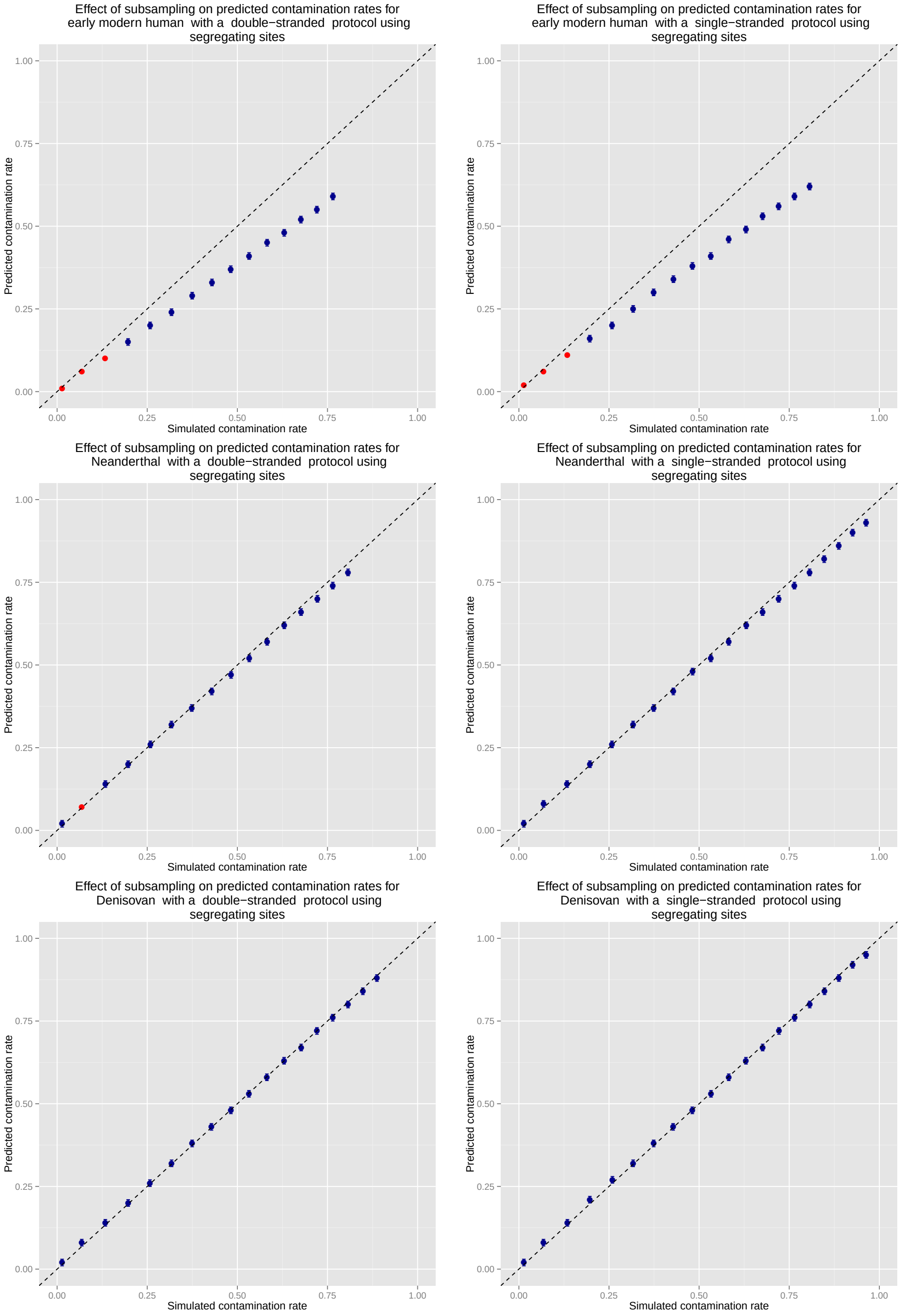


Figure S16: Simulated contamination rate versus the predicted one for datasets containing 1M fragments each. The endogenous genome used was either an early modern human (top), a Neanderthal (middle) or a Denisovan (bottom) and the simulated aDNA damage pattern was either double-stranded (left) or single-stranded (right). The data points where our program stopped after the first iteration due to lack of contaminant fragments to characterize are marked in red. As mentioned in the previous sections, for the EMH at high levels of contamination, our algorithm did not converge.

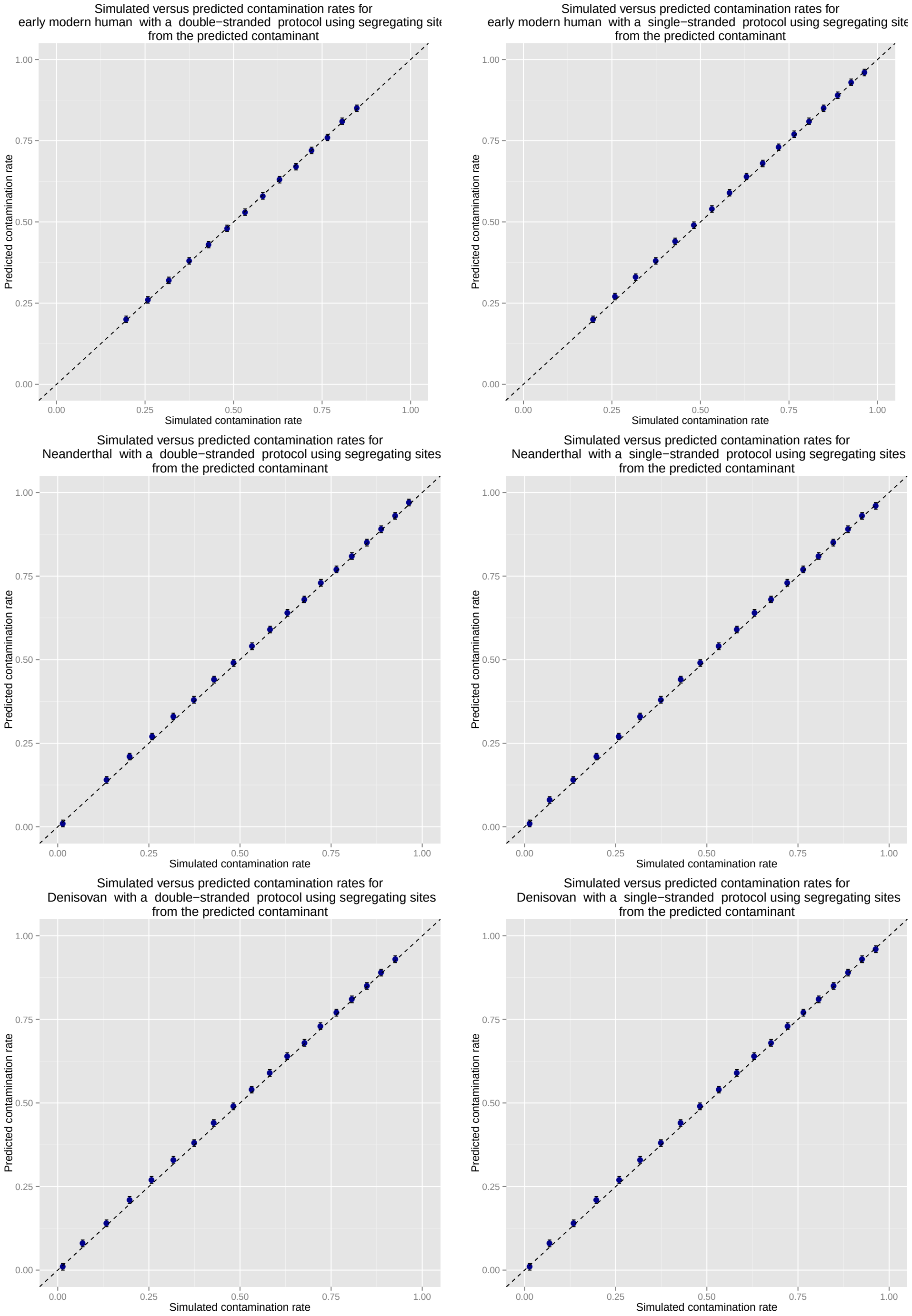


Figure S17: Simulated contamination rate versus the predicted one using the predicted contaminant as putative contaminant source for datasets containing 1M fragments each. The endogenous genome used was either an early modern human (top), a Neanderthal (middle) or a Denisovan (bottom) and the simulated aDNA damage pattern was double-stranded (left) or single-stranded (right). As for the previous graphs, the data points where schmutzi did not converge are omitted which mostly occur with a EMH as endogenous with either too little or too much contamination.

endogenous genome and scanning the contaminant database to estimate contamination rates. However, for very low coverage samples, getting an accurate resolution of the endogenous mitochondrial genome is often difficult. Sometimes, investigators have access to an endogenous mitochondrial genome that can serve as a close proxy (e.g. a different Neanderthal for a Neanderthal sample, a mitochondrial genome from the same haplogroup for early modern humans) to determine whether this low coverage sample is heavily contaminated. This is useful to prioritize which extractions are the most promising and should be further sequenced. The second approach therefore involved taking the endogenous consensus from the original high coverage dataset and using it as the endogenous genome. This latter approach has the advantage of being highly robust to low coverage but requires a well-characterized endogenous genome or a very close proxy.

**Subsampled datasets: Using a consensus from the dataset itself**

Using the default methodology, we inferred contamination rates from the predicted endogenous genome and putative contaminants in the database. As the simulated contamination rate was known, we plotted the final contamination rates as a function of coverage (see Figure S18). For both archaics, the contamination estimate is reliable from about 100X or 200X coverage for the single-stranded and double-stranded rates of deamination respectively. For the EMH, the contamination estimate remains an underestimate since schmutzi does not use the predicted contaminant as a putative source of contamination with default parameters.

**Subsampled datasets: Using a consensus from a higher quality source**

In the previous section, we saw that schmutzi performed well at coverages levels that are routinely seen in aDNA projects due to the relative abundance of the mitochondrial DNA compared to nuclear [10]. However, in certain studies, the relative amount of non-bacterial DNA is relatively small leading to extracts yielding low coverage across the mitochondrial genome (e.g. less than 50X). In those cases, neither approach to estimate contamination by deamination patterns or by endogenous consensus calling followed by comparison to a database yielded accurate estimates.

A hurdle in predicting contamination using low coverage samples is the inability to accurately call the endogenous mitochondrial genome. However, it is possible that researchers have access to a higher quality mitochondrial genome from the same individual (obtained using mitochondrial capture for example) and wish to prioritize which extractions are most promising to fully sequence the nuclear genome. It is also possible to determine from which clade or haplogroup the individual being sequenced belongs to therefore providing a close proxy. Our results show that if a research group has access to a high quality mitochondrial genome from a close proxy, contamination can be estimated even at low coverage. This approach can be useful if a group prepared a new library from a Neanderthal extract and wishes to estimate contamination despite low coverage across the mitochondrial genome. Knowing that the sample pertains to a Neanderthal entails that a high quality mitochondrial genome from a different Neanderthal can be used as substitute. The contamination rate could therefore be estimated for the new low coverage library. We supplied our contamination estimator with the endogenous genome predicted from the original 1M datasets. Our results show that our estimates are accurate for even very low coverage samples. (see Figure S19).

For both archaics hominins, the estimate is close to the actual simulated rate even at low coverage. For the EMH, the underestimate due to the exclusion of the contaminant is still noticeable however, the estimate offers greater robustness to low coverage rates compared to simply estimating contamination using the endogenous consensus from the sample itself.

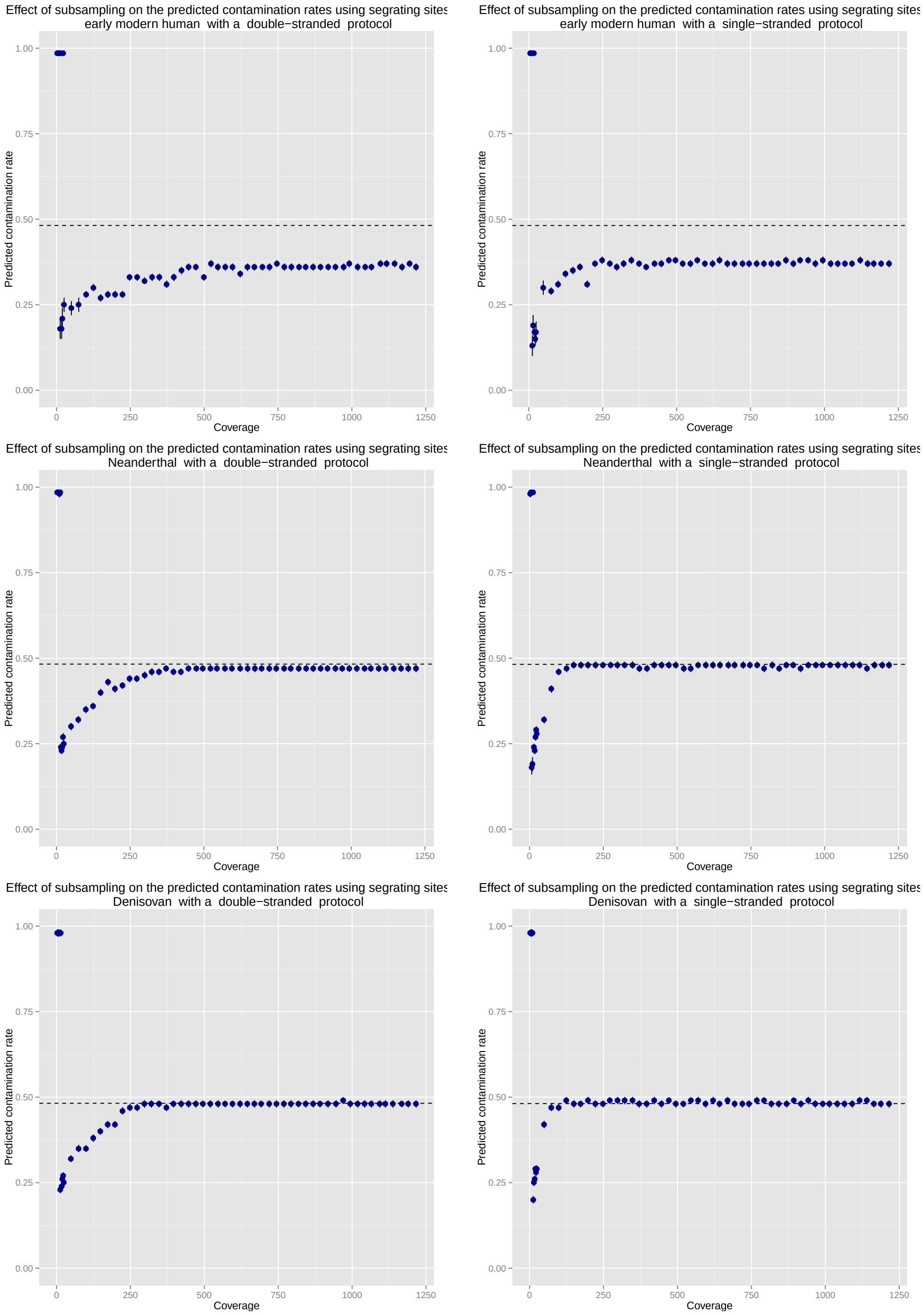


Figure S18: Predicted contamination rates at various rates of coverage using schmutzi with default parameters. The endogenous genome used was either an early modern human (top), a Neanderthal (middle) or a Denisovan (bottom) and the simulated aDNA damage pattern was double-stranded (left) or single-stranded (right). The black dotted line corresponds to the simulated contamination rate.

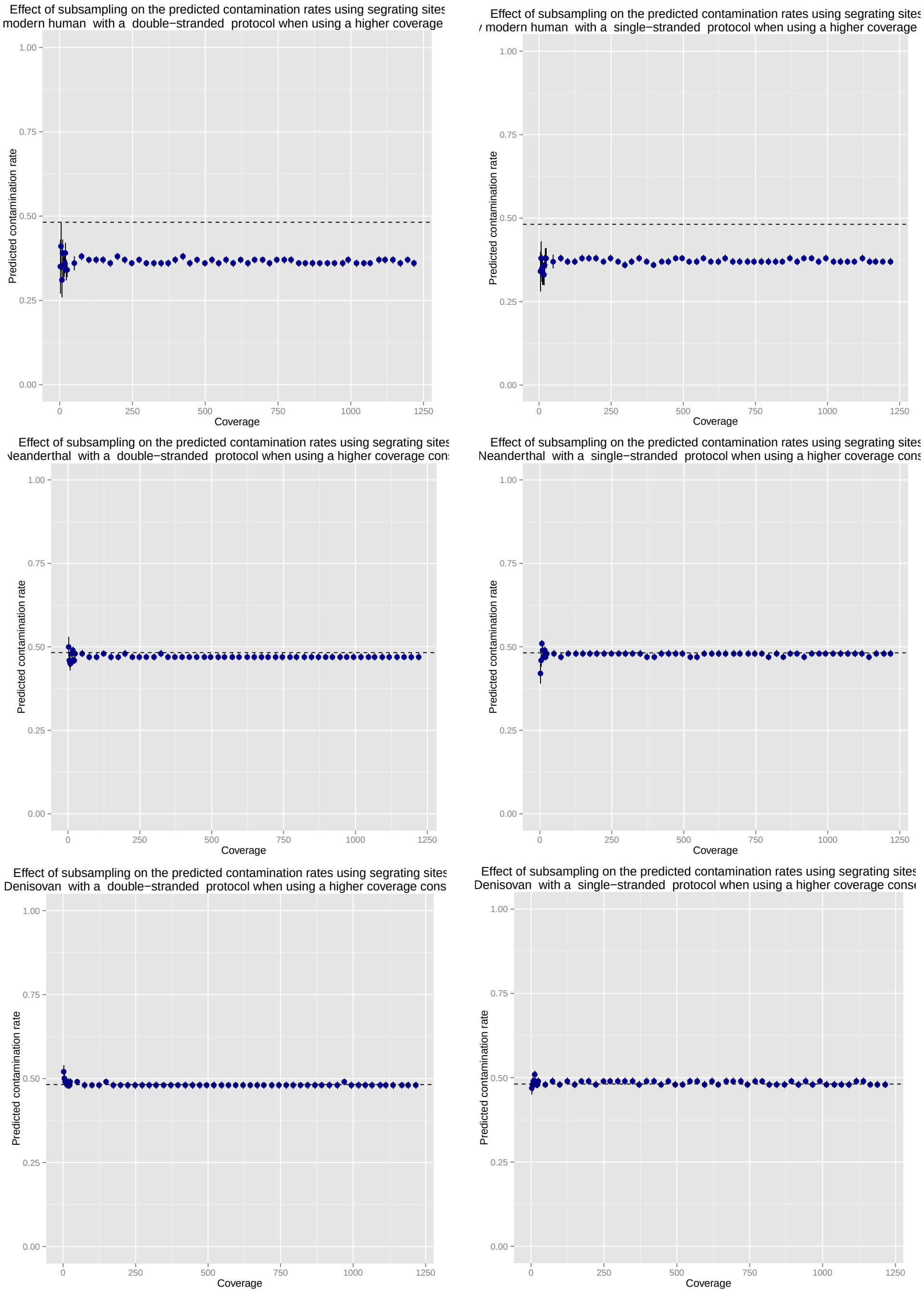


Figure S19: Predicted contamination rates at various rates of coverage using schmutzi with default parameters but with the endogenous genome inferred from the original set from which the fragments were subsampled. The endogenous genome used was either an early modern human (top), a Neanderthal (middle) or a Denisovan (bottom) and the simulated aDNA damage pattern was double-stranded (left) or single-stranded (right). The black dotted line corresponds to the simulated contamination rate.

### S2.3.6 Comparison to existing methods

Using the maximum likelihood method previously described in the literature [7], a contamination estimate for each simulated set of 1M fragment was computed. Our results measure the correlation between the simulated contamination rate and the one obtained using this method (see Figure S20). As the contaminating mitochondrial genome was known, the program was run once where this genome was used as the contamination source. The program was run again using the closest mitochondrial genome to the contaminant one in the 311 database records provided in the original description of the method. One issue with this maximum likelihood method is the inability to quantify the three main sources of uncertainty: sequencing errors, deamination and mismappings. The result is an estimate that misses the simulated contamination rate at lower and higher levels of contamination. An underestimate of the error rate leads to an overestimate of the contamination rate and vice-versa. Mitigating measures against deamination can be taken like trimming the ends of fragments or restricting the analysis to transversions only. However these approaches suffer from residual deamination in the middle of the fragments and reduction of ascertainment power respectively. The impact of mismappings could be mitigated by filtering for fragments with high mapping quality but this does not guarantee that every fragment is correctly mapped to its original position.

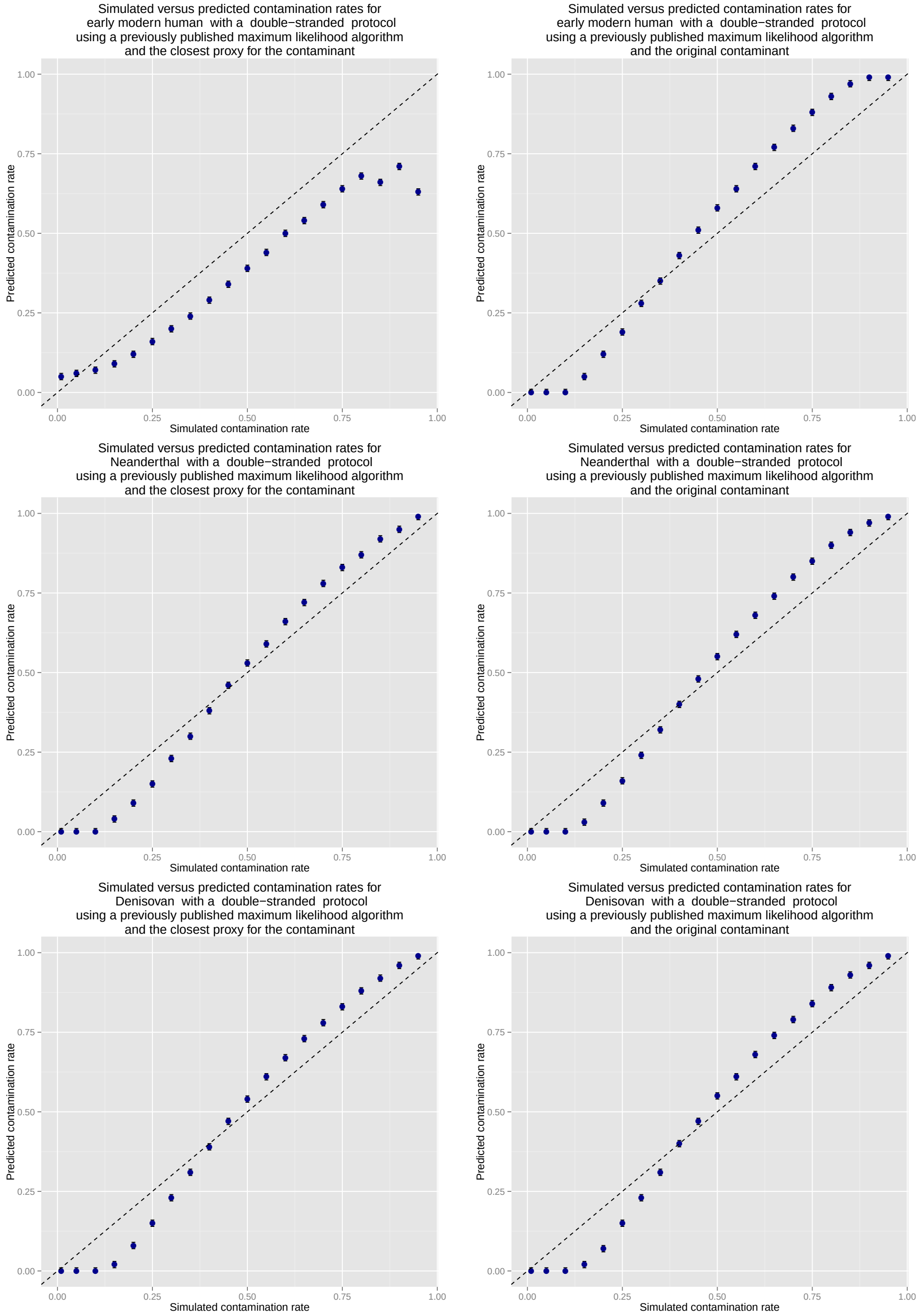


Figure S20: Predicted contamination rates at various rates of coverage using a previously described maximum likelihood method. The method was tested on sets containing 1M simulated aDNA fragments using as endogenous genome an early modern humans (top), Neanderthals (middle) and a Denisovan(bottom). The method was used by including the closest record in the 311 mitochondrial genome database described in the method (left). To present the upper predictive limit, the actual contaminant used in the simulation was included (right). The dotted black line represents a perfect prediction.

### S2.3.7 Multiple contaminants

As seen in the methods section, the algorithm assumes a single contaminant nucleotide at a given position. If multiple human mitochondrial genomes from present-day humans with several divergent positions contaminate the ancient sample, positions where one of the contaminant genomes shares an allele with the endogenous one can lead to underestimates. The biggest hurdle for our algorithm is not the number of contaminant mitochondria but rather, the sharing of alleles with the endogenous genome.

We sought to evaluate how our estimates would be affected as a result of having more than one mitochondrial genomes that contaminate the endogenous sample. Simulations of 170k aDNA fragments with an early modern human and Neanderthals as endogenous samples were repeated using a mix of a second mitochondrial genome (GenBank ID: EU926618.1) in addition to the previously used contaminant mitochondrial genome (GenBank ID: KJ446110.1). This second contaminating mitochondrion had an edit distance of 21 to the first contaminant mitochondrial genome, comparable to the distance of the endogenous early modern human genome to the first contaminant mitochondrion (edit distance: 24) and the second one (edit distant: 21).

The mixture proportion of the two mitochondrial genomes ranged from 0% to 50% with steps of 10%. Single-stranded damage was added only to the endogenous material as described in the Methods section of the main text. Contamination rates varied from 0% to 90%. Contamination estimates were produced using the predicted contaminant bases for estimates starting from 20% contamination rate onwards as the prediction of the contaminant mitochondrial genome becomes feasible at this level (see Results in the main text).

Our results show that high mixture proportions of multiple contaminants do not affect our contamination estimates at low rates of contamination (<20%) for the early modern humans (see Figure S21A). At medium rates of contamination (>20% and <70%), an underestimate of about 8% can be seen at the highest rate of blending. At high levels of contamination (>70%) and at a higher mixture of multiple contaminants (40% and 50%), higher underestimates are seen. However, when the endogenous mitochondrial genome is that of a Neanderthal, the contamination estimates are more robust to higher mixtures of contaminant mitochondrial genomes (see Figure S21B). This robustness is due to the greater agreement between the two contaminant mitochondrial genomes relatively to the Neanderthal mitochondrial genome. The presence of multiple contaminant genomes also does not affect the estimates produced using deamination patterns (see Figure S21C and D).

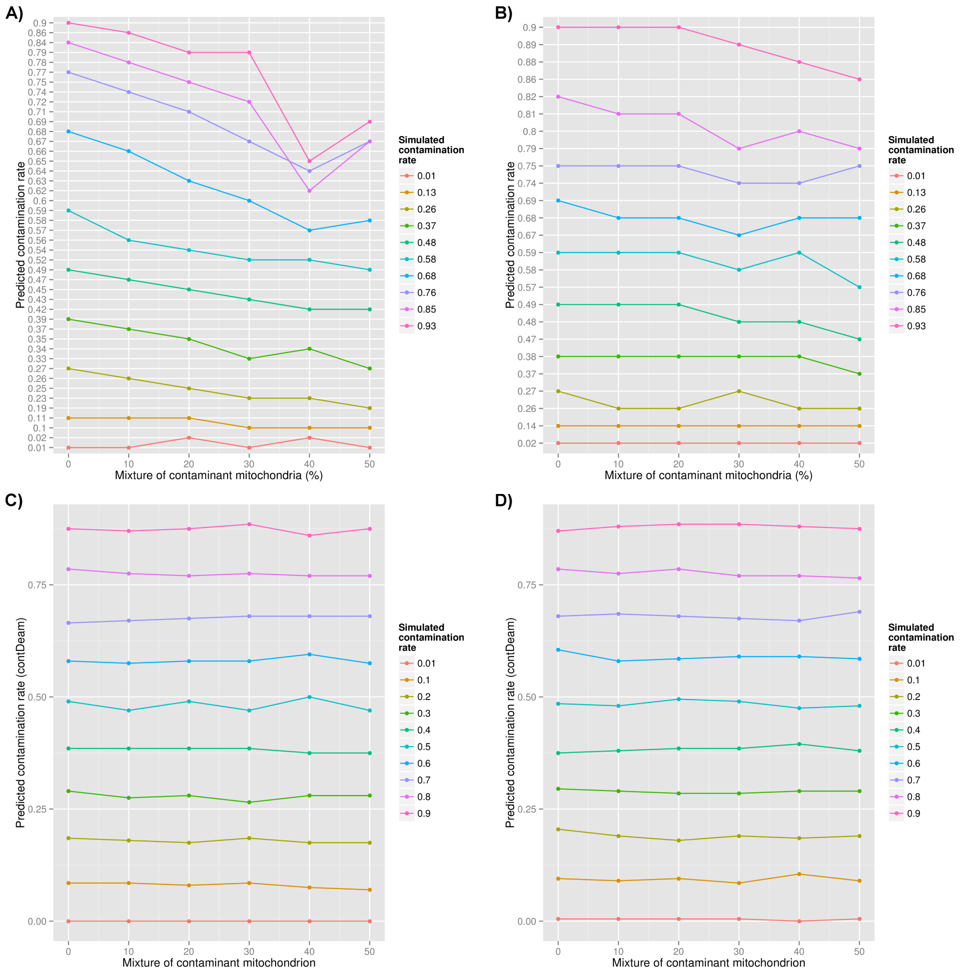


Figure S21: Effect of having multiple contaminant bases at various blends of contaminant mitochondrial genomes (x-axis) on the contamination estimate produced by schmutzi (y-axis) for different rates of simulated contamination (see color codes in the legend). The x-axis represents the proportion at which contaminant genomes were mixed while the colored lines represents the proportion at which these contaminant genomes were mixed with the endogenous one. A) Effect on the predicted contamination estimate of multiple contaminant genomes for an early modern human as the endogenous genome. B) Effect of multiple contaminants on contamination estimates using a Neanderthal mitochondrial genome as the endogenous sample. C) and D) Contamination estimates based on deamination patterns for the early modern human and Neanderthal genomes.

# References

[1] Li, H., Durbin, R.: Fast and accurate short read alignment with Burrows–Wheeler transform. Bioinformatics **25**(14), 1754–1760 (2009)

[2] Krause, J., Fu, Q., Good, J.M., Viola, B., Shunkov, M.V., Derevianko, A.P., Pääbo, S.: The complete mitochondrial DNA genome of an unknown hominin from southern Siberia. Nature **464**(7290), 894–897 (2010)

[3] Lazaridis, I., Patterson, N., Mittnik, A., Renaud, G., Mallick, S., Kirsanow, K., Sudmant, P.H., Schraiber, J.G., Castellano, S., *et al.*: Ancient human genomes suggest three ancestral populations for present-day Europeans. Nature **513**(7518), 409–413 (2014)

[4] Schubert, M., Ginolhac, A., Lindgreen, S., Thompson, J.F., Al-Rasheid, K.A., Willerslev, E., Krogh, A., Orlando, L.: Improving ancient DNA read mapping against modern reference genomes. BMC genomics **13**(1), 178 (2012)

[5] David, M., Dzamba, M., Lister, D., Ilie, L., Brudno, M.: SHRiMP2: sensitive yet practical short read mapping. Bioinformatics **27**(7), 1011–1012 (2011)

[6] Minoche, A.E., Dohm, J.C., Himmelbauer, H.: Evaluation of genomic high-throughput sequencing data generated on Illumina HiSeq and Genome Analyzer systems. Genome Biology **12**(11), 112 (2011)

[7] Fu, Q., Mittnik, A., Johnson, P.L., Bos, K., Lari, M., Bollongino, R., Sun, C., Giemsch, L., Schmitz, R., Burger, J., *et al.*: A revised timescale for human evolution based on ancient mitochondrial genomes. Current Biology **23**(7), 553–559 (2013)

[8] Fu, Q., Li, H., Moorjani, P., Jay, F., Slepchenko, S.M., Bondarev, A.A., Johnson, P.L., Aximu-Petri, A., Prüfer, K., de Filippo, C., *et al.*: Genome sequence of a 45,000-year-old modern human from western Siberia. Nature **514**(7523), 445–449 (2014)

[9] Noonan, J.P., Coop, G., Kudaravalli, S., Smith, D., Krause, J., Alessi, J., Chen, F., Platt, D., Pääbo, S., Pritchard, J.K., *et al.*: Sequencing and analysis of neanderthal genomic DNA. Science **314**(5802), 1113–1118 (2006)

[10] Green, R.E., Briggs, A.W., Krause, J., Prüfer, K., Burbano, H.A., Siebauer, M., Lachmann, M., Pääbo, S.: The Neandertal genome and ancient DNA authenticity. The EMBO Journal **28**(17), 2494–2502 (2009)

[11] Meyer, M., Fu, Q., Aximu-Petri, A., Glocke, I., Nickel, B., Arsuaga, J.L., Martinez, I., Gracia, A., de Castro, J.M., Carbonell, E., Paabo, S.: A mitochondrial genome sequence of a hominin from Sima de los Huesos. Nature **505**(7483), 403–406 (2014)

[12] Katoh, K., Standley, D.M.: MAFFT multiple sequence alignment software version 7: improvements in performance and usability. Molecular Biology and Evolution **30**(4), 772–780 (2013)

[13] Gansauge, M.-T., Meyer, M.: Selective enrichment of damaged DNA molecules for ancient genome sequencing. Genome Research **24**(9), 1543–1549 (2014)

[14] Prüfer, K., Racimo, F., Patterson, N., Jay, F., Sankararaman, S., Sawyer, S., Heinze, A., Renaud, G., Sudmant, P.H., de Filippo, C., *et al.*: The complete genome sequence of a Neanderthal from the Altai mountains. Nature **505**(7481), 43–49 (2014)

[15] Felsenstein, J.: Evolutionary trees from DNA sequences: a maximum likelihood approach. Journal of Molecular Evolution **17**(6), 368–376 (1981)

[16] Felsenstein, J.: PHYLIP - Phylogeny Inference Package (Version 3.2). Cladistics **5**, 164–166 (1989)

[17] Löytynoja, A., Goldman, N.: An algorithm for progressive multiple alignment of sequences with insertions. Proceedings of the National Academy of Sciences of the United States of America **102**(30), 10557–10562 (2005)

[18] Rohland, N., Hofreiter, M.: Ancient DNA extraction from bones and teeth. Nature Protocols **2**(7), 1756–1762 (2007)

[19] Gansauge, M.-T., Meyer, M.: Single-stranded DNA library preparation for the sequencing of ancient or damaged DNA. Nature Protocols **8**(4), 737–748 (2013)

[20] Kircher, M., Sawyer, S., Meyer, M.: Double indexing overcomes inaccuracies in multiplex sequencing on the Illumina platform. Nucleic acids research **40**(1), 3–3 (2012)

[21] Ruffalo, M., LaFramboise, T., Koyutürk, M.: Comparative analysis of algorithms for next-generation sequencing read alignment. Bioinformatics **27**(20), 2790–2796 (2011)

[22] Meyer, M., Kircher, M., Gansauge, M.-T., Li, H., Racimo, F., Mallick, S., Schraiber, J.G., Jay, F., Prüfer, K., de Filippo, C., *et al.*: A high-coverage genome sequence from an archaic Denisovan individual. Science **338**(6104), 222–226 (2012)

[23] Van Oven, M., Kayser, M.: Updated comprehensive phylogenetic tree of global human mitochondrial DNA variation. Human Mutation **30**(2), 386–394 (2009)

[24] Kloss-Brandstätter, A., Pacher, D., Schönherr, S., Weissensteiner, H., Binna, R., Specht, G., Kronenberg, F.: HaploGrep: a fast and reliable algorithm for automatic classification of mitochondrial DNA haplogroups. Human Mutation **32**(1), 25–32 (2011)

[25] Behar, D.M., van Oven, M., Rosset, S., Metspalu, M., Loogväli, E.-L., Silva, N.M., Kivisild, T., Torroni, A., Villems, R.: A "Copernican" reassessment of the human mitochondrial DNA tree from its root. The American Journal of Human Genetics **90**(4), 675–684 (2012)

[26] Haak, W., Lazaridis, I., Patterson, N., Rohland, N., Mallick, S., Llamas, B., Brandt, G., Nordenfelt, S., Harney, E., Stewardson, K., Fu, Q., Mittnik, A., Banffy, E., Economou, C., Francken, M., Friederich, S., Pena, R.G., Hallgren, F., Khartanovich, V., Khokhlov, A., Kunst, M., Kuznetsov, P., Meller, H., Mochalov, O., Moiseyev, V., Nicklisch, N., Pichler, S.L., Risch, R., Rojo Guerra, M.A., Roth, C., Szecsenyi-Nagy, A., Wahl, J., Meyer, M., Krause, J., Brown, D., Anthony, D., Cooper, A., Alt, K.W., Reich, D.: Massive migration from the steppe was a source for Indo-European languages in Europe. Nature (2015)

[27] Seguin-Orlando, A., Korneliussen, T.S., Sikora, M., Malaspinas, A.S., Manica, A., Moltke, I., Albrechtsen, A., Ko, A., Margaryan, A., Moiseyev, V., Goebel, T., Westaway, M., Lambert, D., Khartanovich, V., Wall, J.D., Nigst, P.R., Foley, R.A., Lahr, M.M., Nielsen, R., Orlando, L., Willerslev, E.: Genomic structure in Europeans dating back at least 36,200 years. Science **346**(6213), 1113–1118 (2014)

[28] Skoglund, P., Northoff, B.H., Shunkov, M.V., Derevianko, A.P., Pääbo, S., Krause, J., Jakobsson, M.: Separating endogenous ancient DNA from modern day contamination in a Siberian Neandertal. Proceedings of the National Academy of Sciences **111**(6), 2229–2234 (2014)

[29] Raghavan, M., Skoglund, P., Graf, K.E., Metspalu, M., Albrechtsen, A., Moltke, I., Rasmussen, S., Stafford Jr, T.W., Orlando, L., Metspalu, E., et al.: Upper Palaeolithic Siberian genome reveals dual ancestry of Native Americans. Nature (2013)

[30] Robinson, J.T., Thorvaldsdóttir, H., Winckler, W., Guttman, M., Lander, E.S., Getz, G., Mesirov, J.P.: Integrative genomics viewer. Nature Biotechnology **29**(1), 24–26 (2011)

[31] Green, R.E., Malaspinas, A.-S., Krause, J., Briggs, A.W., Johnson, P.L., Uhler, C., Meyer, M., Good, J.M., Maricic, T., Stenzel, U., *et al.*: A complete Neandertal mitochondrial genome sequence determined by high-throughput sequencing. Cell **134**(3), 416–426 (2008)

[32] Burbano, H.A., Hodges, E., Green, R.E., Briggs, A.W., Krause, J., Meyer, M., Good, J.M., Maricic, T., Johnson, P.L., Xuan, Z., *et al.*: Targeted investigation of the Neandertal genome by array-based sequence capture. Science **328**(5979), 723–725 (2010)
